# Subnational inequalities and preventable gaps in drowning premature mortality and non-fatal health loss among population younger than 20 years

**DOI:** 10.3389/fpubh.2026.1917814

**Published:** 2026-08-28

**Authors:** Gengfeng Su, You Zeng, Wenyi Jin, Queran Lin, Shihao Wang, Pi Guo, Junyan Xi, Lian Liang, Jiajia Wang, Bingying Fan, Min Huang, Jingxuan Jia, Xiaoliang Huang, Xiao Lin

**Affiliations:** 1 Department of Epidemiology, Fuwai Hospital, National Center for Cardiovascular Diseases, Chinese Academy of Medical Sciences and Peking Union Medical College & School of Public Health Sun Yat-sen University, Beijing, China; 2 Department of Medical Statistics & Center for Health Information Research & Guangdong Key Laboratory of Medicine, School of Public Health, Sun Yat-sen University, Guangzhou, Guangdong, China; 3 Department of Women and Children Health Care, Guangzhou Baiyun District Maternal and Child Health Hospital, Guangzhou, China; 4 Department of Orthopaedics, Renmin Hospital of Wuhan University, Wuhan University, Wuhan, China; 5 Department of Primary Care and Public Health, Imperial College London, London, United Kingdom; 6 Clinical Research Center & Department of Emergency Medicine & Institute of Cardiopulmonary Cerebral Resuscitation, Sun Yat-Sen Memorial Hospital, Sun Yat-Sen University, Guangzhou, Guangdong, China; 7 Department of Preventive Medicine, Shantou University Medical College, Shantou, China; 8 The Affairs Center of Health Commission of Guangdong Province, Guangzhou, Guangdong, China; 9 Sun Yat-sen Global Health Institute, School of Public Health, Sun Yat-sen University, Guangzhou, Guangdong, China

**Keywords:** drowning burden, subnational injury epidemiology, frontier analysis, inequality analysis, premature mortality

## Abstract

**Background:**

Drowning remains a preventable cause of premature mortality among children and adolescents. National estimates can monitor progress, but may not identify subnational locations where risk remains high. We examined drowning burden in the population younger than 20 years in 2023, focusing on age patterns, national differences, subnational variation, and frontier gaps.

**Methods:**

We analyzed 1990–2023 population health estimates for drowning. Deaths, disability-adjusted life-years (DALYs), death rates, and DALY rates were summarized globally and across 204 countries and territories. Subnational DALY-rate analyses included 507 locations in 17 countries. We assessed age-specific patterns, rankings by DALY rate and the number of DALYs, subnational variation, sociodemographic inequality, and frontier gaps. Subnational frontier analyses included 389 locations in 14 countries with available estimates on socio-demographic index and DALY rates.

**Results:**

In 2023, drowning caused 127,762 deaths and 10.48 million DALYs in the population < 20 years. Death and DALY rates declined by 68.4% and 68.8% from 1990 to 2023, while years of life lost accounted for 99.86% of DALYs. The highest DALY rate occurred at ages 12–23 months. Rankings by DALY rate and by the number of DALYs identified different national and subnational priorities. Subnational analyses showed large within-country variation. At the national level, positive frontier gaps were observed in 199 of 204 countries and territories. Among subnational locations with available frontier inputs, positive gaps were observed in 374 of 389 locations across 14 countries.

**Conclusion:**

Drowning in the population < 20 years still caused substantial premature mortality in 2023. Age patterns, rate-based and number-based rankings, subnational variation, and frontier gaps showed that national averages alone were insufficient to identify all priority populations for drowning prevention.

## 1 Introduction

Drowning remains one of the most preventable causes of fatal injury in childhood and adolescence (1). Its impact is obvious in deaths that occur early in life, so even a relatively small number of events can translate into a large loss of life-years (2, 3). Global prevention guidance has repeatedly emphasized supervision, safe childcare, barriers around hazardous water, water-safety skills, timely rescue, and emergency care (4, 5). And yet, the populations most likely to benefit from these measures are not distributed evenly (6). Children’s exposure to water hazards depends on age, household environment, local geography, flooding, recreation, transport, and the reach of community prevention systems (5, 7–9). For this reason, estimates for the population younger than 20 years are most informative if they could show how preventable mortality and life-years lost may vary by age and location setting, rather than only describing global or national totals.

Current evidence still leaves several prevention-relevant issues unresolved (10–12). Previous comparative studies of drowning, including the Global Burden of Disease (GBD) studies, have mainly focused on all-age burden at global, regional, or national levels. Such broad summaries may obscure age-specific patterns in children and adolescents, since water exposure and feasible prevention measures change across childhood and adolescence (13–15). The second knowledge gap concerns the location setting of the drowning burden estimates. National estimates can identify high-burden countries, but they cannot show whether the burden is the highest in geographic scale involving provinces, states, or districts. Existing subnational evidence suggests that such within-country differences can be substantial (16). Thirdly, comparisons by sociodemographic development level can show whether drowning burden differs across development contexts. However, they do not show whether a country or subnational location has a higher disability-adjusted life-year (DALY) rate than other locations at a similar sociodemographic development level. Frontier analysis addresses this question by comparing the observed DALY rates with benchmark rates at comparable development levels. Nevertheless, to the best of our knowledge, it remains unclear which age groups and subnational locations have the highest rates, which countries or subnational locations account for the largest numbers of deaths and DALYs, and where rates remain high relative to other locations at a similar level of sociodemographic development.

The primary objective of this study was to identify the age groups, countries, and subnational locations that still accounted for substantial under-20 drowning deaths and DALYs during 1990–2023. Using the latest GBD estimates updated to 2023, we examined absolute numbers and rates of deaths and DALYs among population < 20 years across 204 countries and territories. We then ranked countries separately by DALY rate and by the number of DALYs to distinguish countries with the highest average risk from those suffering the greatest absolute health loss. For countries with available subnational estimates, we examined within-country variation and compared local DALY rates with their corresponding national averages. Finally, we summarized sociodemographic inequality and frontier gaps at national and subnational levels to assess how drowning burden varied with sociodemographic development.

## 2 Methods

### 2.1 Study design and data source

We conducted a descriptive analysis of the aggregated estimates for drowning burden. Estimates were obtained from the Institute for Health Metrics and Evaluation (IHME) database.^1^ The GBD 2023 study implements standardized cause-of-death and non-fatal estimation procedures to generate comparable estimates by cause, location, year, age, and sex (3). For drowning, we extracted deaths, DALYs, years of life lost (YLLs), years lived with disability (YLDs). For each measure, we retained counts, rates per 100,000 population, and 95% uncertainty intervals (UIs). Deaths and DALYs were used to describe the absolute level of drowning burden, whereas age-specific death and DALY rates were used for comparisons across ages and locations. Following previous studies (17, 18), we used both crude and age-specific rates to summarize and compare differences across countries and locations among population < 20 years. DALY rate was also used for national rankings, subnational comparisons, comparisons with national averages, and frontier analyses. YLLs and YLDs were then used to describe the premature mortality and non-fatal components of DALYs.

### 2.2 Geographic levels

Geographic analyses included the global estimate, five socio-demographic index (SDI) groups, 21 regions, and 204 countries and territories. The five SDI groups were low, low-middle, middle, high-middle, and high SDI. Subnational analyses were limited to countries with available local estimates for drowning among population < 20 years. In 2023, 507 subnational locations in 17 countries were included in the within-country analyses of DALY rates. These subnational estimates were used to assess geographic variation and to compare local DALY rates with their corresponding national estimates.

### 2.3 Study population and age groups

The target population was people younger than 20 years. Main analyses used estimates for both sexes combined, while sex-specific estimates were used to describe male–female differences. Age-specific analyses followed the age categories available in the extracted estimates. We examined population younger than 1 year, younger than 5 years, 5–9 years, 10–14 years, and 15–19 years. To describe early-childhood patterns in more detail, we also examined newborn children aged 6–11 months and those aged 12–23 months and 2–4 years. Although these early-childhood categories overlap with broader age groups, we retained the analyses for these categories to show the drowning risk in these specific age subgroups.

### 2.4 Temporal, national, and subnational comparisons

Based on global total number and rate of deaths and DALYs during 1990–2023, long-term trends were assessed by comparing estimates for 2023 with those for 1990. Percentage change was calculated as the difference between the 2023 and 1990 estimates divided by the 1990 estimate and expressed as a percentage. Next, countries and territories were ranked separately by DALY rate and by the number of DALYs in 2023, to distinguish high-rate settings from countries contributing the largest absolute health loss. For each country with subnational data, we identified the subnational locations with the highest and lowest DALY rates, calculated the absolute rate difference, and calculated the ratio of maximum-to-minimum rate to describe within-country variation. We also compared each subnational DALY rate with the corresponding national DALY rate for the same year, age group, sex, cause, and measure. The number of subnational locations with DALY rates above the corresponding national estimate was then counted.

### 2.5 Inequality analysis and frontier gaps based on SDI

We adopted the SDI metric to assess how drowning DALY rates varied with sociodemographic development. We used SDI to assess inequality in drowning DALY rates across levels of sociodemographic development. Countries and territories were ordered from the lowest to the highest SDI and assigned a relative rank based on the midpoint of the cumulative population distribution, whereas the slope index of inequality (SII) was estimated as the slope coefficient from a population-weighted linear regression of DALY rates on this relative rank and was used to measure absolute inequality. The concentration index (CI) was used to measure relative inequality and was calculated based on the relationship between the cumulative proportion of drowning DALYs and the cumulative population share, with countries ranked from lowest to highest SDI (19, 20). Signed CI values were reported rather than absolute values. Negative SII and CI values indicated that drowning burden was greater among populations at the lower end of the SDI distribution (20). CI Frontier analysis was conducted using the extracted DALY-rate estimates and corresponding SDI values, following previously described frontier approaches (21, 22). This analysis estimated the lowest observed DALY rate at each SDI level as the benchmark. The frontier gap was defined as the observed DALY rate minus this benchmark rate, with positive values indicating rates above the benchmark. At the country level, the 2023 frontier analysis included all 204 countries and territories in the national analysis. At the subnational level, the frontier analysis was restricted to locations with available SDI values and DALY-rate estimates.

### 2.6 Statistical analysis

Descriptive tabulation and comparison of the extracted estimates were conducted, along with their available 95% UIs. Following previous studies (23–25), we then used point estimates to derive percentage changes, rankings, maximum-to-minimum rate ratios, national rates, and frontier gaps. Similar to previous studies, uncertainty intervals were not propagated through these derived quantities. We did not perform inferential testing, since the study was designed to summarize population estimates rather than to test a statistical hypothesis. The analysis used only aggregated estimates with no individual-level records, so ethics review and informed consent were not applicable.

## 3 Results

### 3.1 Global burden and temporal trends

**Figure 1:**
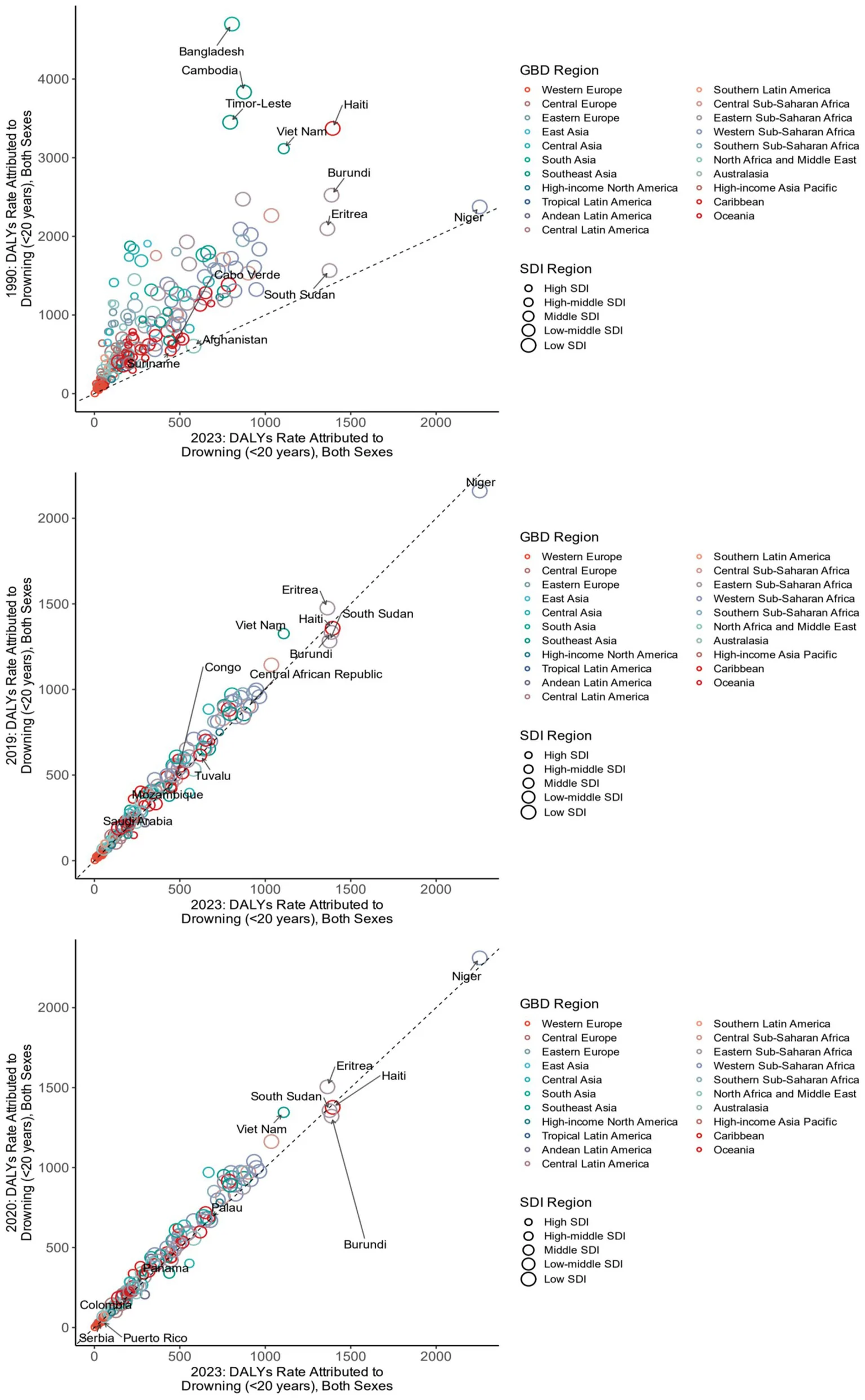
Changes in under-20 drowning DALY rates from 1990, 2019, and 2020 to 2023 across global, regional, and national locations.DALY, disability-adjusted life-year; SDI, Socio-demographic Index. Each panel places the 2023 drowning DALY rate on the *x*-axis and the corresponding rate in 1990, 2019, or 2020 on the *y*-axis. Points denote individual locations. Color and point size follow the regional and SDI groupings shown in the source figure. The diagonal reference line marks equal rates in the paired years. Estimates are for both sexes combined, and rates are per 100,000 population.

As shown by Figure 1, by 2023, drowning mortality among population < 20 years had dropped since 1990, but drowning still caused a large number of annual deaths and substantial DALY loss, mainly through premature mortality. Globally, an estimated 127,762 deaths (95% UI 95,352 to 169,781) and 10.48 million DALYs (7.79 to 13.96 million) were attributable to drowning in this age group. The death rate was 4.79 per 100,000 population (3.57 to 6.36), and the DALY rate was 392.71 per 100,000 population (292.10 to 523.10) (Supplementary Figure S1). From 1990 to 2023, the death rate decreased by 68.4%, and the DALY rate decreased by 68.8%. In 2023, YLLs accounted for 99.86% of all drowning DALYs, whereas YLDs contributed only 14,923 DALYs, indicating that the health loss in this age group remained dominated by fatal outcomes.

### 3.2 Age-specific drowning burden

The burden in 2023 was the highest in early childhood. The global DALY rate was 654.10 per 100,000 population (95% UI 474.35 to 895.43) among children younger than 5 years, compared with 372.41 per 100,000 (279.52 to 489.93) at ages 5–9 years, 322.00 per 100,000 (249.75 to 411.79) at ages 10–14 years, and 228.99 per 100,000 (169.43 to 318.11) at ages 15–19 years. Within the younger-than-5-years group, the highest rate was observed at ages 12–23 months (877.21 per 100,000 [638.77 to 1163.89]), and the levels of DALY rate were also prominent at ages 6–11 months (704.35 [447.38 to 1110.94]) and 2–4 years (601.10 [447.22 to 792.61]). Male children and adolescents had a higher DALY rate than female children and adolescents in 2023 (510.07 vs. 268.33 per 100,000 population). Detailed estimates are summarized in Supplementary Table S1.

### 3.3 National differences by rate and absolute number

Country rankings in 2023 differed depending on whether drowning burden was measured as a population rate or as an absolute number. The highest DALY rates among population < 20 years were in Niger (2255.15 per 100,000 population [95% UI 1118.72 to 3655.78]), Haiti (1394.66 [807.01 to 2024.65]), Burundi (1387.89 [884.09 to 2106.48]), South Sudan (1376.05 [836.93 to 2076.97]), and Eritrea (1363.60 [839.78 to 1996.16]). Vietnam, Angola, and Chad also had DALY rates above 900 per 100,000 (Figure 1). By contrast, the largest absolute DALY burdens were in India (1.65 million [1.10 to 2.30 million]), China (1.03 million [0.85 to 1.26 million]), Pakistan (575,481 [363,058 to 924,386]), Nigeria (573,442 [358,462 to 884,980]), and Bangladesh (541,149 [351,145 to 723,620]). Detailed estimates are summarized in Supplementary Tables S2 and S3.

### 3.4 Subnational variation within countries

Subnational estimates for 2023 were available for 507 locations in 17 countries (Supplementary Figure S1). Across all subnational locations, the highest DALY rates were observed in Papua in Indonesia (1506.13 per 100,000 population), Apayao in the Philippines (1329.63), South West Ethiopia (1176.34), Catanduanes in the Philippines (1098.25), Ebonyi in Nigeria (1037.84), and Eastern Samar in the Philippines (1035.33). The locations with the largest absolute numbers of DALYs were different, led by Bihar in India (268,569 DALYs), Uttar Pradesh in India (263,206), Madhya Pradesh in India (174,065), Sindh in Pakistan (131,385), and West Bengal in India (122,903).

**Figure 2:**
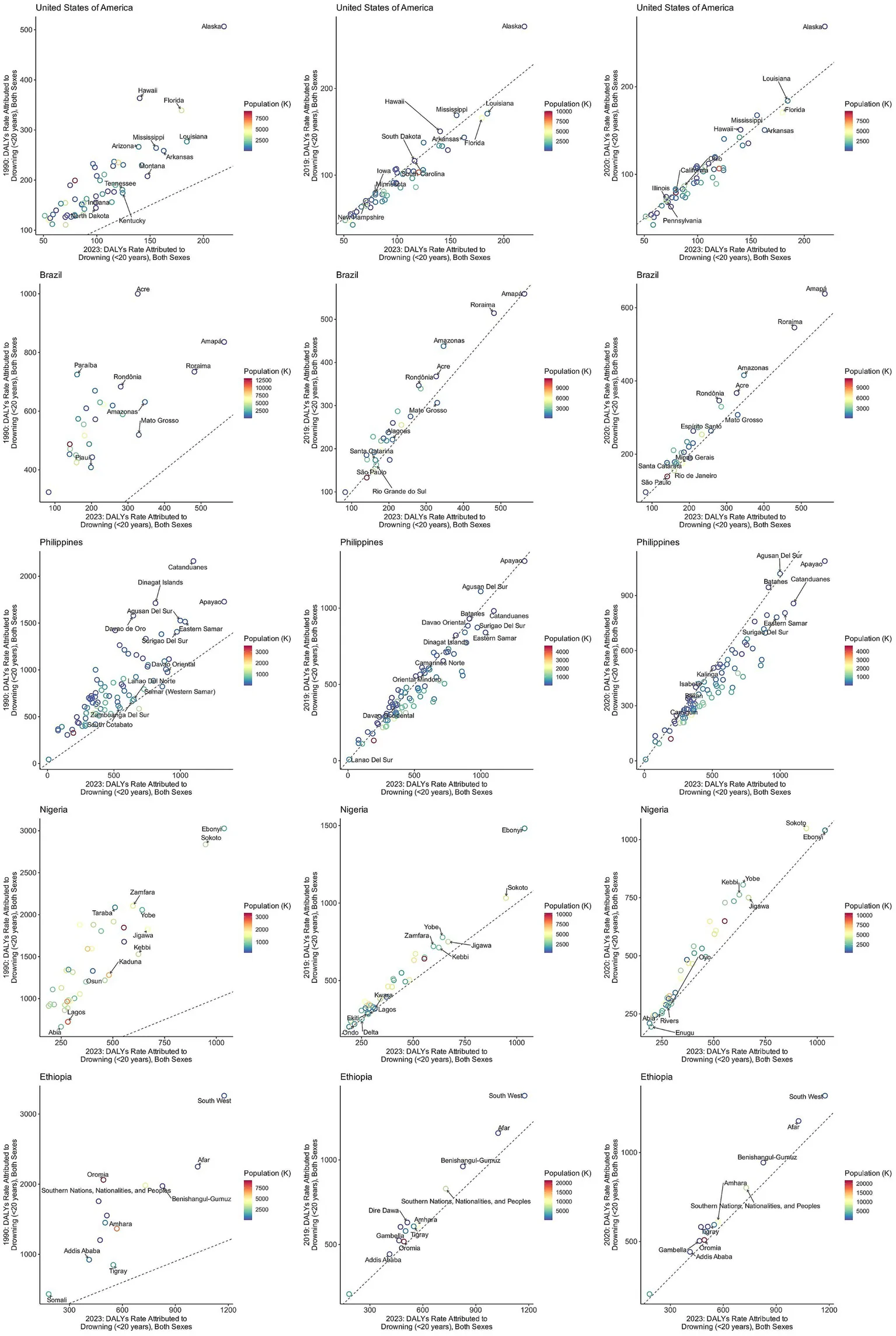
Selected examples of province-level variation in under-20 drowning DALY rates, 1990–2023.DALY, disability-adjusted life-year; SDI, Socio-demographic Index. Five countries with subnational estimates are displayed to show how local rates varied within national borders in 2023: United States of America, Brazil, Philippines, Nigeria, and Ethiopia. The three columns compare subnational rates in 1990, 2019, and 2020 with rates in 2023. Each point is a subnational location, and the diagonal line indicates equal values in the 2 years being compared. Rates are per 100,000 population and are shown for both sexes combined.

Large within-country contrasts were also observed (Figure 2). In the Philippines, the DALY rate ranged from 1329.63 per 100,000 in Apayao to 8.36 in Lanao Del Sur, a 159.1-fold difference. In Indonesia, the rate ranged from 1506.13 in Papua to 103.94 in Yogyakarta, a 14.5-fold difference. The corresponding ratios were 10.5 in Iran, 7.6 in Kenya, and 6.8 in Brazil. The within-country range was narrower in some settings, including South Africa (Eastern Cape 380.56 vs. Gauteng 217.56; 1.7-fold) and the United Kingdom (Scotland 27.70 vs. Wales 16.26; 1.7-fold).

National rates indeed concealed locations with above-average burden. For example, subnational DALY rates were higher than their corresponding national rates in 41 of 82 locations in the Philippines, 22 of 51 in the United States, 19 of 47 in Kenya, 18 of 32 in Mexico, 15 of 27 in Brazil, 15 of 31 in Iran, and 14 of 34 in Indonesia (Table 1).

**Table 1:** Country-specific spread of subnational drowning DALY rates among population younger than 20 years, 2023.

Country	Subnational locations, n	National DALY rate, 2023	Highest subnational DALY rate	Lowest subnational DALY rate	Absolute difference	Max/min ratio	Above national, n (%)
Philippines	82	438.10 (328.18 to 566.86)	Philippines: Apayao: 1,329.63 (763.09 to 2,003.53)	Philippines: Lanao Del Sur: 8.36 (4.98 to 14.59)	1,321.27	159.08	41/82 (50.0%)
Indonesia	34	384.14 (229.95 to 605.23)	Indonesia: Papua: 1,506.13 (814.14 to 2,345.53)	Indonesia: Yogyakarta: 103.94 (63.15 to 165.34)	1,402.19	14.49	14/34 (41.2%)
Iran (Islamic Republic of)	31	115.58 (82.14 to 155.60)	Iran (Islamic Republic of): Khuzestan: 277.00 (171.79 to 423.15)	Iran (Islamic Republic of): Tehran: 26.50 (14.45 to 55.36)	250.49	10.45	15/31 (48.4%)
Kenya	47	293.55 (189.10 to 434.01)	Kenya: Wajir: 613.09 (317.45 to 966.98)	Kenya: Nyandarua: 80.29 (44.60 to 127.84)	532.80	7.64	19/47 (40.4%)
Brazil	27	190.69 (172.41 to 209.64)	Brazil: Amapá: 563.15 (460.70 to 677.51)	Brazil: Distrito Federal: 82.81 (68.08 to 99.08)	480.34	6.80	15/27 (55.6%)
Ethiopia	13	554.02 (363.12 to 811.08)	Ethiopia: South West: 1,176.34 (657.51 to 1,929.33)	Ethiopia: Somali: 182.13 (102.24 to 351.21)	994.21	6.46	5/13 (38.5%)
India	30	332.60 (221.64 to 463.56)	India: Madhya Pradesh: 497.74 (298.21 to 784.00)	India: Delhi: 87.42 (44.80 to 170.83)	410.32	5.69	12/30 (40.0%)
Nigeria	37	426.37 (266.52 to 658.00)	Nigeria: Ebonyi: 1,037.84 (609.24 to 1,664.50)	Nigeria: Edo: 189.65 (119.04 to 302.21)	848.19	5.47	13/37 (35.1%)
Mexico	32	116.70 (103.16 to 133.30)	Mexico: Tabasco: 215.92 (178.58 to 259.63)	Mexico: Mexico City: 41.09 (33.98 to 50.27)	174.83	5.25	18/32 (56.2%)
United States of America	51	100.98 (88.07 to 114.78)	United States of America: Alaska: 219.22 (186.44 to 251.95)	United States of America: New Jersey: 51.26 (42.18 to 61.50)	167.96	4.28	22/51 (43.1%)
Japan	47	44.66 (37.95 to 51.44)	Japan: Fukui: 91.74 (79.36 to 108.66)	Japan: Saitama: 23.00 (18.37 to 28.52)	68.74	3.99	32/47 (68.1%)
Pakistan	7	481.35 (303.67 to 773.18)	Pakistan: Azad Jammu & Kashmir: 504.96 (314.21 to 760.45)	Pakistan: Punjab: 165.60 (97.01 to 268.40)	339.36	3.05	1/7 (14.3%)
Italy	21	20.68 (17.97 to 23.20)	Italy: Provincia autonoma di Bolzano: 35.97 (29.57 to 43.37)	Italy: Calabria: 12.25 (9.57 to 16.13)	23.71	2.94	10/21 (47.6%)
Norway	11	44.60 (36.12 to 53.42)	Norway: Troms og Finnmark: 78.35 (62.44 to 94.45)	Norway: Oslo: 29.17 (23.44 to 35.32)	49.18	2.69	7/11 (63.6%)
Poland	16	35.38 (29.69 to 42.13)	Poland: Warmińsko-Mazurskie: 53.75 (41.26 to 67.48)	Poland: Śląskie: 26.18 (20.84 to 32.35)	27.56	2.05	9/16 (56.2%)
South Africa	9	276.31 (217.77 to 333.45)	South Africa: Eastern Cape: 380.56 (247.60 to 515.53)	South Africa: Gauteng: 217.56 (166.88 to 278.97)	163.00	1.75	5/9 (55.6%)
United Kingdom	12	21.05 (16.85 to 24.83)	United Kingdom: Scotland: 27.70 (22.17 to 34.92)	United Kingdom: Wales: 16.26 (12.33 to 20.12)	11.44	1.70	4/12 (33.3%)

DALY, disability-adjusted life-year, UI, uncertainty interval, SDI=Socio-demographic Index. The table summarizes 507 subnational locations. Rates and differences are per 100,000 population. Uncertainty intervals are presented for source estimates. The derived contrasts, ratios, and counts above the national value were calculated from point estimates. Above-national counts describe locations rather than population shares.

### 3.5 Sociodemographic inequality and frontier gaps

**Figure 3:**
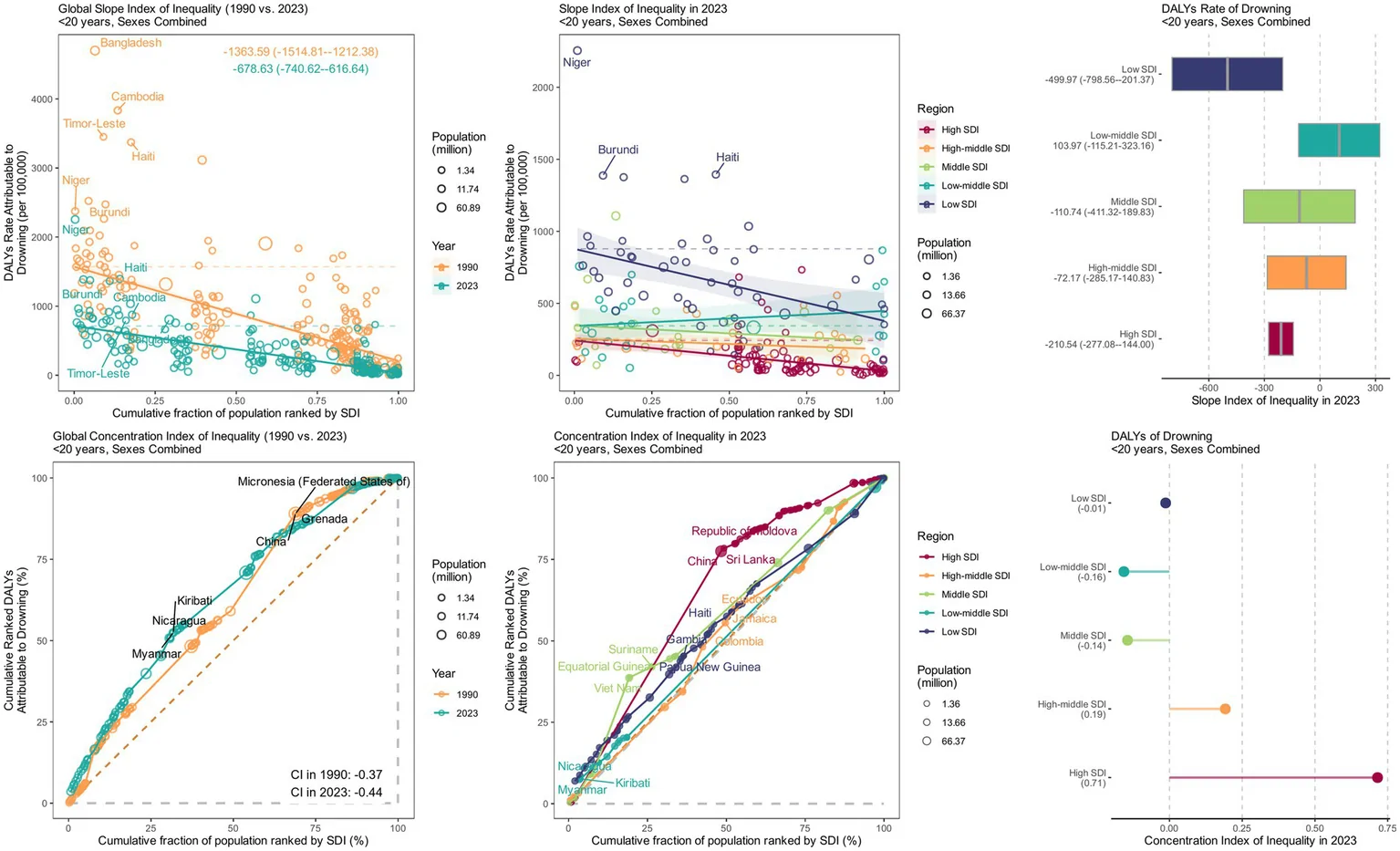
SDI-related absolute and relative inequality in drowning DALYs among population younger than 20 years, 1990 and 2023.DALY, Disability-adjusted life-year; SDI, Socio-demographic Index; SII, Slope index of inequality; CI, Concentration index. Countries and territories were ranked from the lowest to the highest SDI. CI values retain their signs and are not absolute values. Negative CI values indicate that drowning DALYs are disproportionately higher among lower-SDI populations, whereas positive values indicate that they are disproportionately higher among higher-SDI populations. Negative SII values indicate higher DALY rates at the lower end of the SDI distribution. Upper panels present absolute inequality using the SII, and lower panels present relative inequality using the CI. The left panels compare the global pattern in 1990 and 2023. The middle panels plot 2023 country and territory estimates by SDI group, with marker size proportional to population. The right panels summarize SII for DALY rates and CI for DALYs across SDI groups in 2023. Rates are per 100,000 population and all estimates are for both sexes combined.

SDI-related inequality persisted in 2023. For DALY rates among population <20 years, the global slope index of inequality changed from −1363.59 in 1990 (95% UI -1514.81 to −1212.38) to −678.63 in 2023 (−740.62 to −616.64). The negative SII values indicated higher drowning DALY rates at the lower end of the SDI distribution, while the movement toward zero indicated a reduction in absolute inequality. The global concentration index changed from −0.37 in 1990 to −0.44 in 2023. The negative CI values indicated that drowning DALYs were greater among lower-SDI populations, while the increasing absolute magnitude suggested that relative inequality widened over time (Figure 3).

**Figure 4:**
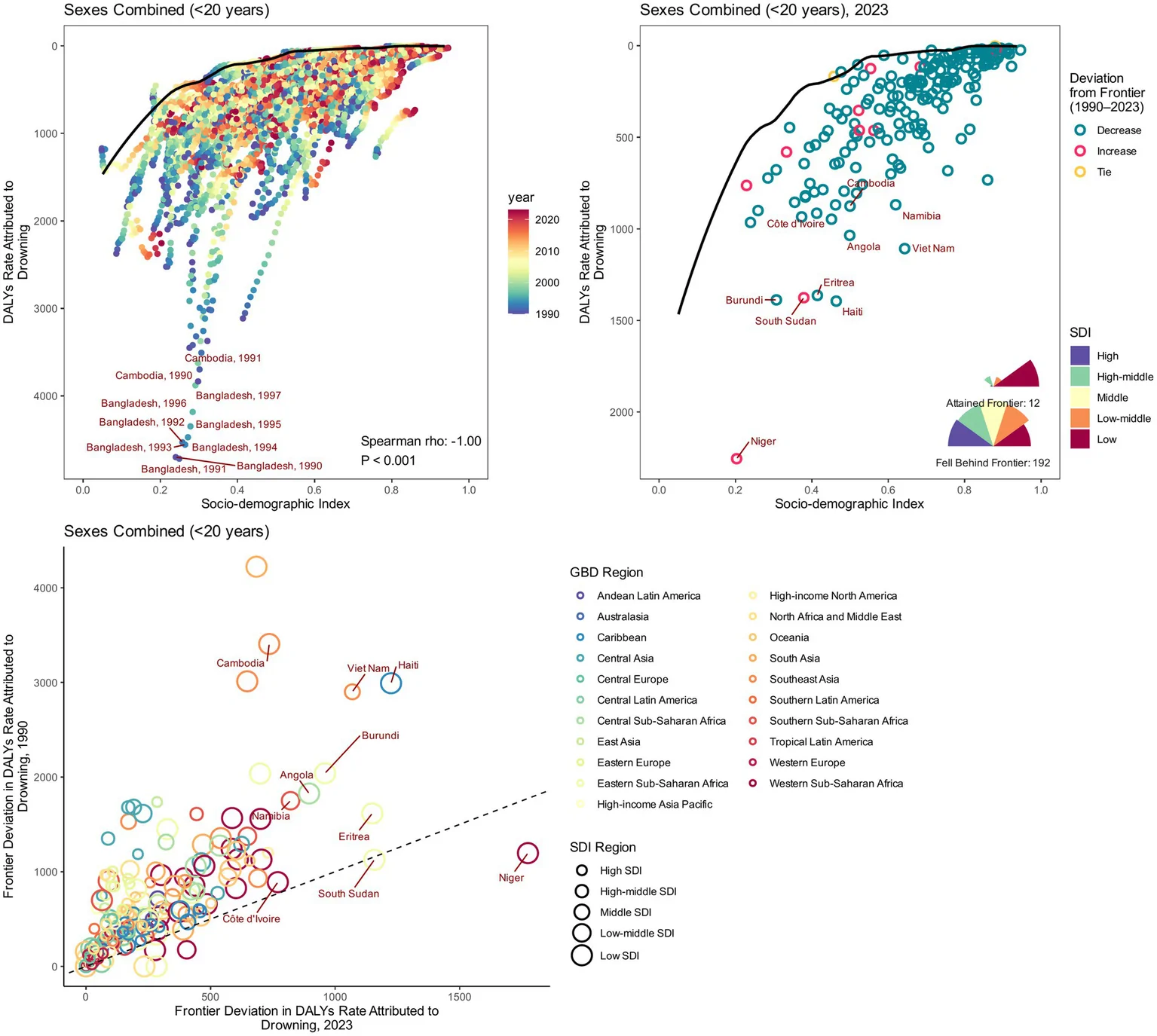
Country-level frontier comparison for under-20 drowning DALY rates, 1990–2023.DALY, disability-adjusted life-year; SDI, Socio-demographic Index. The upper-left panel relates SDI to drowning DALY rates for countries and territories from 1990 to 2023, with the black curve representing the frontier rate. The upper-right panel shows 2023 country and territory estimates relative to that frontier. The lower-left panel contrasts each location’s distance from the frontier in 1990 and 2023. Points are grouped by region or by change in frontier distance, and point size reflects SDI group. Locations above the curve had higher observed rates than the frontier value at similar SDI. Of the 204 locations, 12 had lower 95% UI bounds at or below the frontier value and were labelled as having attained the frontier in the figure. In the other 192 locations, the lower UI bound remained above the frontier. However, to remain consistent with previous publication (24), the frontier gaps reported in the primary findings were calculated using the GBD point estimates. Rates and frontier deviations are per 100,000 population and are shown for both sexes combined.

Frontier analysis showed that most countries still had DALY rates above the benchmark given their corresponding SDI level in 2023. Among 204 countries and territories, 199 had positive frontier gaps. The largest gaps were observed in Niger (1773.86 per 100,000 population), Haiti (1224.36), South Sudan (1157.39), Eritrea (1148.93), Vietnam (1069.11), Burundi (959.58), and Angola (895.55) (Figure 4). At the subnational level, the frontier analysis included 389 locations in 14 countries with available SDI values and DALY-rate estimates, and we found substantial variation in frontier gaps across subnational locations (Supplementary Figure S2). In 2023, positive gaps were identified in 374 of 389 subnational locations with available frontier estimates. The largest gaps were observed in Papua in Indonesia (1327.09 per 100,000 population), South West Ethiopia (989.99), Ebonyi in Nigeria (855.07), Afar in Ethiopia (825.72), Benishangul-Gumuz in Ethiopia (640.54), and Sokoto in Nigeria (552.42).

## 4 Discussion

The study found that, despite long-term declines, under-20 drowning burden in 2023 remained prominent in early childhood and in countries or subnational locations with high DALY rates. Specifically, even though global death and DALY rates have declined by 68.4% and 68.8%, respectively, from 1990 to 2023, drowning still accounted for an estimated 127,762 deaths and 10.48 million DALYs in 2023. The burden was almost entirely fatal, with YLLs accounting for 99.86% of DALYs. The highest rates occurred in early childhood, and the countries with the highest DALY rates were not the same as those contributing the largest numbers of DALYs. Subnational results further showed that locations with DALY rates above their corresponding national averages were mostly located in middle-to-high SDI countries, indicating that within-country drowning burden was not confined to low-SDI settings. Prevention resources should not be allocated merely on the basis of national estimates, but should be guided by local assessment of drowning burden and risks, especially for early-childhood age groups and high-burden areas.

### 4.1 Added value of the current study to the existing evidence

Recent comparative studies have shown declines in all-age drowning deaths and all-age DALY rates over time, with uneven progress across various regions and countries (10–12). We extended this evidence by focusing on drowning burden among children and adolescents. Although country-level estimates may help identify national priorities, they cannot show local prevention priorities. Specifically, national estimates could not demonstrate which local populations continue to experience high rates after national improvement has occurred. Combining national rankings with available subnational estimates allowed us to assess drowning burden with greater relevance to local prevention planning and policy implementation (4, 26).

### 4.2 The age window for drowning prevention

The age-specific findings further support separate prevention planning for early childhood and for older children and adolescents. The highest DALY rate occurred at ages 12–23 months, and rates were also high at ages 6–11 months and 2–4 years. This pattern is developmentally and behaviorally plausible. During this period, children gain mobility before they have the judgment, coordination, or strength to avoid water hazards or escape from such hazards (27). In early childhood, exposure often comes from water hazards in ordinary daily environments rather than from intentional swimming or other recreational activities. Ponds, irrigation channels, wells, buckets, tanks, bathtubs, and uncovered water-storage containers can become hazardous when children have unsupervised access (13, 26, 28–31). In later childhood and adolescence period, exposure is more likely to involve various recreational activities such as playing in the water. Travel near water, work or chores near water, floodwater exposure, and unsupervised swimming may also become more common (4). Prevention strategies to reduce under-20 drowning burden should therefore be tailored to detailed age-specific risks, rather than designed and implemented uniformly across this age range (13).

### 4.3 Nations that need to set priorities for injury prevention

The national findings show why rate and absolute number of burden estimates should not be treated as interchangeable measures. Countries such as Niger, Haiti, Burundi, South Sudan, and Eritrea had the highest DALY rates in 2023, indicating a high level of burden relative to the size of the under-20 population. The largest numbers of DALYs, however, were the highest in India, China, Pakistan, Nigeria, and Bangladesh. Higher DALY rates identified countries where the average risk among children and adolescents was higher (32). In comparison, a larger total number of DALYs may help identify countries where prevention could avert greater total health loss. The policy response should therefore differ. For countries with high DALY rates, prevention planning should target the main sources of water exposure and address gaps in the supervision of hazardous water bodies, rescue capacity, emergency care, and injury surveillance (33). For countries with the largest absolute number of DALYs, drowning prevention programs should be designed and implemented to benefit larger numbers of children and households (34), even when the corresponding national DALY rates may appear lower.

### 4.4 Subnational variation beyond national averages

The subnational findings highlighted priority locations that were not evident from national rankings alone. Some of the highest local DALY rates were observed in locations such as Papua in Indonesia, Apayao in the Philippines, and South West Ethiopia, whereas the largest absolute subnational burden occurred in more populous locations such as Bihar and Uttar Pradesh in India and Sindh in Pakistan. These comparative findings show that subnational locations with the highest average risk are not necessarily those with the greatest absolute number of deaths or DALYs. Large within-country maximum-to-minimum rate ratio also suggest that children living in different locations within the same country may face substantially different drowning risks. Similarly, the large number of subnational locations with DALY rates above the corresponding national estimate provides further evidence that national averages may obscure substantial local variation. These findings suggest that national estimates should be used as a starting point. Subnational estimates can then guide local resource allocation and help target intensified prevention efforts.

### 4.5 Inequality and frontier gaps

In the global SDI-related inequality analysis, the SII moved toward zero between 1990 and 2023, indicating a reduction in absolute SDI-related inequality. In comparison, the CI changed from −0.37 in 1990 to −0.44 in 2023. The negative CI showed that the drowning DALY burden was skewed toward lower-SDI populations, and its increasing absolute magnitude indicated persistent relative inequality. The frontier analysis further complements this inequality assessment by identifying locations that remain far from the benchmark frontier expected at comparable SDI levels, consistent with previous GBD frontier studies (22, 26, 35, 36). Specifically, for countries with comparable SDI levels, larger frontier gaps may indicate greater unmet potential to reduce drowning burden compared with the benchmark frontier estimated at the same SDI level. For instance, we observed larger gaps in countries such as Niger and Haiti, and in subnational locations such as Papua in Indonesia and South West Ethiopia. Such locations remain farther from the benchmark frontier and may warrant targeted assessment of drowning risk factors, local prevention capacity, injury surveillance, and data quality (37).

### 4.6 Public health implications

The implications are therefore not the same for every setting. In early childhood, the priority is to reduce unsupervised access to nearby water through supervision, safe childcare, household hazard reduction, and barriers around wells, tanks, ponds, canals, or other water sources (5, 13, 38). In countries or local areas with high DALY rates, prevention planning should begin with a review of local water exposure, supervision practices, flood risk, rescue capacity, and emergency care (4, 33, 39). In locations with large numbers of deaths or DALYs, the challenge is more about achieving broad coverage. This means that interventions need to reach as many children and households as possible (40), even if the average rate is not among the highest. Subnational locations with DALY rates above the corresponding national averages or those with larger frontier gaps should therefore be prioritized for local assessment to inform the design and implementation of drowning prevention strategies. Previous findings from local injury prevention programs indeed support this interpretation. For instance, in Alaska, United States, *the Kids Do not Float* program combines life-jacket loaner boards with water-safety education (41, 42). In rural Qingyuan, China, an integrated intervention program targeted townships with high drowning mortality and areas with a large number of natural or man-made water bodies (43). To enhance the efficacy of the program, the authorities and relevant departments further combined school and caregiver education, summer supervision of water areas and children, local policy action, and barriers around ponds. These examples show that prevention strategies should reflect local injury burden, available resources, and the feasibility of implementation. Large frontier gaps may also indicate settings where isolated measures are unlikely to be sufficient and where broader drowning prevention programs should be designed and implemented according to local conditions.

### 4.7 Strengths and limitations

This study has several strengths. First, it examined drowning burden among population < 20 years at both national and available subnational levels within the same estimation framework. As suggested by previous subnational burden studies (44, 45), this framework allowed us to assess whether national averages and local estimates identified the same priority populations for drowning prevention. Second, we implemented the SII and CI to quantify the sociodemographic inequality, and added frontier-gap analysis to benchmark each location against the burden expected at a comparable SDI level. This approach improves the policy interpretation of SDI-related inequality by linking inequality estimates to locations where further burden reduction may still be achievable. Third, by updating the estimates to 2023, this study provides a more recent assessment of drowning burden across early childhood, later childhood, and adolescence.

Limitations should be acknowledged. First, the estimates were derived from available mortality and health data. These estimates may be affected by sparse source data, incomplete cause-of-death registration, possible misclassification, and model adjustments (3, 46). Thus, estimation uncertainty may be larger in data-sparse settings, especially for subnational locations such as South Kalimantan in Indonesia and Balochistan in Pakistan. Yet, our standardized estimation framework may still support comparisons across locations, and local rankings of DALY rates should be interpreted together with their corresponding 95% UIs. Second, given the ecological and descriptive nature of our study, individual-level inference on drowning risks or intervention effects was not feasible (47). Nevertheless, findings from the current study may help identify population subgroups and locations where more detailed local assessment is warranted. Third, cross-country comparisons using crude DALY rates may be influenced by differences in the age structure of populations < 20 years. We therefore also reported age-specific rates, consistent with previous burden of disease studies (17, 18), to describe variation across childhood and adolescence. Finally, the source IHME data did not include local measures of water exposure, caregiver supervision, timely rescue capacity, emergency care, or implementation of water-safety programs (4). Future dedicated analyses may be tailored to examine the local drowning burden attributable to these factors.

## 5 Conclusion

In 2023, drowning among population < 20 years still caused substantial premature mortality and DALY loss, despite large rate reductions since 1990. The highest burden was observed in early childhood, while the distribution of deaths and DALYs differed substantially across countries and subnational locations. DALY-rate rankings identified locations where children and adolescents had the highest average risk. In contrast, rankings by the absolute number of DALYs identified locations where prevention could avert the greatest total health loss. Our study further revealed subnational locations with DALY rates above the corresponding national averages, suggesting that country-level estimates could obscure important local priorities. Frontier gaps also highlighted countries and subnational locations in need of in-depth local assessment, where larger reductions in drowning burden might be achievable after accounting for sociodemographic development. In brief, drowning prevention will require injury surveillance and planning that are age-specific, geographically detailed, and informed by both DALY rates and the total number of DALYs.

## Data Availability

The full dataset in this study can be obtained from http://ghdx.healthdata.org/gbd-results-tool.

## Footnotes

1
https://vizhub.healthdata.org/gbdcompare
