# Supplementary material for: Subnational inequalities and preventable gaps in drowning premature mortality and non-fatal health loss among population younger than 20 years
Source: Front Public Health. 2026 Aug 28;14:1917814. doi: 10.3389/fpubh.2026.1917814 (PMC13563505; doi:10.3389/fpubh.2026.1917814)
Supplement: Supplementary file 1 [file Supplementary_file_1.pdf]

Subnational inequalities and preventable gaps in drowning premature mortality and non-fatal health loss among population younger than 20 years

Online supplementary materials

Contents

Supplementary Table S1. 2
Supplementary Table S2. 3
Supplementary Table S3. 4
Supplementary Figure S1. 5
Supplementary Figure S2. 22

Supplementary Table S1. Global age- and sex-specific drowning DALY rates among population younger than 20 years, 2023.

Age group	DALY rates per 100,000 population (95% UI)		
	**Both sexes**	**Male**	**Female**
**<20 years**	392.71 (292.10 to 523.10)	510.07 (352.92 to 747.05)	268.33 (180.10 to 404.89)
**<5 years**	654.10 (474.35 to 895.43)	783.03 (533.57 to 1,187.28)	517.08 (337.42 to 803.86)
**<1 year**	596.23 (384.71 to 918.50)	640.85 (364.05 to 1,147.96)	548.78 (316.37 to 1,002.36)
0-6 days	1,895.99 (1,270.58 to 2,908.43)	2,404.06 (1,314.22 to 4,401.20)	1,354.48 (968.72 to 1,874.89)
7-27 days	313.58 (206.96 to 482.15)	341.30 (181.46 to 628.19)	284.09 (187.00 to 446.13)
1-5 months	448.46 (290.30 to 689.88)	486.97 (264.65 to 869.54)	407.50 (236.50 to 703.76)
6-11 months	704.35 (447.38 to 1,110.94)	738.03 (407.15 to 1,325.51)	668.53 (359.90 to 1,271.12)
12-23 months	877.21 (638.77 to 1,163.89)	1,044.80 (696.84 to 1,530.66)	699.16 (500.60 to 1,008.62)
2-4 years	601.10 (447.22 to 792.61)	744.44 (505.18 to 1,095.75)	448.80 (293.10 to 672.64)
5-9 years	372.41 (279.52 to 489.93)	483.00 (322.56 to 699.81)	254.80 (161.80 to 389.80)
10-14 years	322.00 (249.75 to 411.79)	431.79 (307.72 to 607.80)	205.60 (145.38 to 290.38)
15-19 years	228.99 (169.43 to 318.11)	349.34 (237.84 to 518.79)	102.28 (70.24 to 143.87)

Notes: Estimates represent global disability-adjusted life-year (DALY) rates per 100,000 population, with their corresponding 95% uncertainty intervals (UIs).

Supplementary Table S2. Top 10 countries with the highest drowning DALY rates among population younger than 20 years, 2023.

Rank	Country	DALY rate per 100,000 population (95% UI)
1	Niger	2,255.15 (1,118.72 to 3,655.78)
2	Haiti	1,394.66 (807.01 to 2,024.65)
3	Burundi	1,387.89 (884.09 to 2,106.48)
4	South Sudan	1,376.05 (836.93 to 2,076.97)
5	Eritrea	1,363.60 (839.78 to 1,996.16)
6	Vietnam	1,108.03 (787.23 to 1,475.16)
7	Angola	1,035.67 (655.69 to 1,589.41)
8	Chad	965.07 (560.28 to 1,525.06)
9	Côte d'Ivoire	947.44 (608.26 to 1,477.75)
10	Guinea-Bissau	934.42 (588.67 to 1,473.89)

Notes: DALY = disability-adjusted life-year; UIs=uncertainty intervals. DALY rates are calculated as values per 100,000 population.

Supplementary Table S3. Top 10 countries with the largest numbers of DALYs among population younger than 20 years, 2023.

Rank	Country	Absolute number of DALYs (95% UI)
1	India	1,651,517 (1,100,562 to 2,301,837)
2	China	1,033,301 (845,611 to 1,264,075)
3	Pakistan	575,481 (363,058 to 924,386)
4	Nigeria	573,442 (358,462 to 884,980)
5	Bangladesh	541,149 (351,145 to 723,620)
6	Democratic Republic of the Congo	380,592 (241,162 to 602,818)
7	Niger	370,719 (183,903 to 600,964)
8	Vietnam	356,888 (253,561 to 475,137)
9	Indonesia	345,487 (206,814 to 544,336)
10	Ethiopia	308,109 (201,946 to 451,072)

Notes: DALY = disability-adjusted life-year; UIs=uncertainty intervals.

Supplementary Figure S1. Subnational drowning disability-adjusted life-year (DALY) rates in 1990, 2019, and 2020, as compared with 2023, among population younger than 20 years in 17 countries.

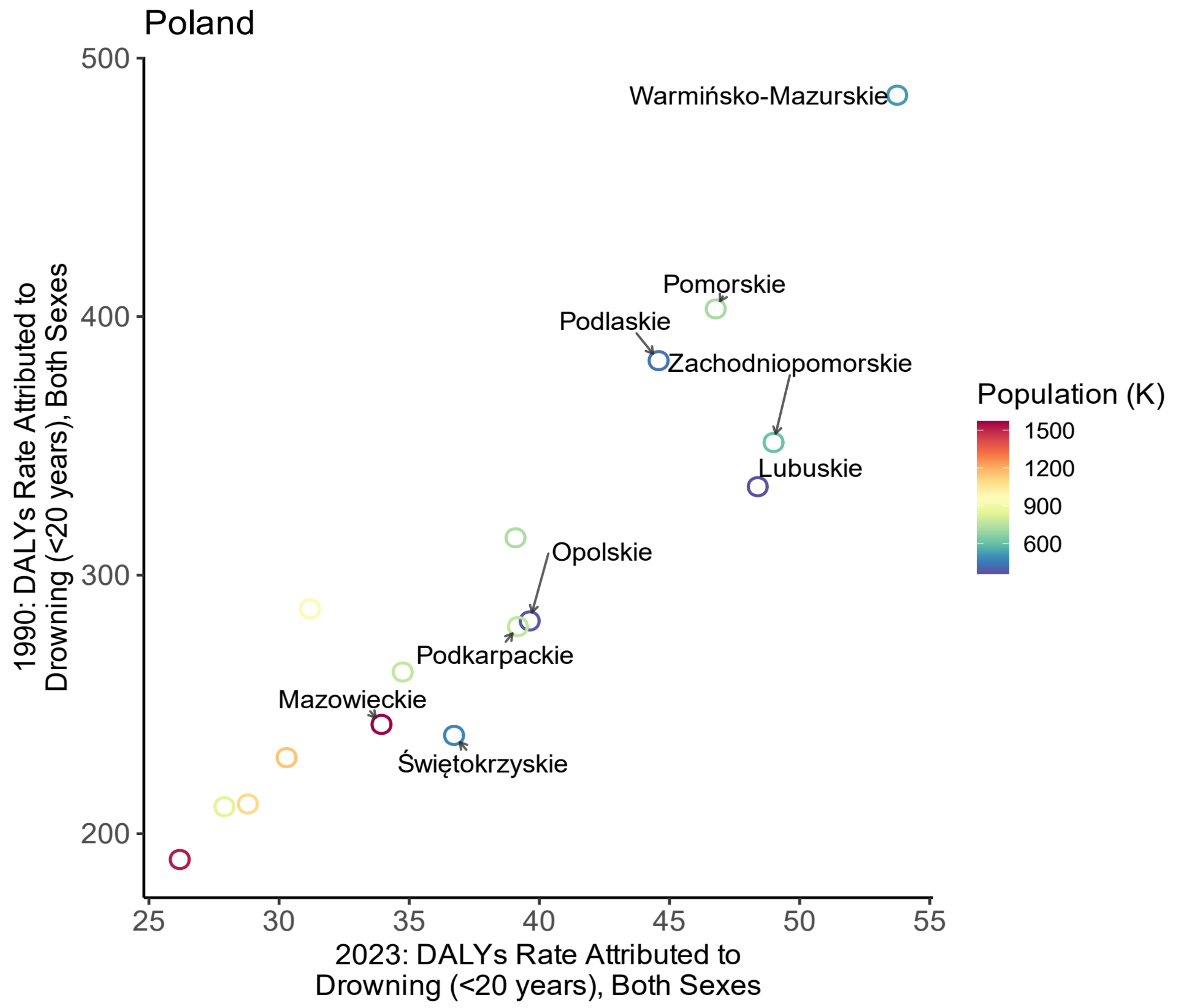

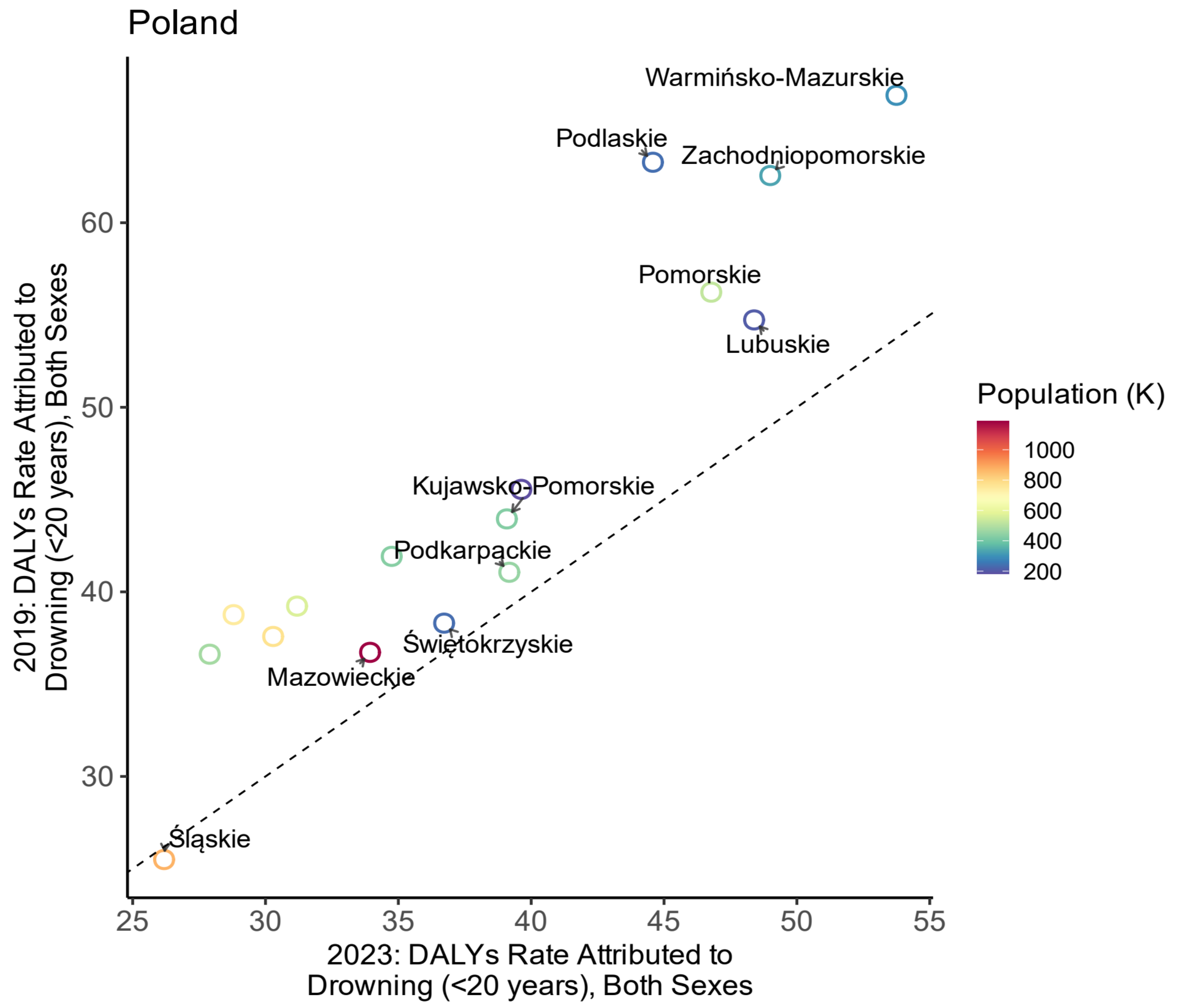

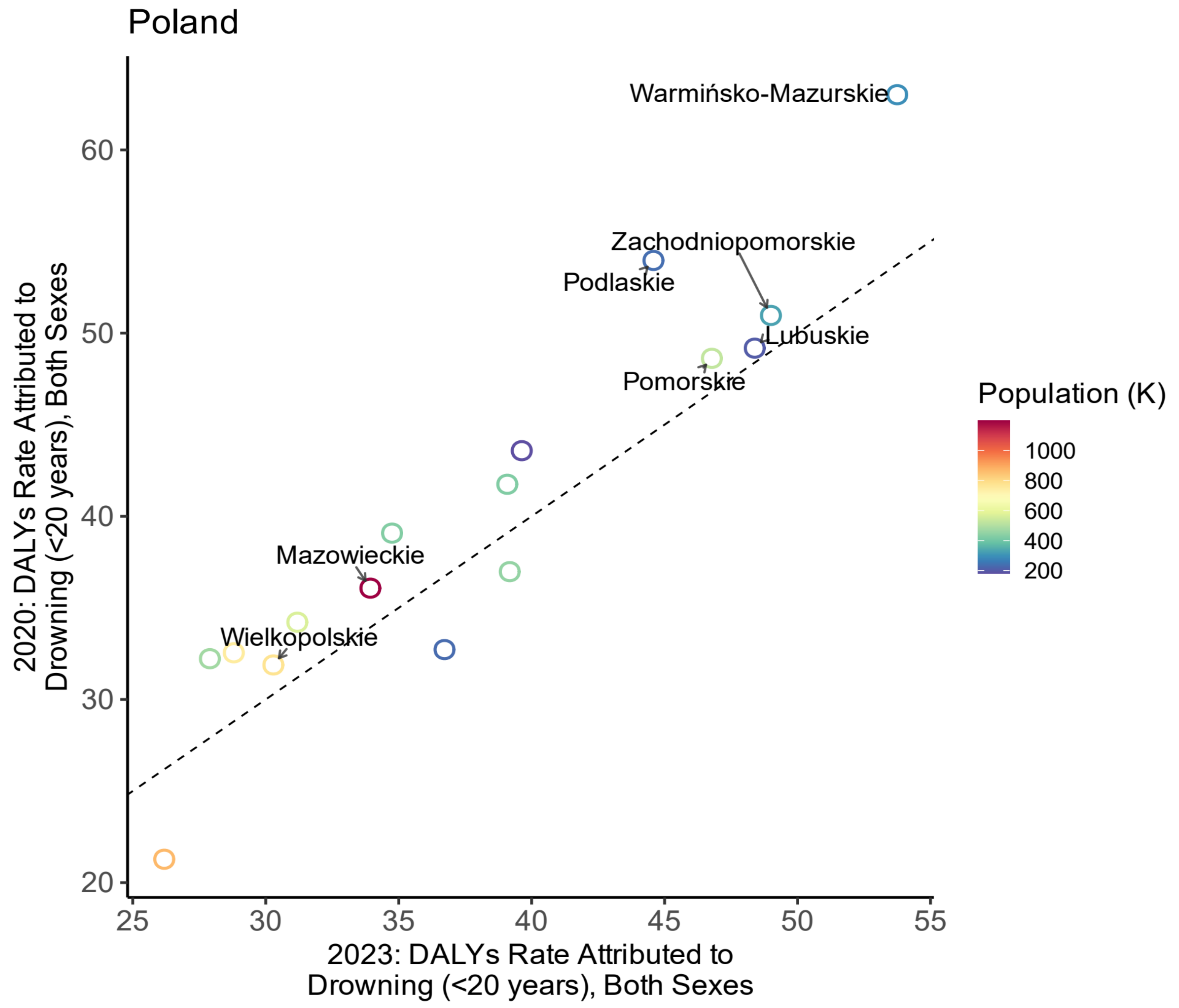

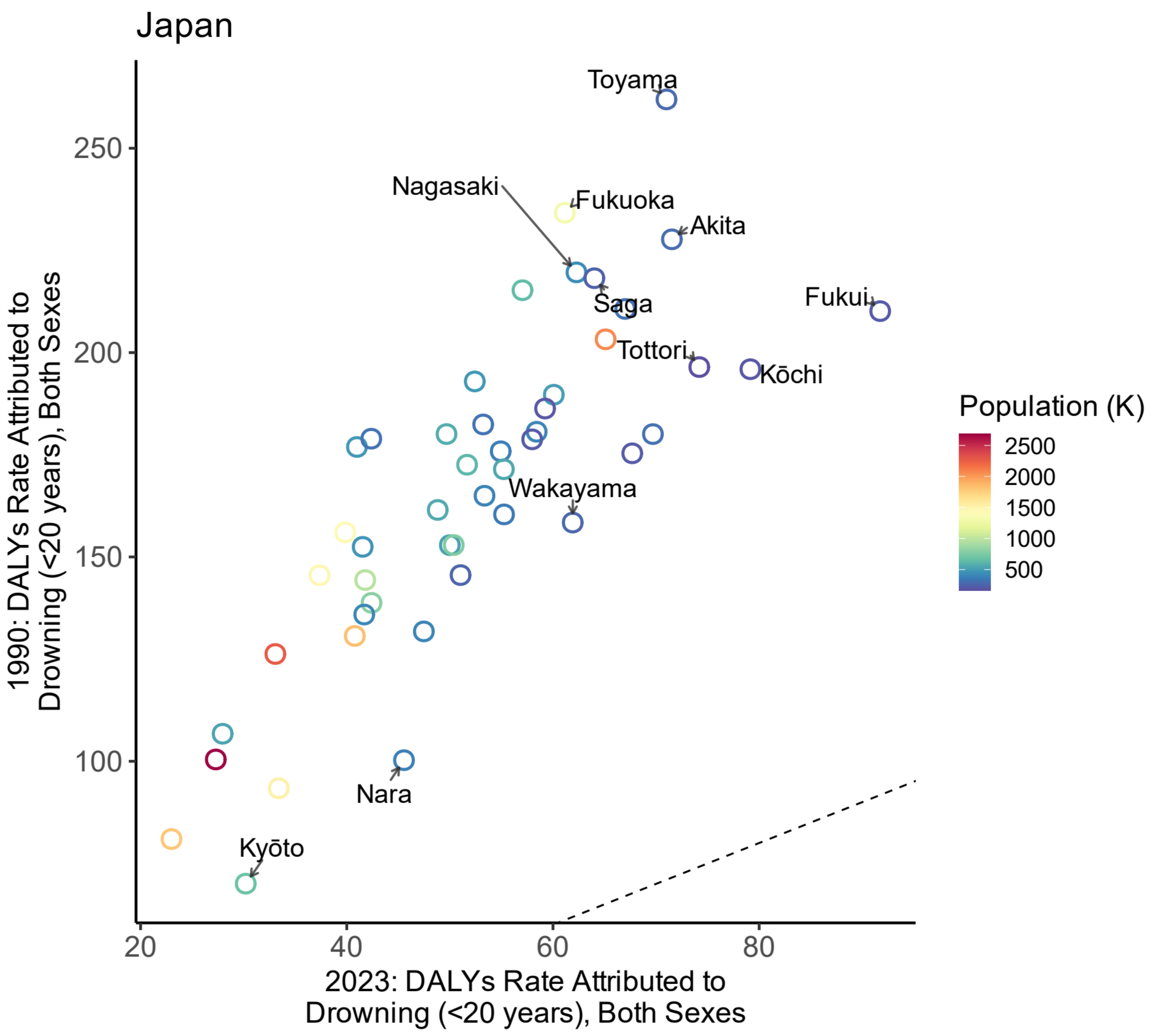
Japan
1990: DALYs Rate Attributed to Drowning (<20 years), Both Sexes
2023: DALYs Rate Attributed to Drowning (<20 years), Both Sexes
Population (K)
2500
2000
1500
1000
500
Toyama
Nagasaki
Fukuoka
Akita
Saga
Fukui
Tottori
Kōchi
Wakayama
Nara
Kyōto
250
200
150
100
20
40
60
80

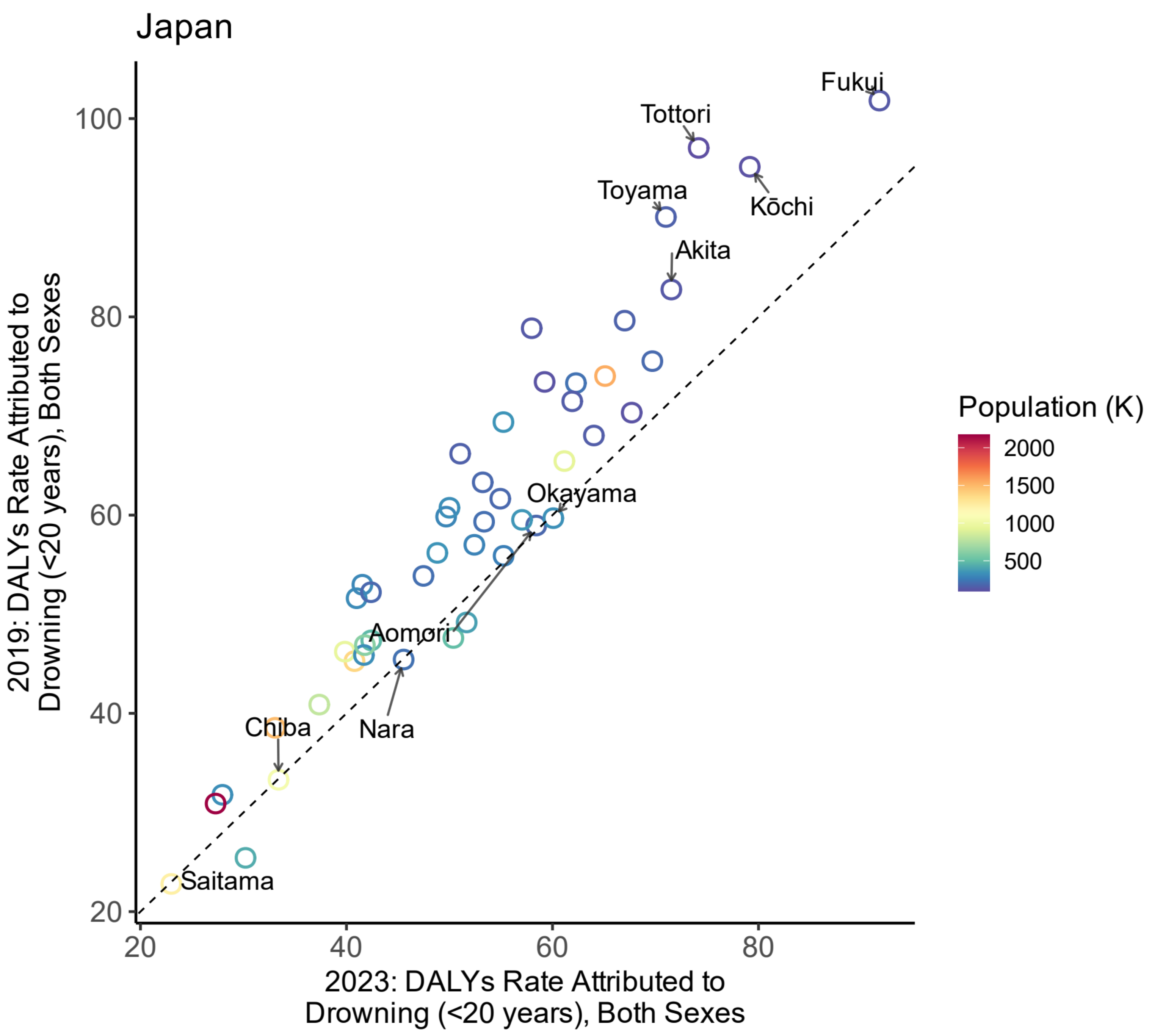
Japan
2019: DALYs Rate Attributed to Drowning (<20 years), Both Sexes
2023: DALYs Rate Attributed to Drowning (<20 years), Both Sexes
Population (K)
2000
1500
1000
500
Fukui
Tottori
Kōchi
Toyama
Akita
Okayama
Aomori
Nara
Chiba
Saitama
100
80
60
40
20
20
40
60
80

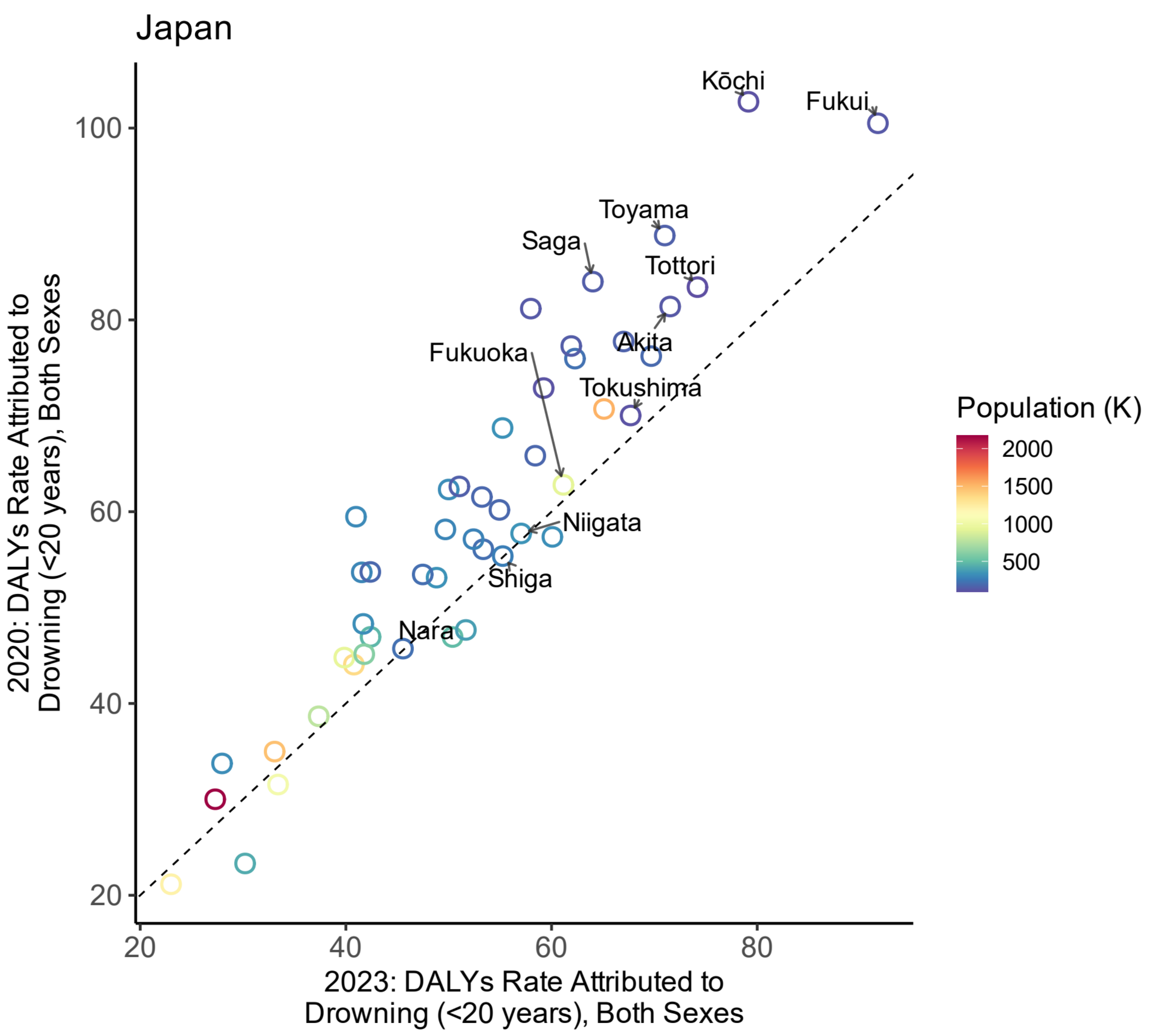
Japan
2020: DALYs Rate Attributed to Drowning (<20 years), Both Sexes
2023: DALYs Rate Attributed to Drowning (<20 years), Both Sexes
Population (K)
2000
1500
1000
500
Kōchi
Fukui
Toyama
Saga
Tottori
Akita
Fukuoka
Tokushima
Niigata
Shiga
Nara
100
80
60
40
20
20
40
60
80

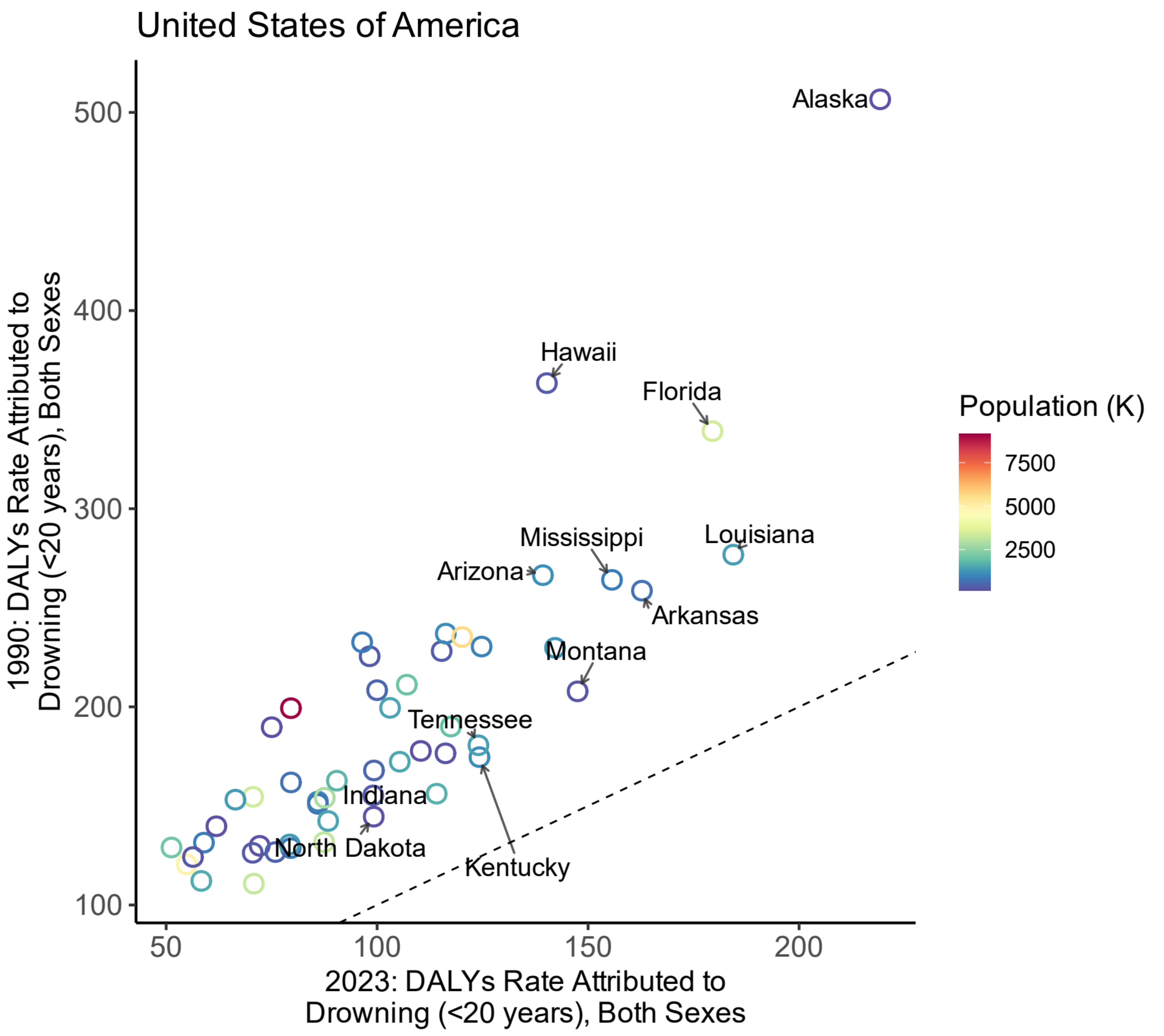
United States of America
1990: DALYs Rate Attributed to Drowning (<20 years), Both Sexes
2023: DALYs Rate Attributed to Drowning (<20 years), Both Sexes
500
400
300
200
100
50
100
150
200
Alaska
Hawaii
Florida
Mississippi
Louisiana
Arizona
Arkansas
Montana
Tennessee
Indiana
North Dakota
Kentucky
Population (K)
7500
5000
2500

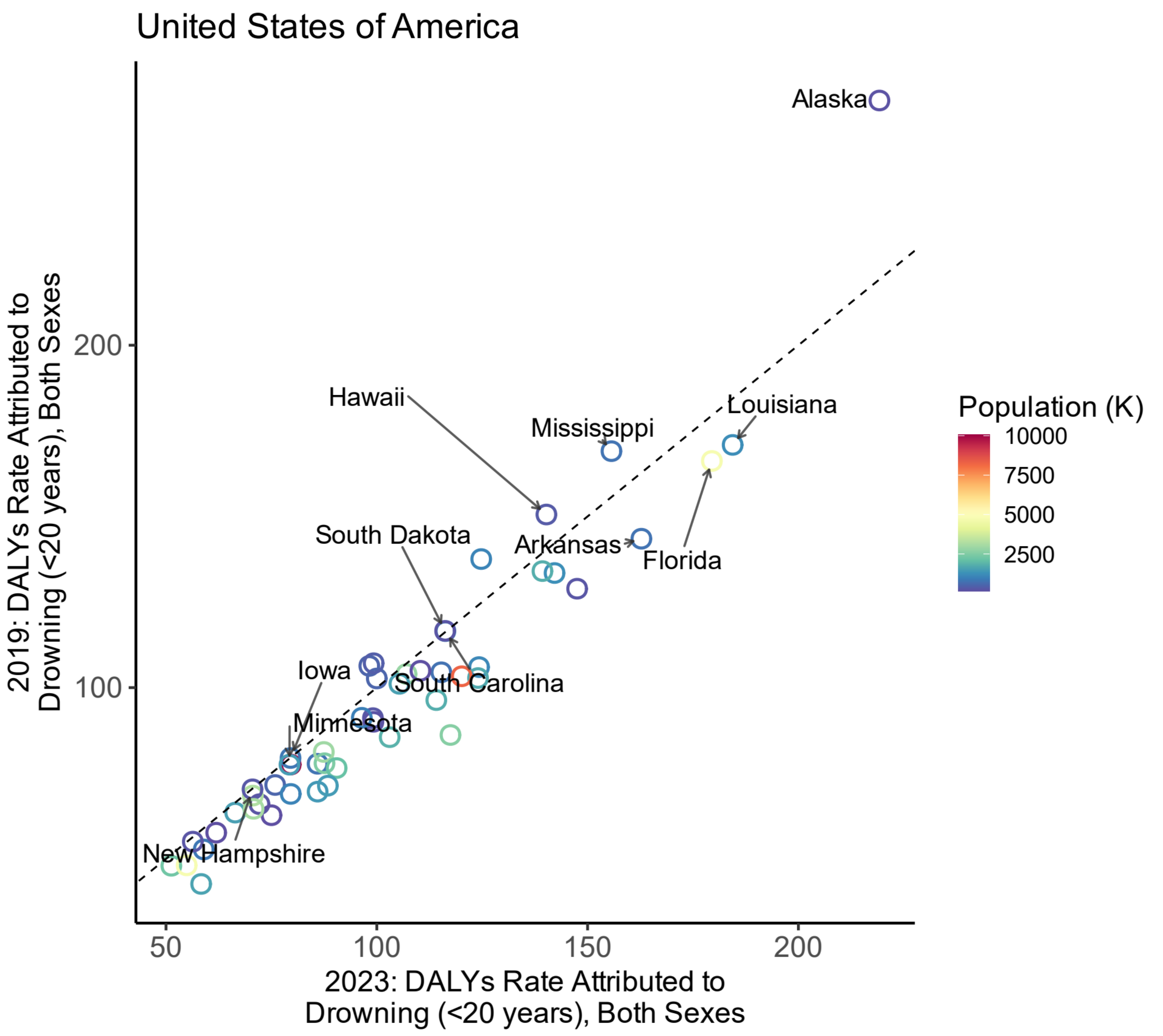
United States of America
2019: DALYs Rate Attributed to Drowning (<20 years), Both Sexes
2023: DALYs Rate Attributed to Drowning (<20 years), Both Sexes
200
100
50
100
150
200
Alaska
Louisiana
Hawaii
Mississippi
South Dakota
Arkansas
Florida
Iowa
South Carolina
Minnesota
New Hampshire
Population (K)
10000
7500
5000
2500

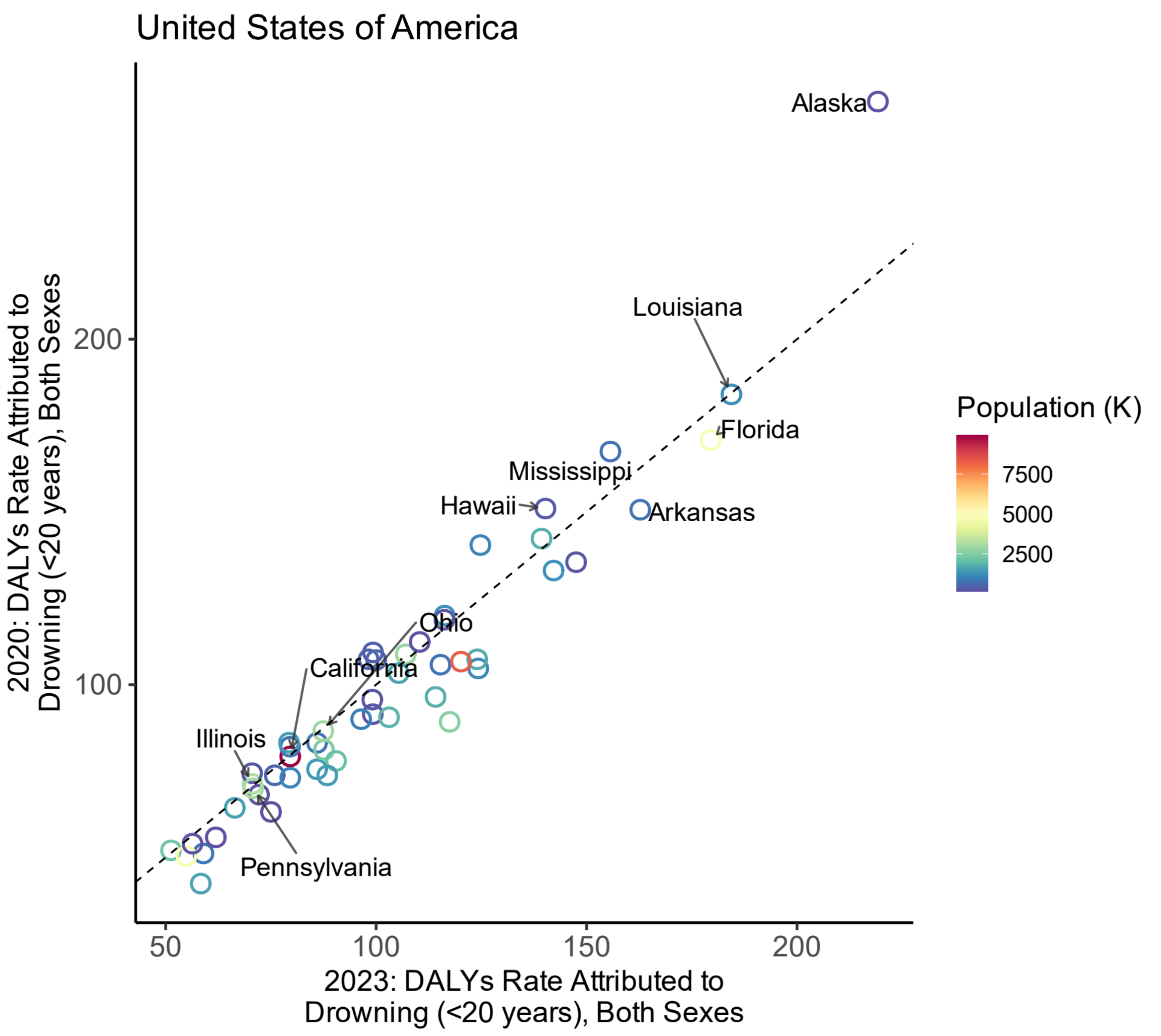
United States of America
2020: DALYs Rate Attributed to Drowning (<20 years), Both Sexes
2023: DALYs Rate Attributed to Drowning (<20 years), Both Sexes
200
100
50
100
150
200
Alaska
Louisiana
Florida
Mississippi
Hawaii
Arkansas
Ohio
California
Illinois
Pennsylvania
Population (K)
7500
5000
2500

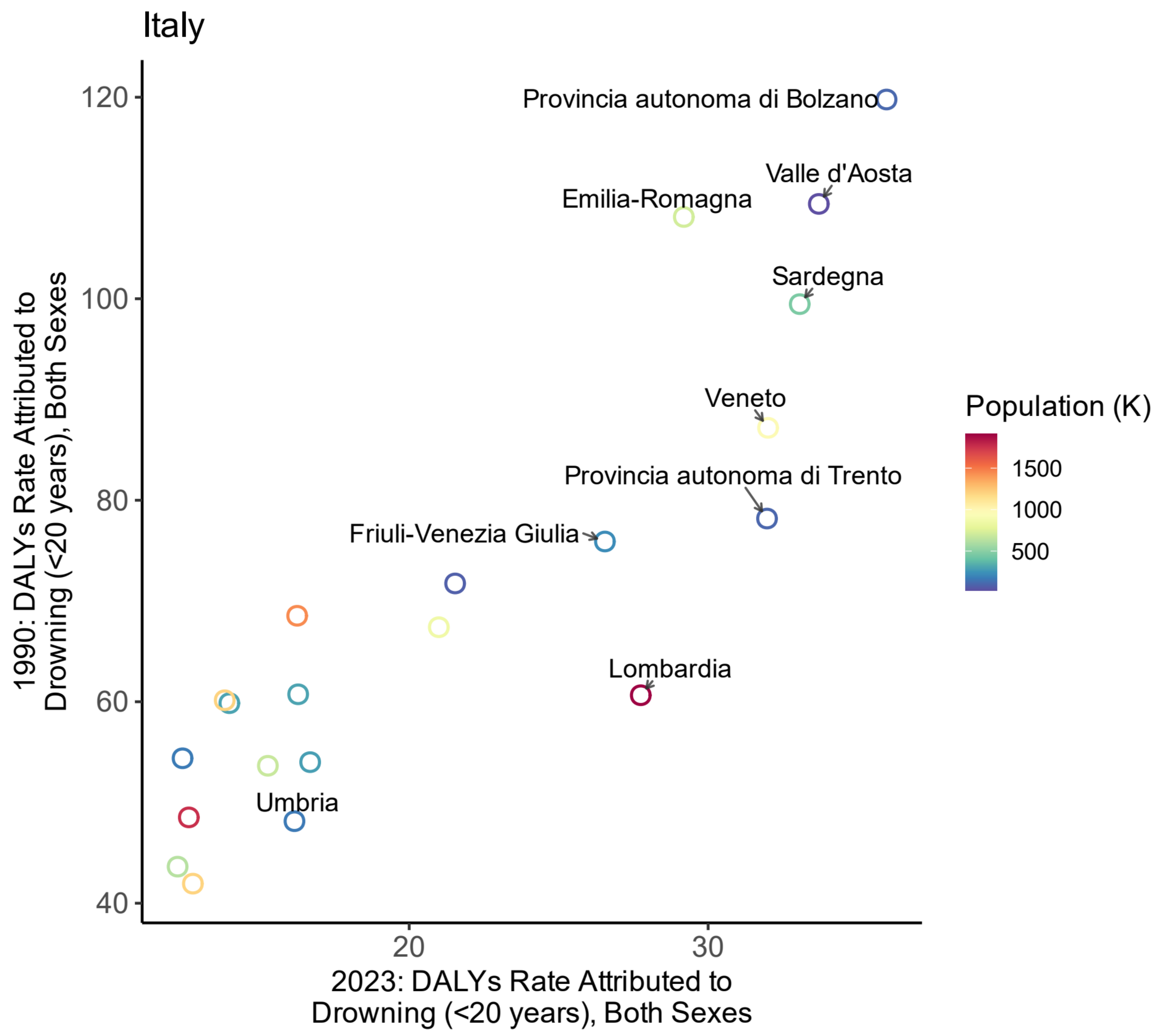
Italy
Provincia autonoma di Bolzano
Valle d'Aosta
Emilia-Romagna
Sardegna
Veneto
Provincia autonoma di Trento
Friuli-Venezia Giulia
Lombardia
Umbria
120
100
80
60
40
20
30
1990: DALYs Rate Attributed to Drowning (<20 years), Both Sexes
2023: DALYs Rate Attributed to Drowning (<20 years), Both Sexes
Population (K)
1500
1000
500

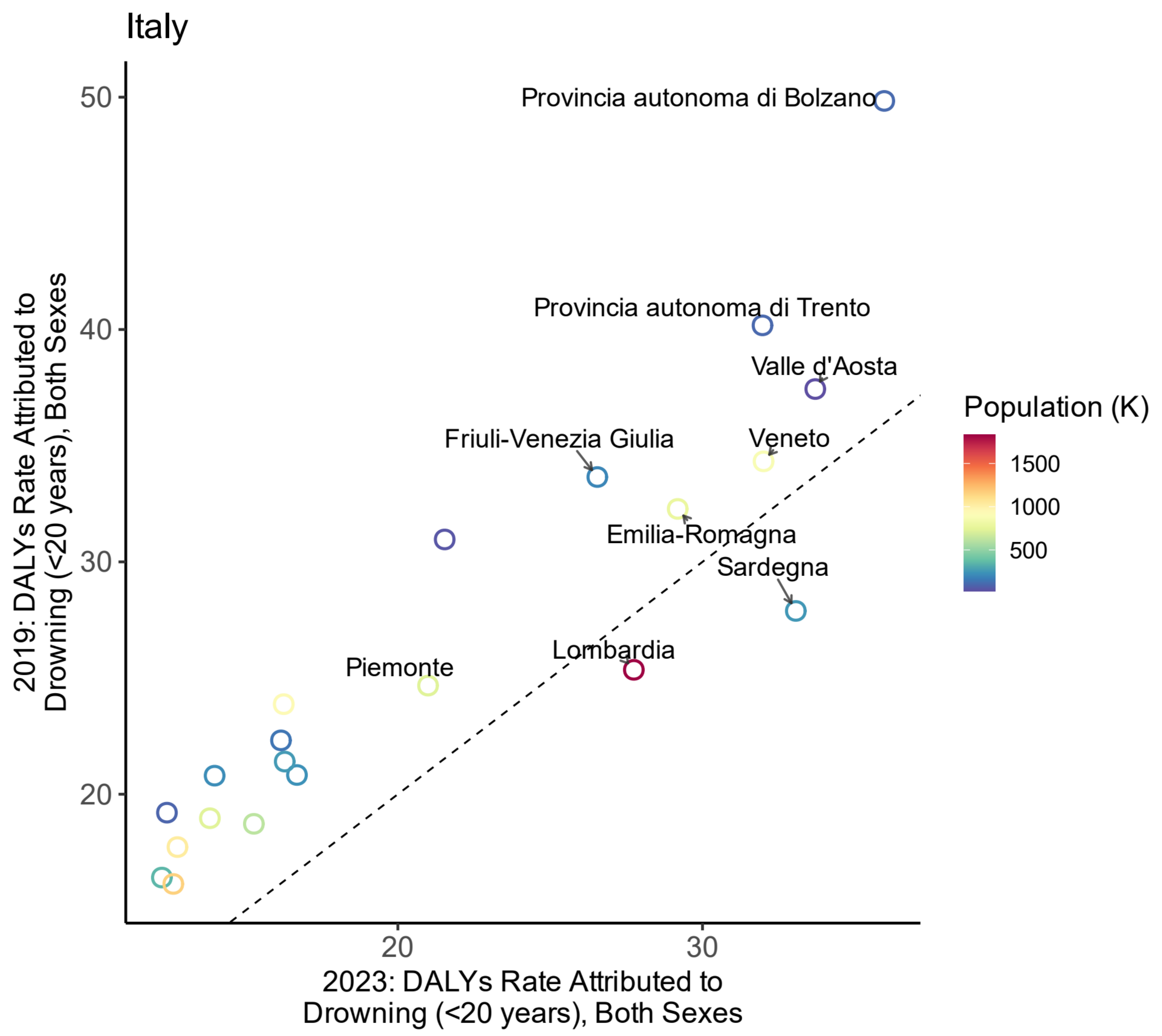
Italy
Provincia autonoma di Bolzano
Provincia autonoma di Trento
Valle d'Aosta
Friuli-Venezia Giulia
Veneto
Emilia-Romagna
Sardegna
Lombardia
Piemonte
50
40
30
20
20
30
2019: DALYs Rate Attributed to Drowning (<20 years), Both Sexes
2023: DALYs Rate Attributed to Drowning (<20 years), Both Sexes
Population (K)
1500
1000
500

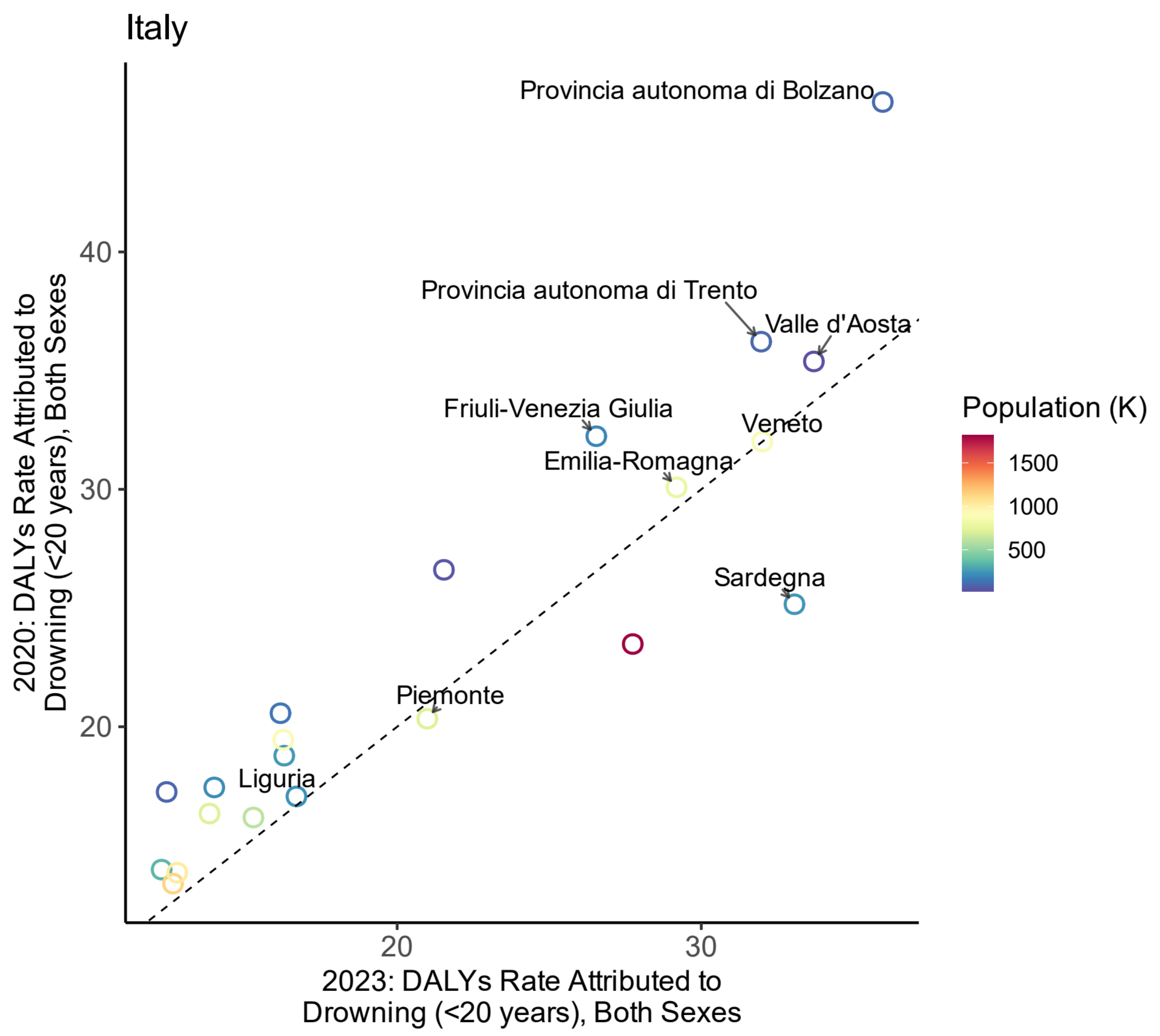
Italy
Provincia autonoma di Bolzano
Provincia autonoma di Trento
Valle d'Aosta
Friuli-Venezia Giulia
Veneto
Emilia-Romagna
Sardegna
Piemonte
Liguria
40
30
20
20
30
2020: DALYs Rate Attributed to Drowning (<20 years), Both Sexes
2023: DALYs Rate Attributed to Drowning (<20 years), Both Sexes
Population (K)
1500
1000
500

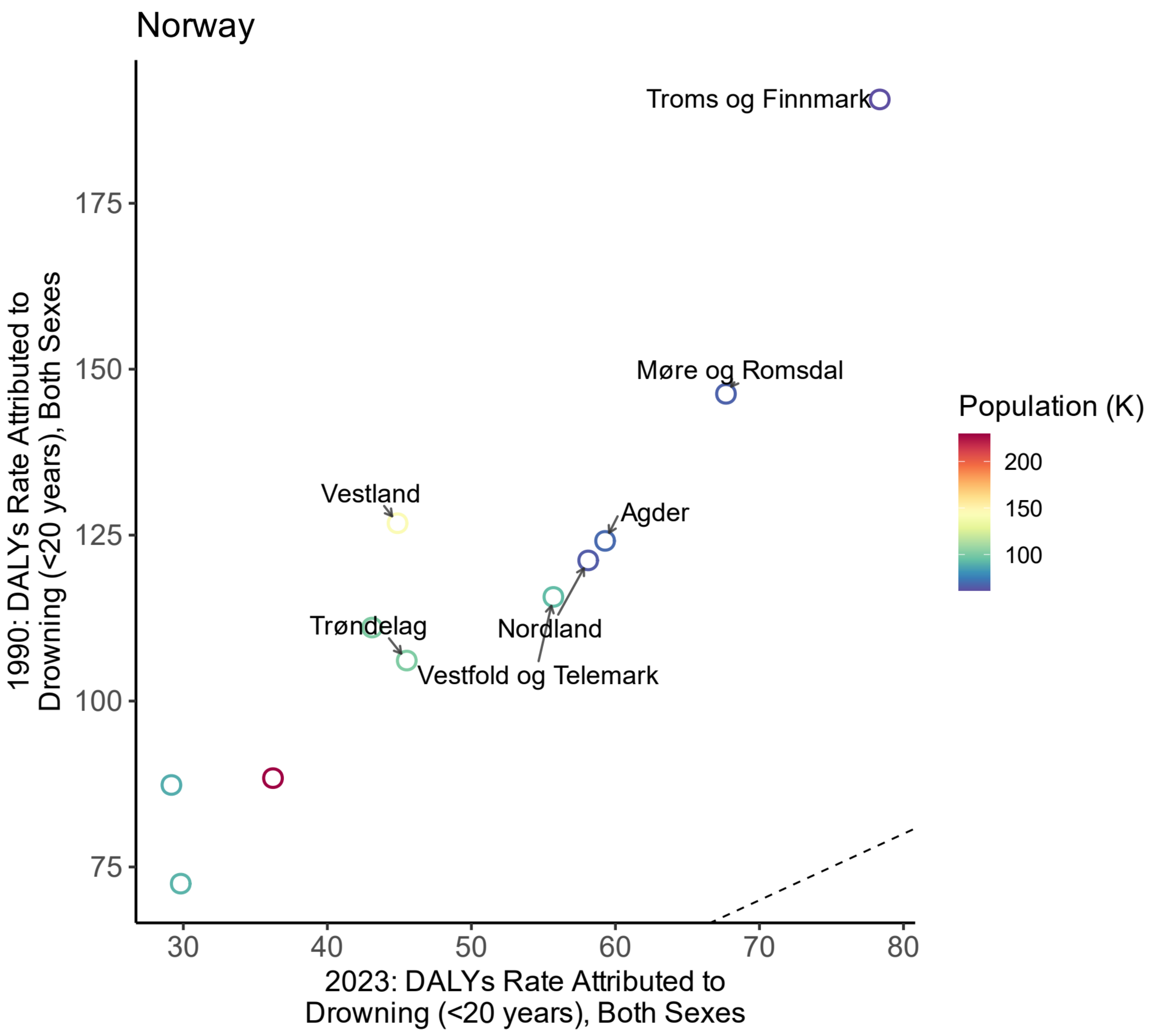
Norway
Troms og Finnmark
Møre og Romsdal
Vestland
Agder
Trøndelag
Nordland
Vestfold og Telemark
1990: DALYs Rate Attributed to Drowning (<20 years), Both Sexes
2023: DALYs Rate Attributed to Drowning (<20 years), Both Sexes
Population (K)
200
150
100
175
125
75
30
40
50
60
70
80

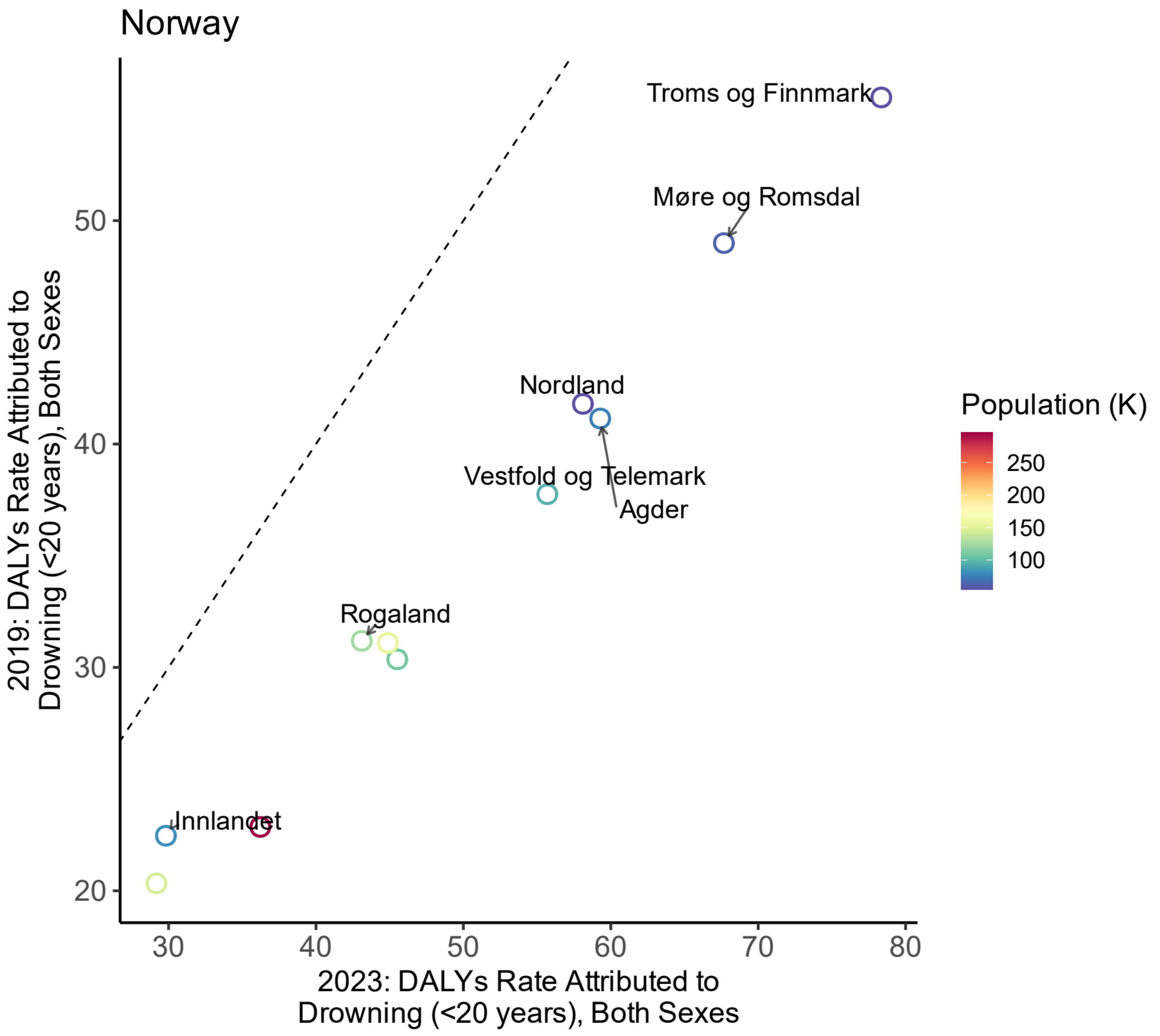
Norway
Troms og Finnmark
Møre og Romsdal
Nordland
Vestfold og Telemark
Agder
Rogaland
Innlandet
2019: DALYs Rate Attributed to Drowning (<20 years), Both Sexes
2023: DALYs Rate Attributed to Drowning (<20 years), Both Sexes
Population (K)
250
200
150
100
50
40
30
20
30
40
50
60
70
80

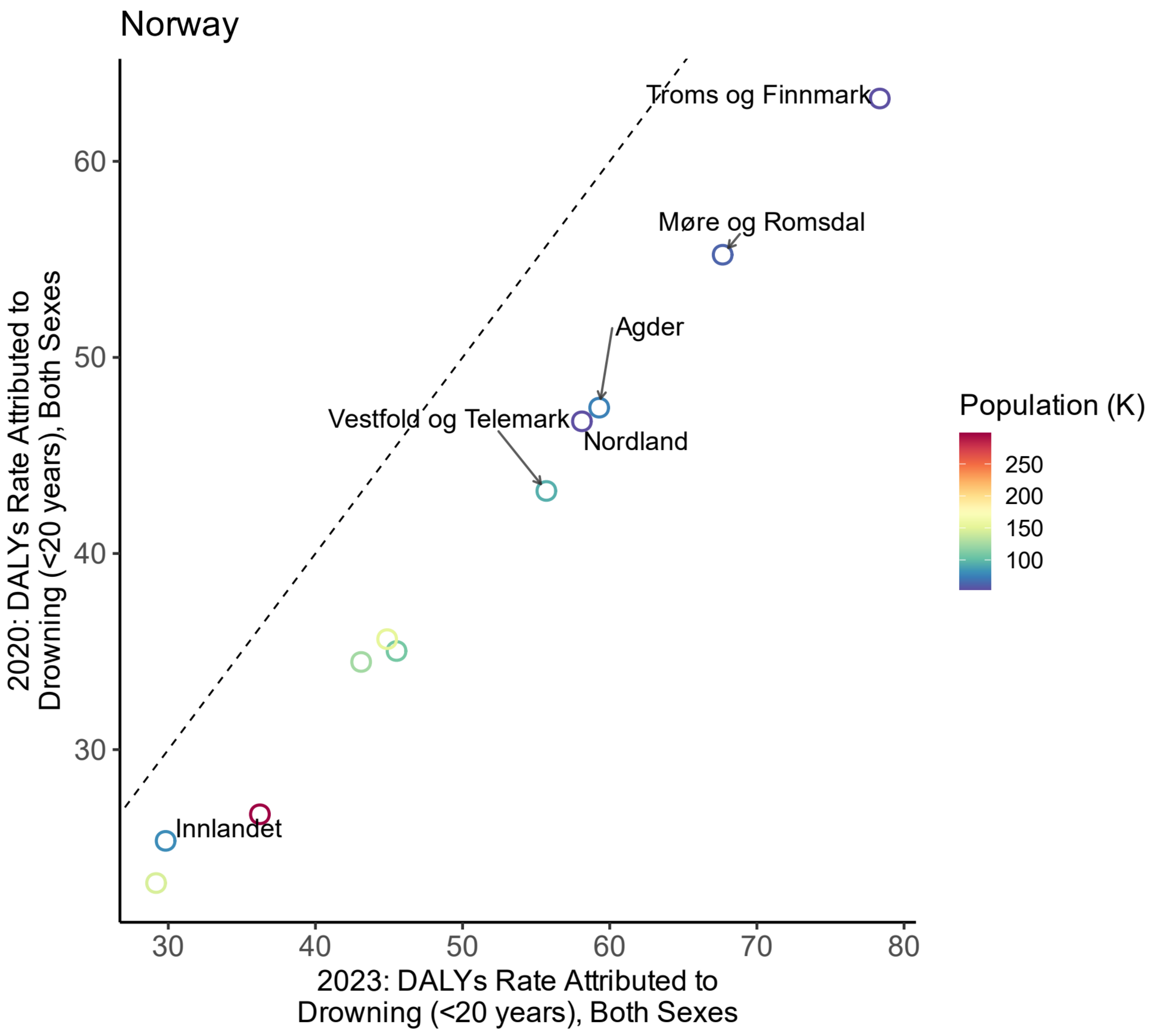
Norway
Troms og Finnmark
Møre og Romsdal
Agder
Vestfold og Telemark
Nordland
Innlandet
2020: DALYs Rate Attributed to Drowning (<20 years), Both Sexes
2023: DALYs Rate Attributed to Drowning (<20 years), Both Sexes
Population (K)
250
200
150
100
60
50
40
30
30
40
50
60
70
80

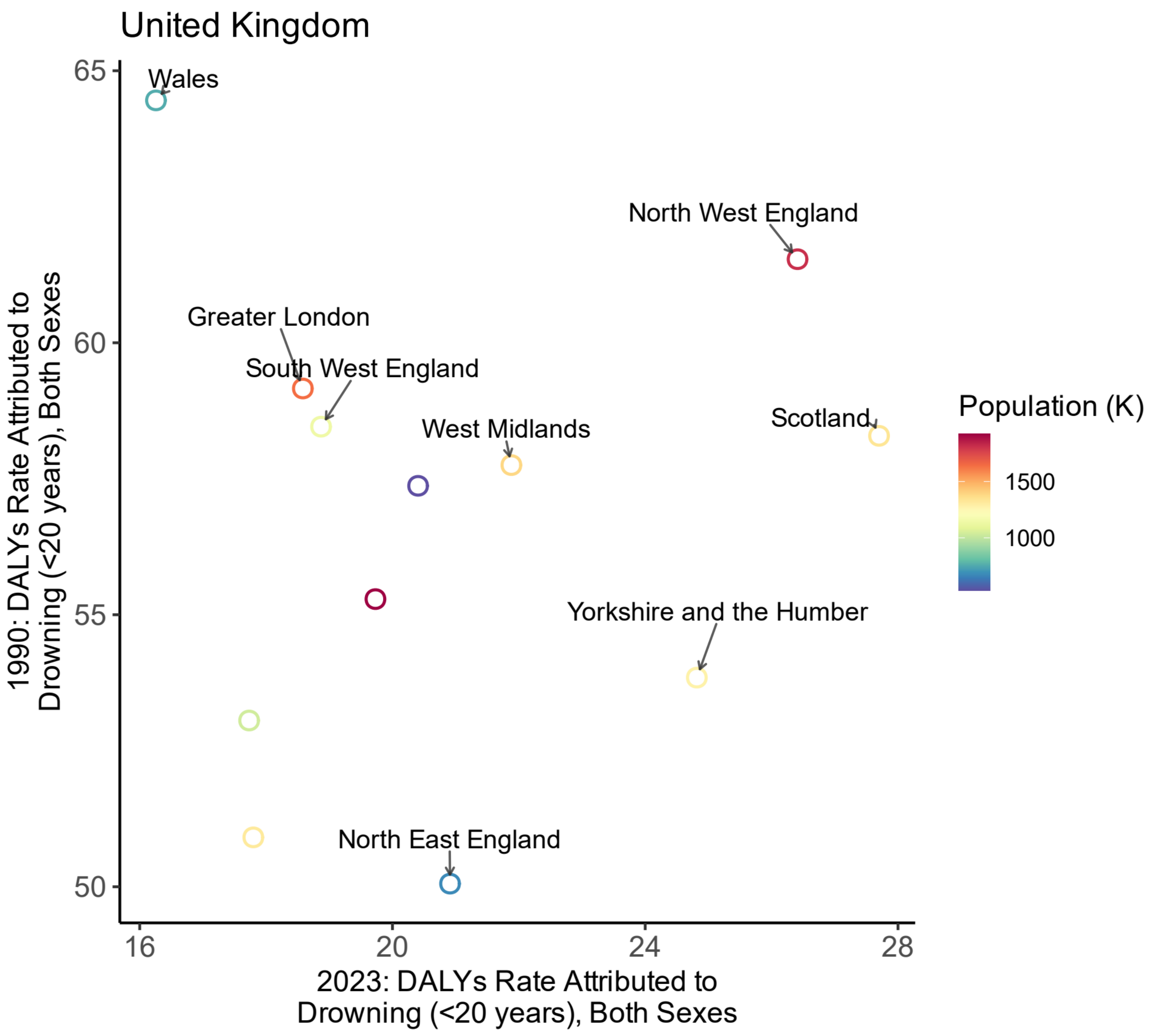
United Kingdom
Wales
North West England
Greater London
South West England
West Midlands
Scotland
Yorkshire and the Humber
North East England
Population (K)
1500
1000
65
60
55
50
16
20
24
28
1990: DALYs Rate Attributed to Drowning (<20 years), Both Sexes
2023: DALYs Rate Attributed to Drowning (<20 years), Both Sexes

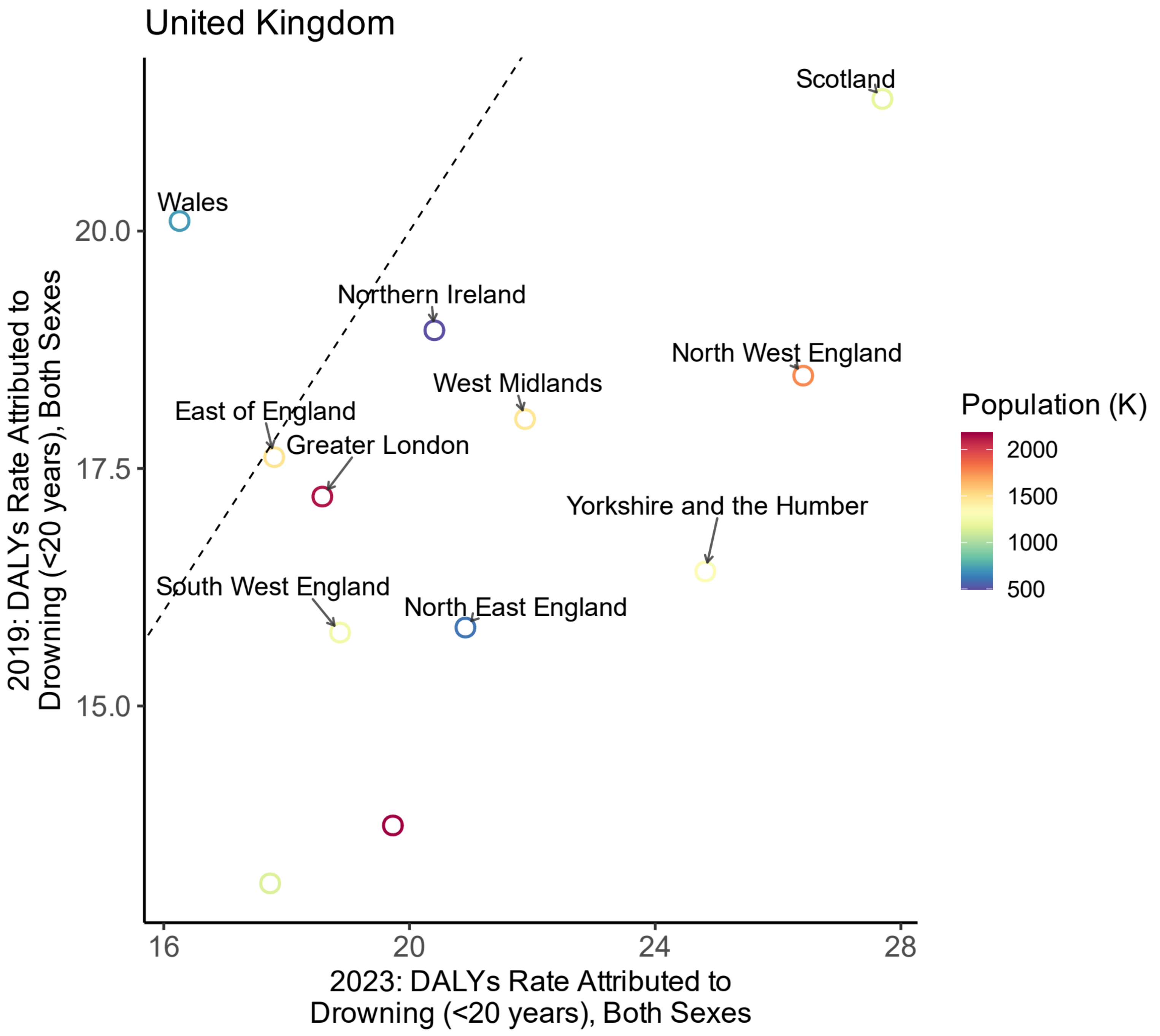
United Kingdom
Scotland
Wales
Northern Ireland
North West England
West Midlands
East of England
Greater London
Yorkshire and the Humber
South West England
North East England
Population (K)
2000
1500
1000
500
20.0
17.5
15.0
16
20
24
28
2019: DALYs Rate Attributed to Drowning (<20 years), Both Sexes
2023: DALYs Rate Attributed to Drowning (<20 years), Both Sexes

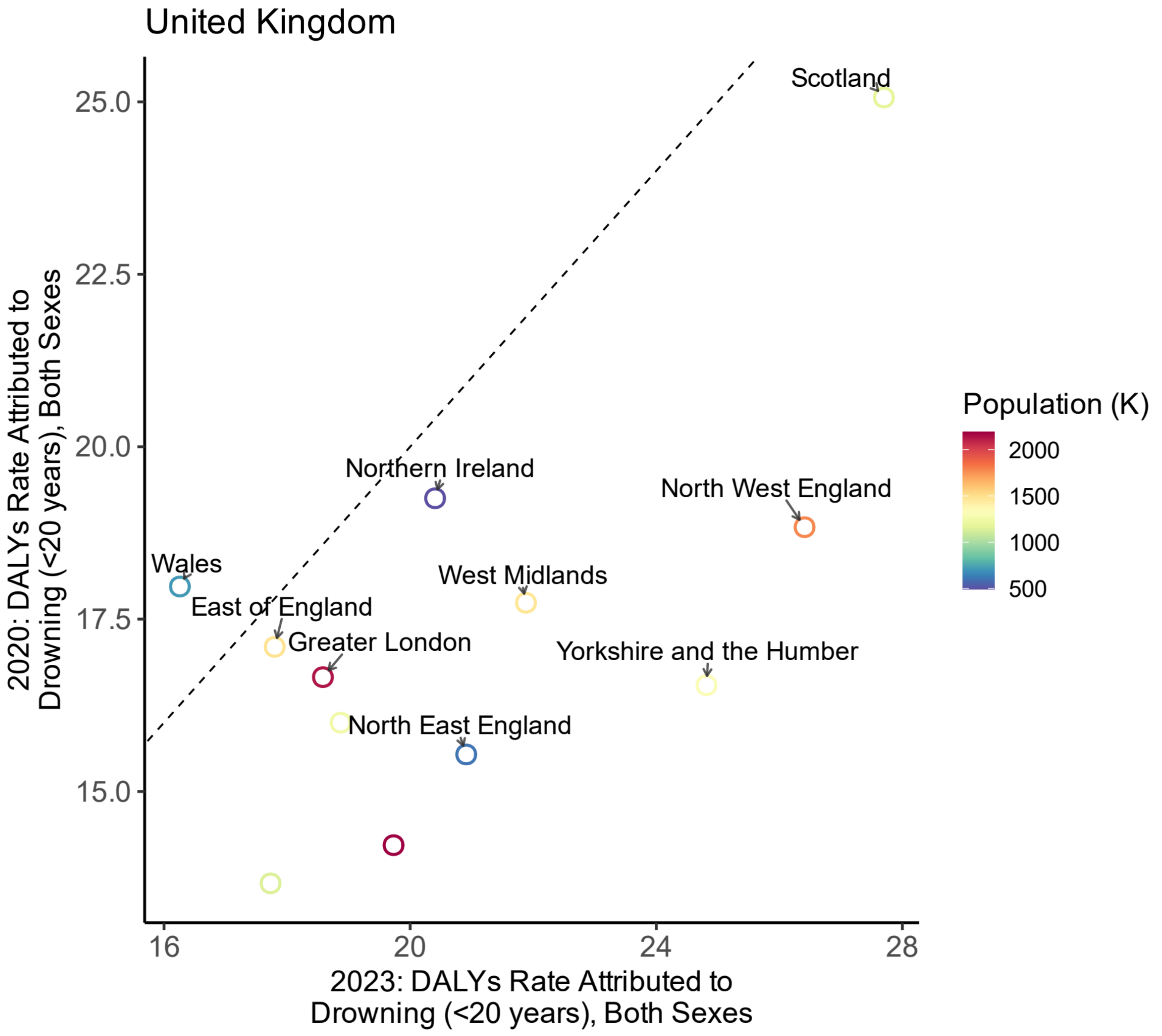
United Kingdom
Scotland
Northern Ireland
North West England
Wales
West Midlands
East of England
Greater London
Yorkshire and the Humber
North East England
Population (K)
2000
1500
1000
500
25.0
22.5
20.0
17.5
15.0
16
20
24
28
2020: DALYs Rate Attributed to Drowning (<20 years), Both Sexes
2023: DALYs Rate Attributed to Drowning (<20 years), Both Sexes

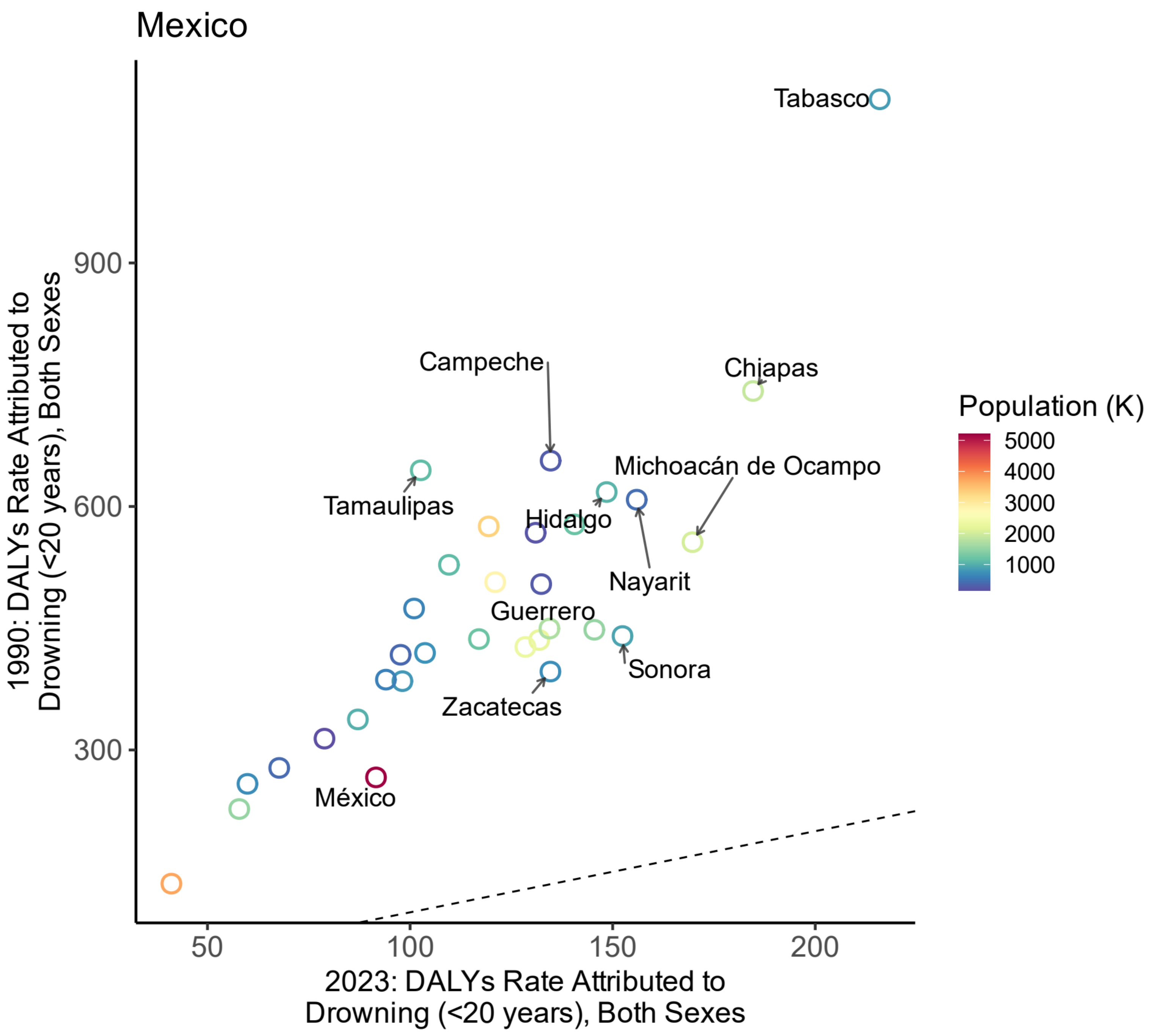
Mexico
1990: DALYs Rate Attributed to Drowning (<20 years), Both Sexes
2023: DALYs Rate Attributed to Drowning (<20 years), Both Sexes
Population (K)
5000
4000
3000
2000
1000
900
600
300
50
100
150
200
Tabasco
Campeche
Chiapas
Michoacán de Ocampo
Tamaulipas
Hidalgo
Nayarit
Guerrero
Sonora
Zacatecas
México

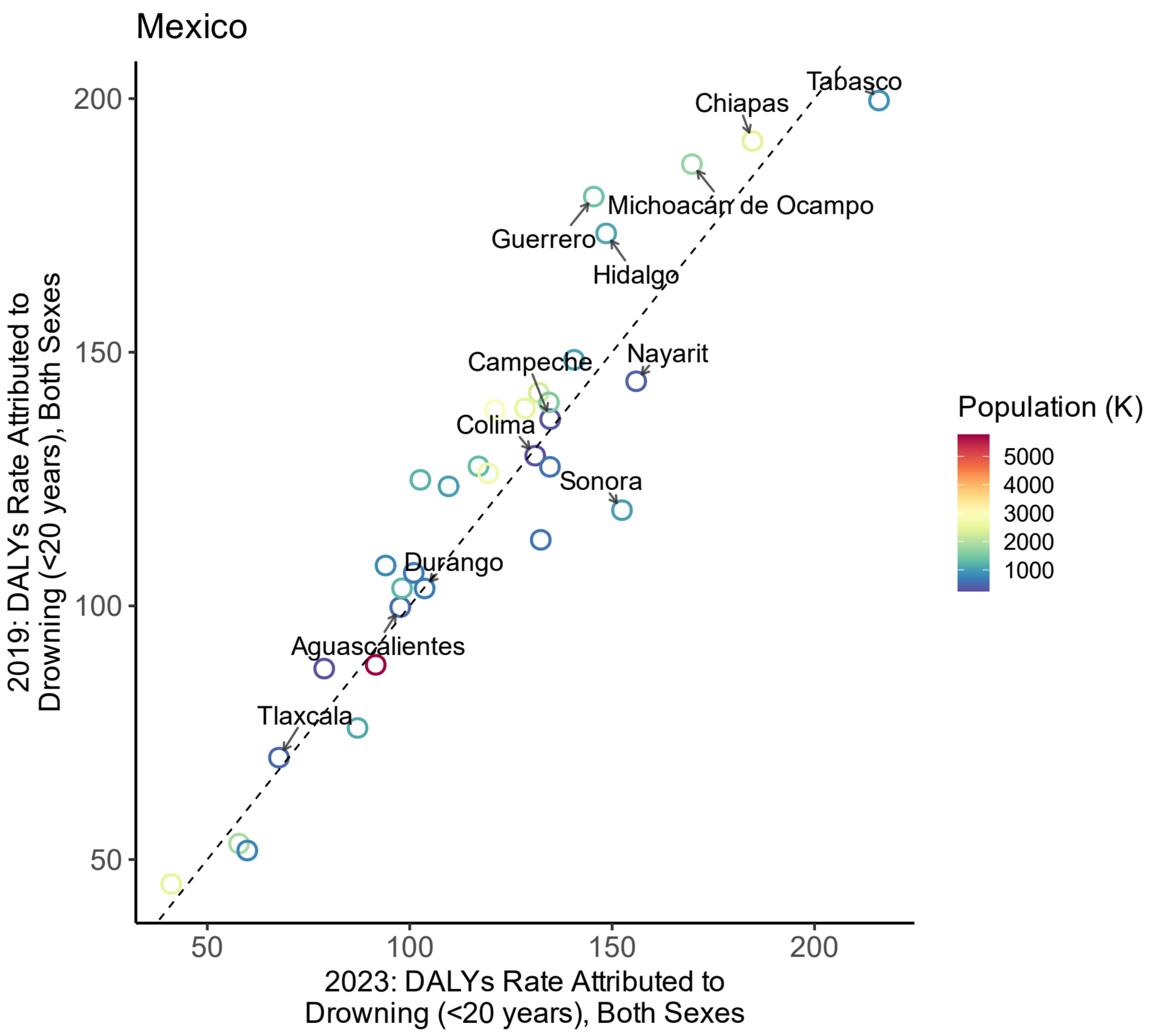
Mexico
2019: DALYs Rate Attributed to Drowning (<20 years), Both Sexes
2023: DALYs Rate Attributed to Drowning (<20 years), Both Sexes
Population (K)
5000
4000
3000
2000
1000
200
150
100
50
Tabasco
Chiapas
Michoacán de Ocampo
Guerrero
Hidalgo
Campeche
Nayarit
Colima
Sonora
Durango
Aguascalientes
Tlaxcala

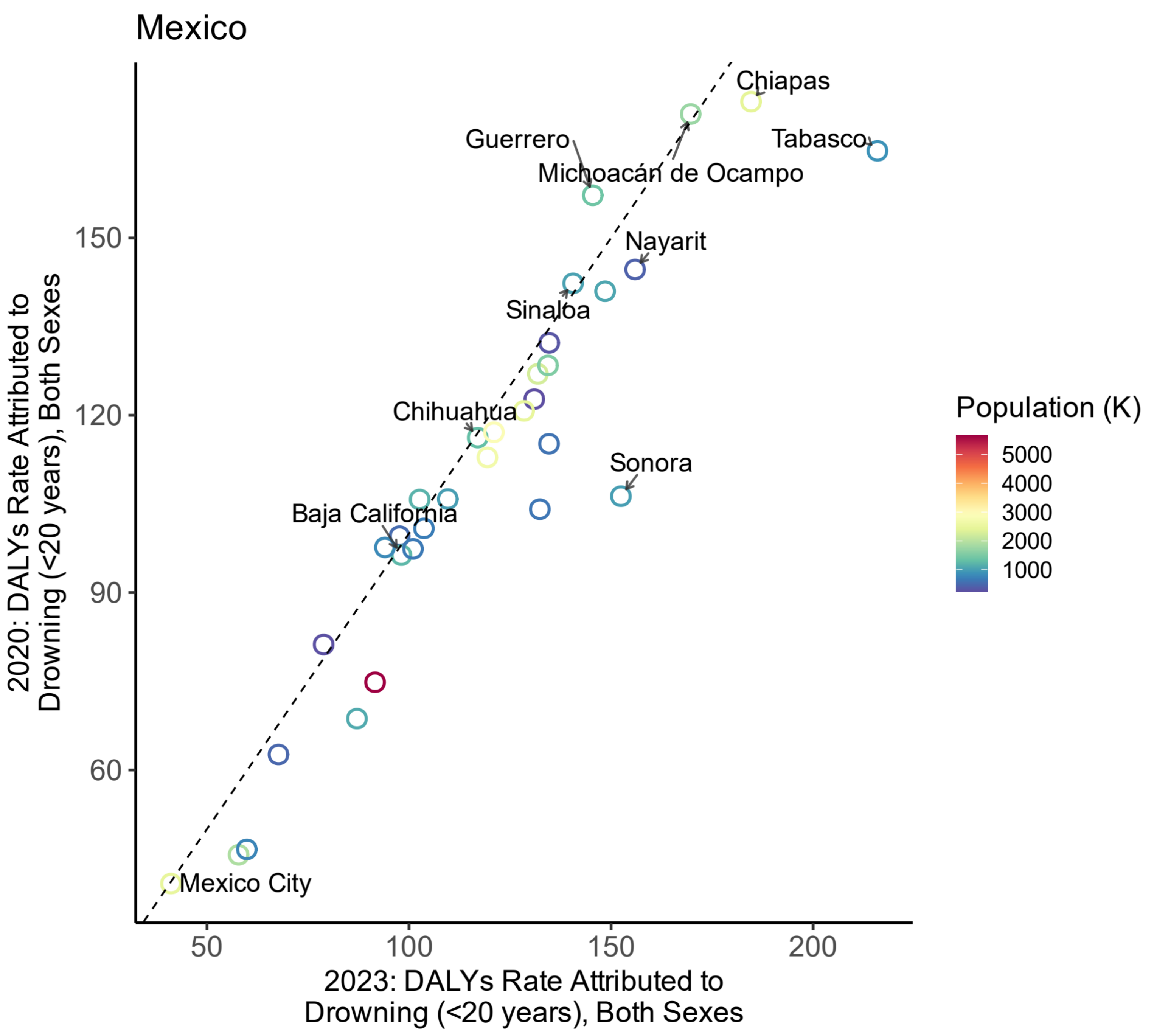
Mexico
2020: DALYs Rate Attributed to Drowning (<20 years), Both Sexes
2023: DALYs Rate Attributed to Drowning (<20 years), Both Sexes
Population (K)
5000
4000
3000
2000
1000
150
120
90
60
50
100
150
200
Chiapas
Guerrero
Tabasco
Michoacán de Ocampo
Nayarit
Sinaloa
Chihuahua
Sonora
Baja California
Mexico City

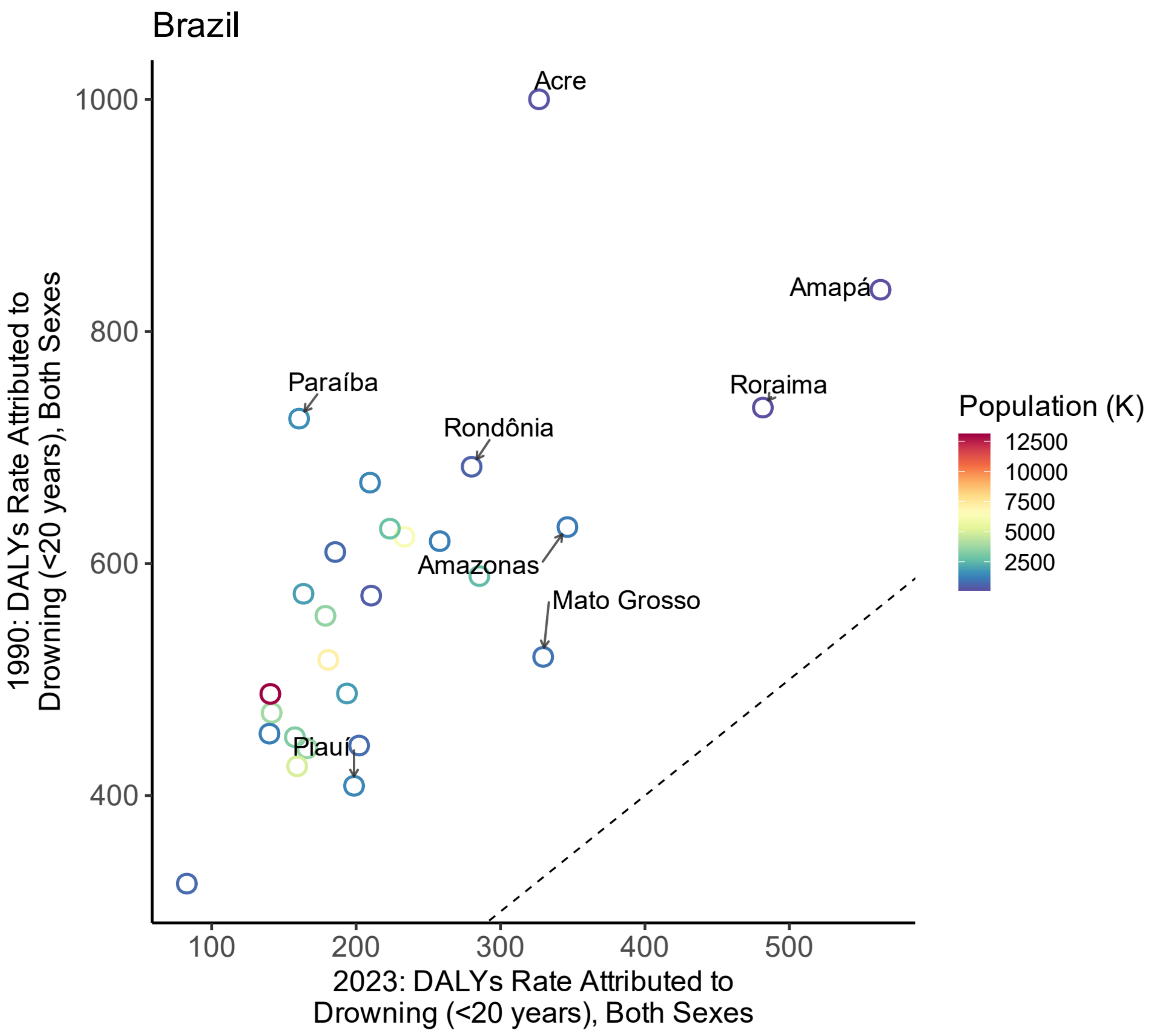
Brazil
1990: DALYs Rate Attributed to Drowning (<20 years), Both Sexes
2023: DALYs Rate Attributed to Drowning (<20 years), Both Sexes
Population (K)
12500
10000
7500
5000
2500
1000
800
600
400
100
200
300
400
500
Acre
Amapá
Roraima
Paraíba
Rondônia
Amazonas
Mato Grosso
Piauí

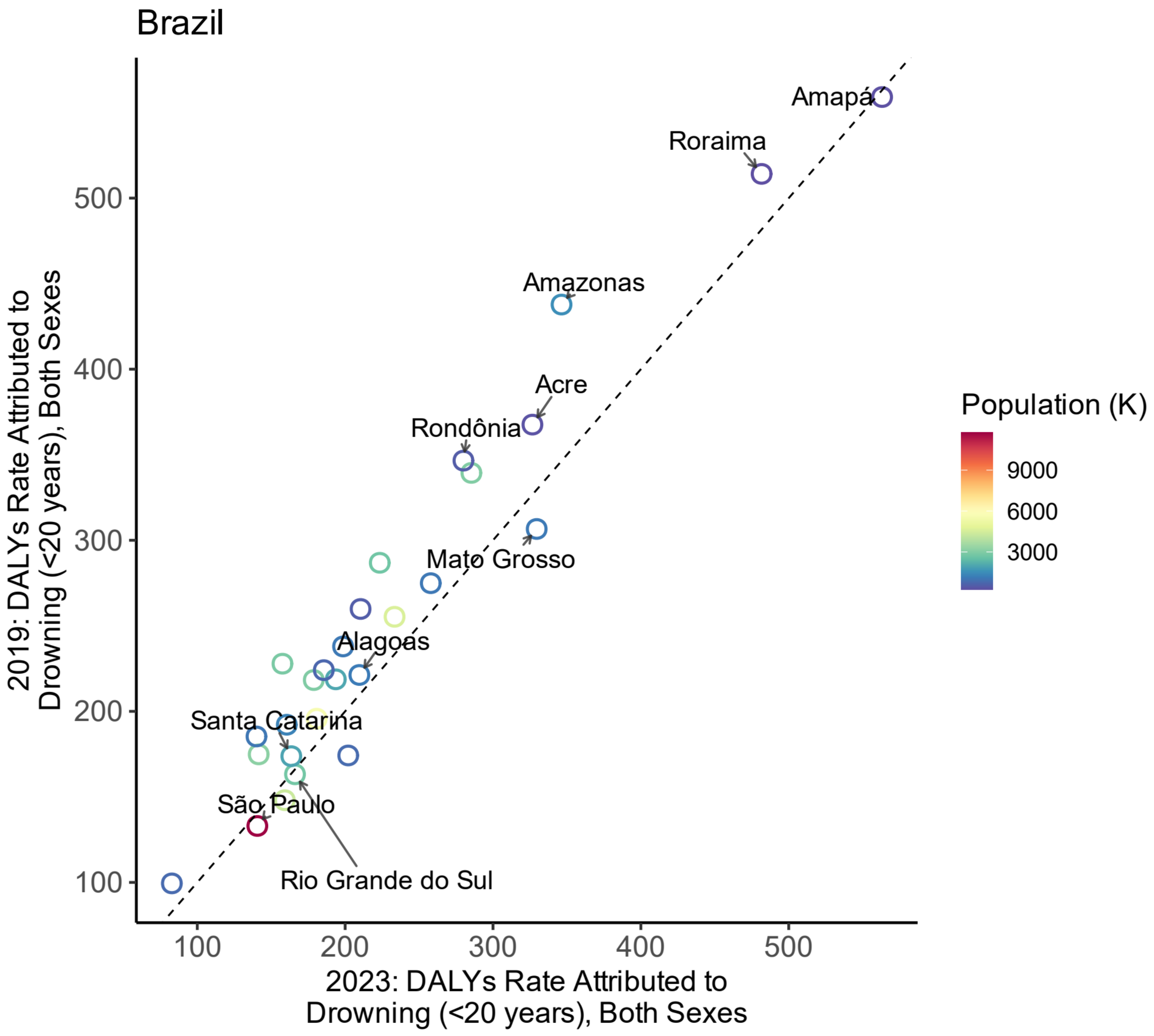
Brazil
2019: DALYs Rate Attributed to Drowning (<20 years), Both Sexes
2023: DALYs Rate Attributed to Drowning (<20 years), Both Sexes
Population (K)
9000
6000
3000
500
400
300
200
100
100
200
300
400
500
Amapá
Roraima
Amazonas
Acre
Rondônia
Mato Grosso
Alagoas
Santa Catarina
São Paulo
Rio Grande do Sul

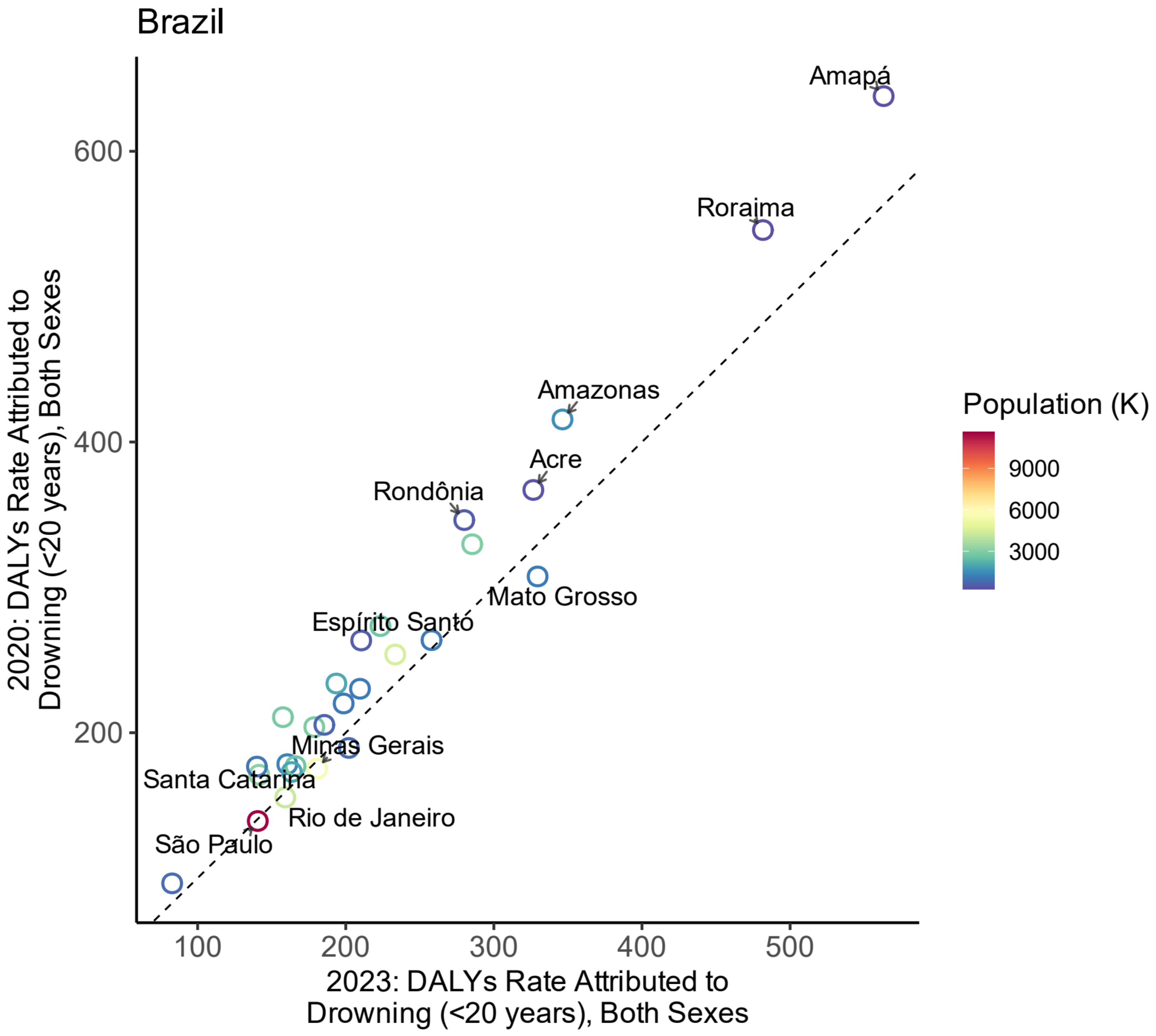
Brazil
2020: DALYs Rate Attributed to Drowning (<20 years), Both Sexes
2023: DALYs Rate Attributed to Drowning (<20 years), Both Sexes
Population (K)
9000
6000
3000
600
400
200
100
200
300
400
500
Amapá
Roraima
Amazonas
Acre
Rondônia
Mato Grosso
Espírito Santo
Minas Gerais
Santa Catarina
Rio de Janeiro
São Paulo

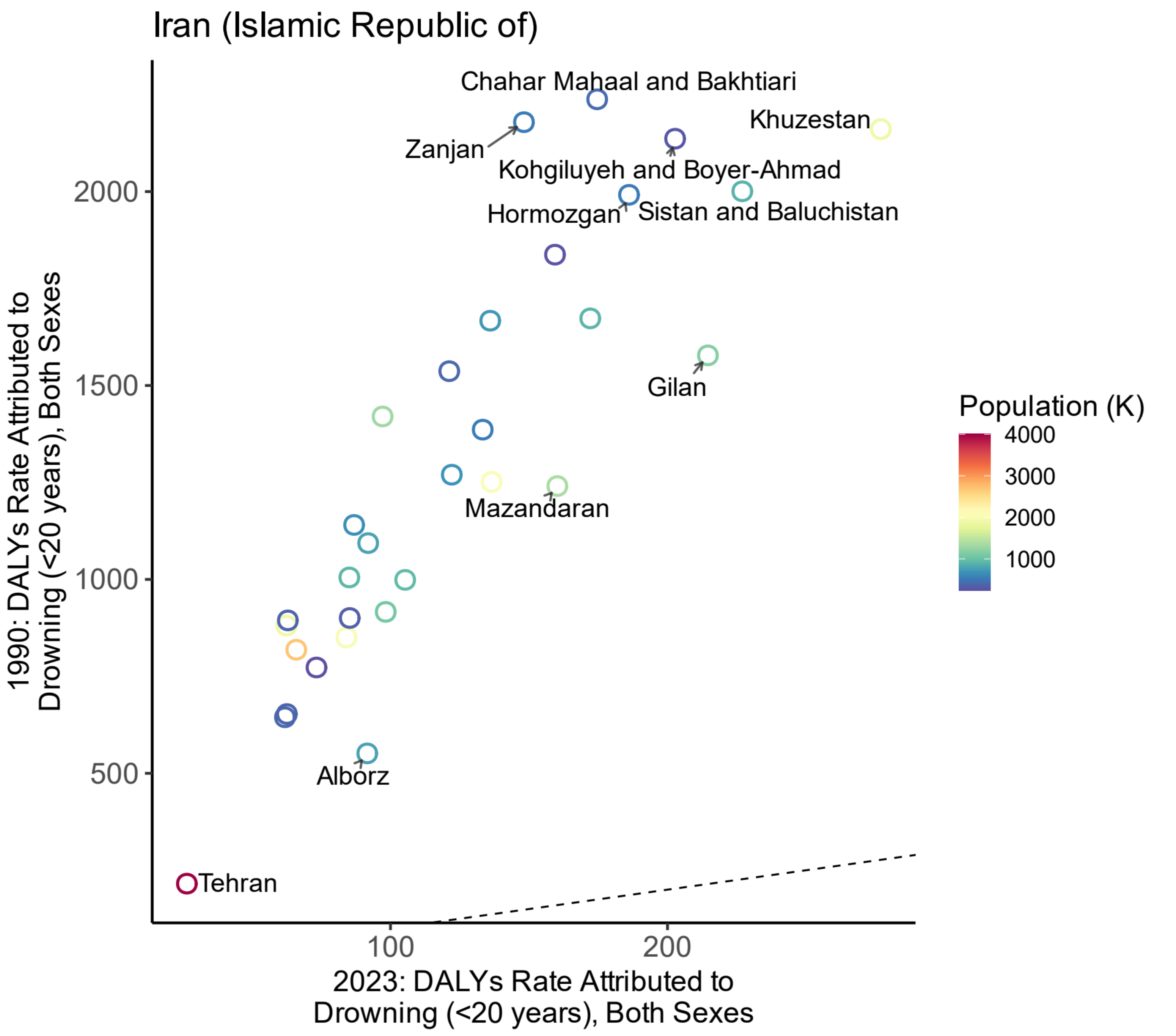
Iran (Islamic Republic of)
Chahar Mahaal and Bakhtiari
Khuzestan
Zanjan
Kohgiluyeh and Boyer-Ahmad
Hormozgan
Sistan and Baluchistan
Gilan
Mazandaran
Alborz
Tehran
1990: DALYs Rate Attributed to Drowning (<20 years), Both Sexes
2023: DALYs Rate Attributed to Drowning (<20 years), Both Sexes
Population (K)
4000
3000
2000
1000
500
1500
100
200

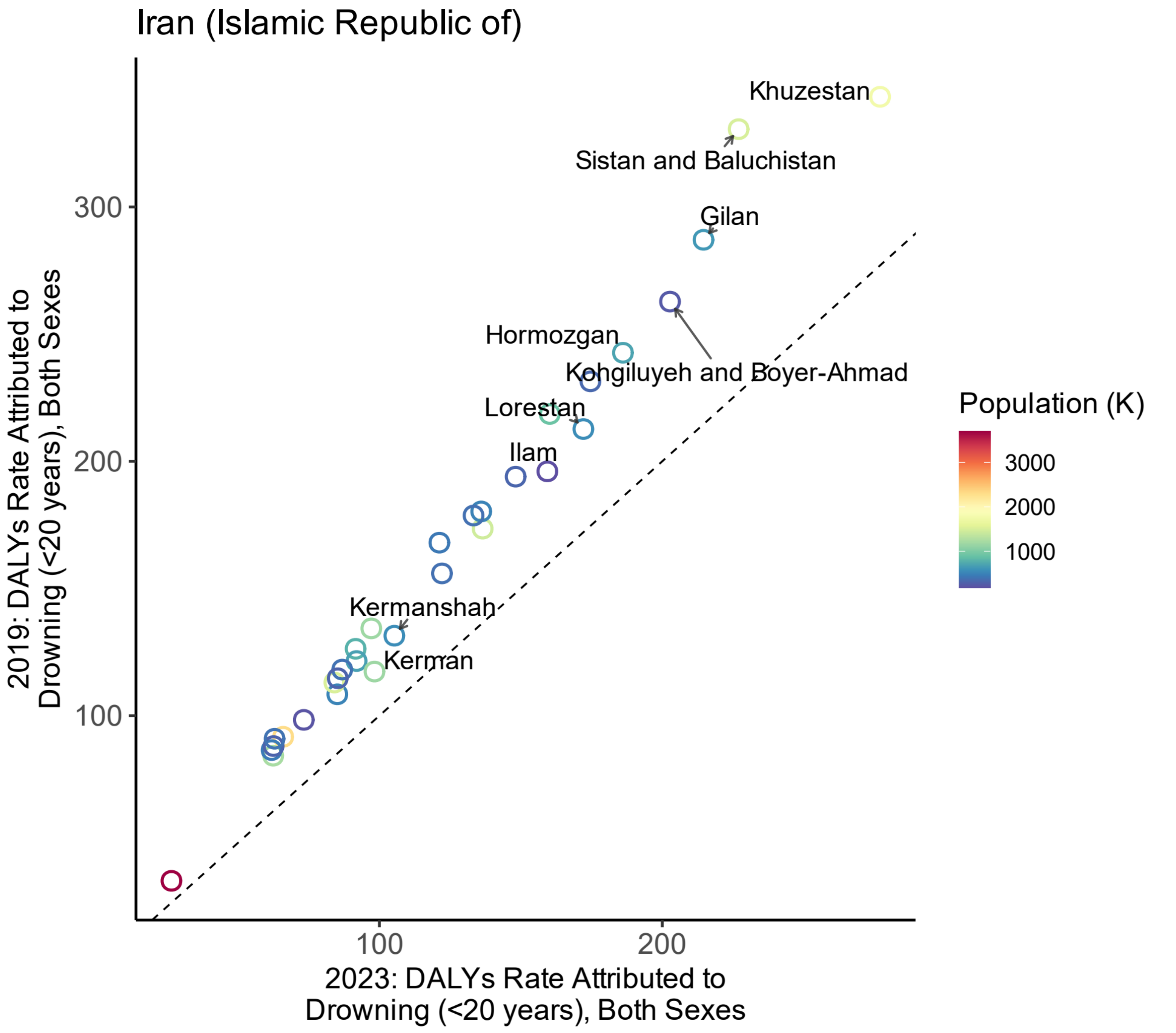
Iran (Islamic Republic of)
Khuzestan
Sistan and Baluchistan
Gilan
Hormozgan
Kohgiluyeh and Boyer-Ahmad
Lorestan
Ilam
Kermanshah
Kerman
2019: DALYs Rate Attributed to Drowning (<20 years), Both Sexes
2023: DALYs Rate Attributed to Drowning (<20 years), Both Sexes
Population (K)
3000
2000
1000
300
200
100

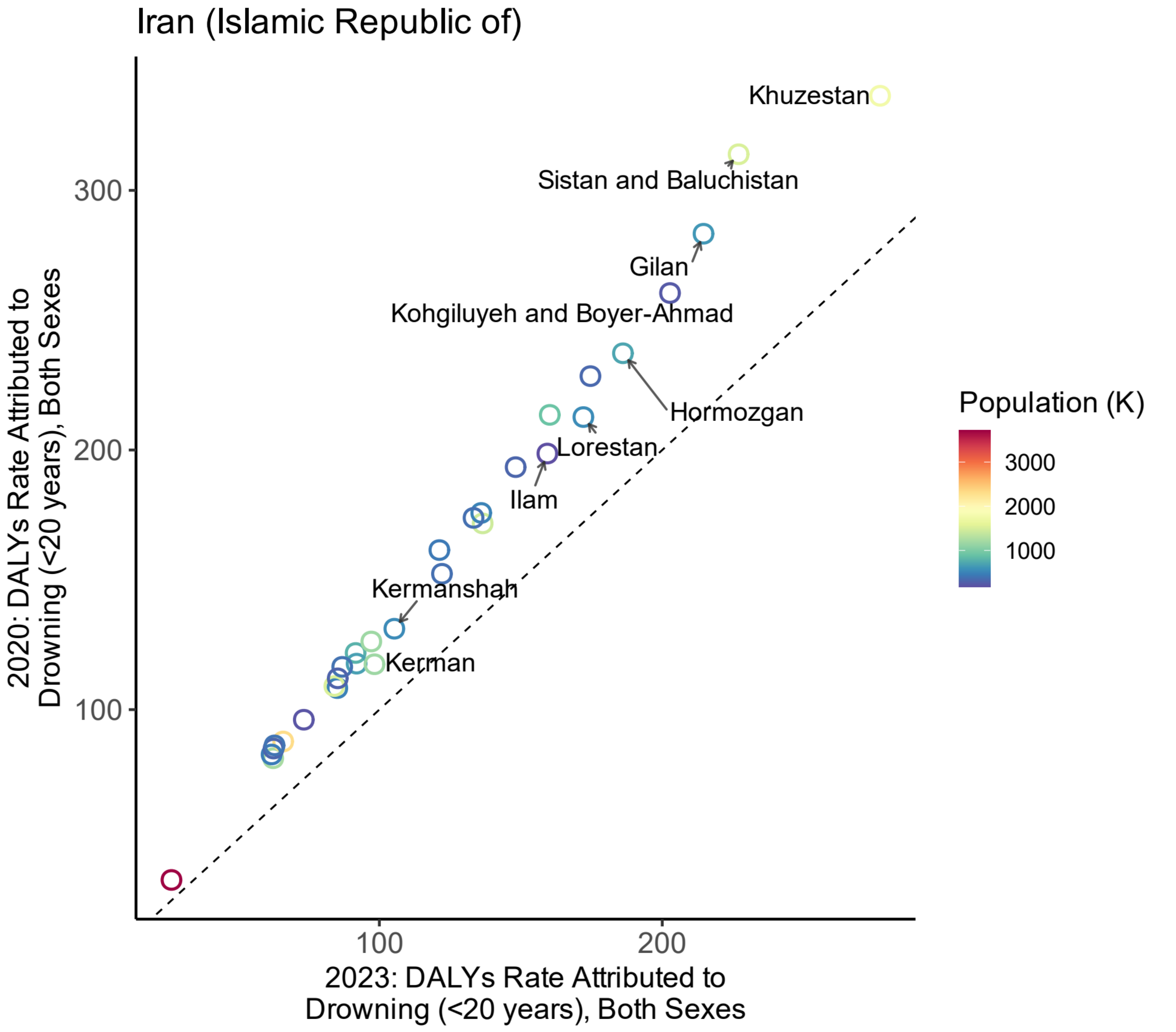
Iran (Islamic Republic of)
Khuzestan
Sistan and Baluchistan
Gilan
Kohgiluyeh and Boyer-Ahmad
Hormozgan
Lorestan
Ilam
Kermanshah
Kerman
2020: DALYs Rate Attributed to Drowning (<20 years), Both Sexes
2023: DALYs Rate Attributed to Drowning (<20 years), Both Sexes
Population (K)
3000
2000
1000
300
200
100

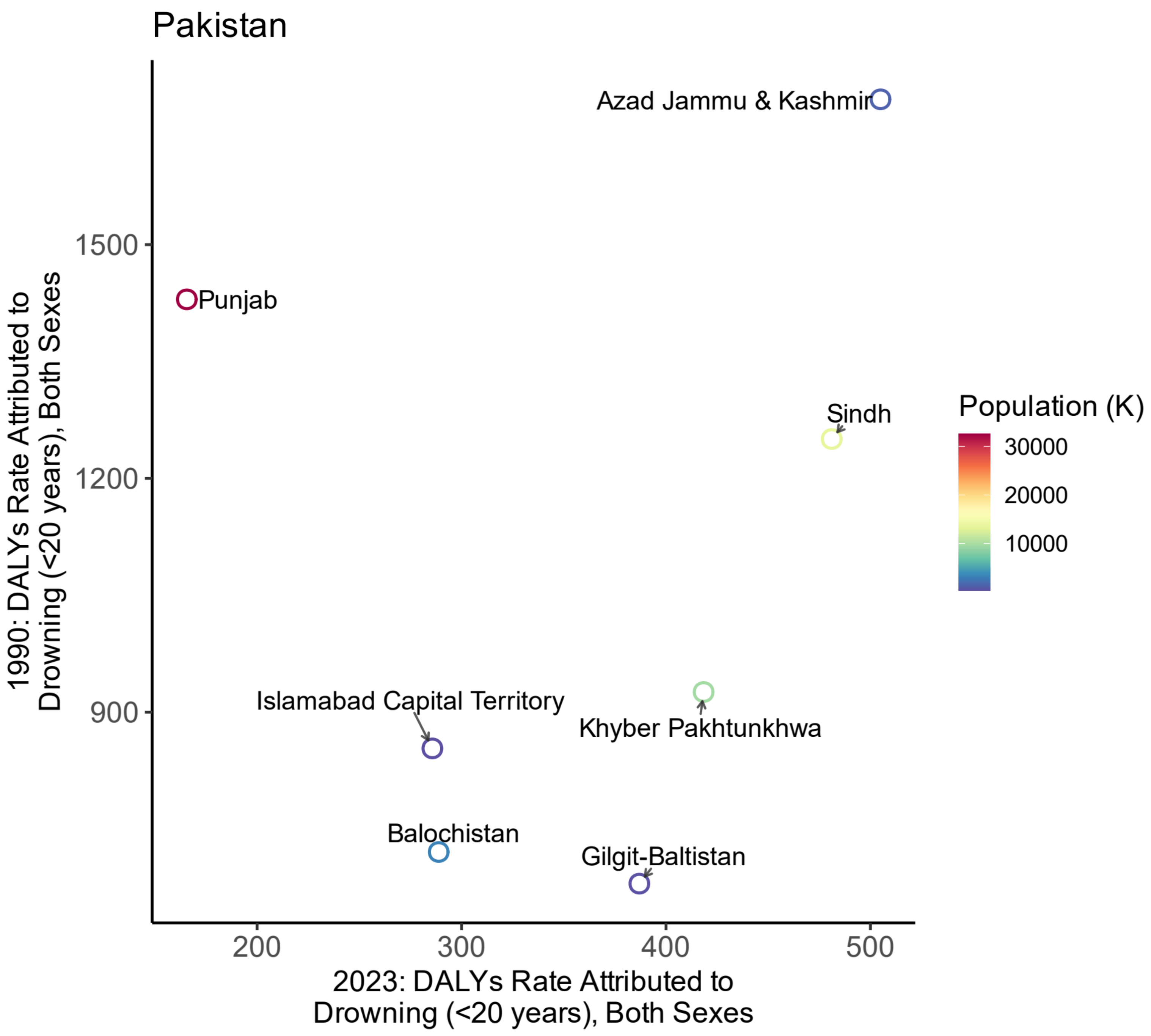

Pakistan
Azad Jammu & Kashmir
Punjab
Sindh
Islamabad Capital Territory
Khyber Pakhtunkhwa
Balochistan
Gilgit-Baltistan
1990: DALYs Rate Attributed to Drowning (<20 years), Both Sexes
2023: DALYs Rate Attributed to Drowning (<20 years), Both Sexes
Population (K)
30000
20000
10000

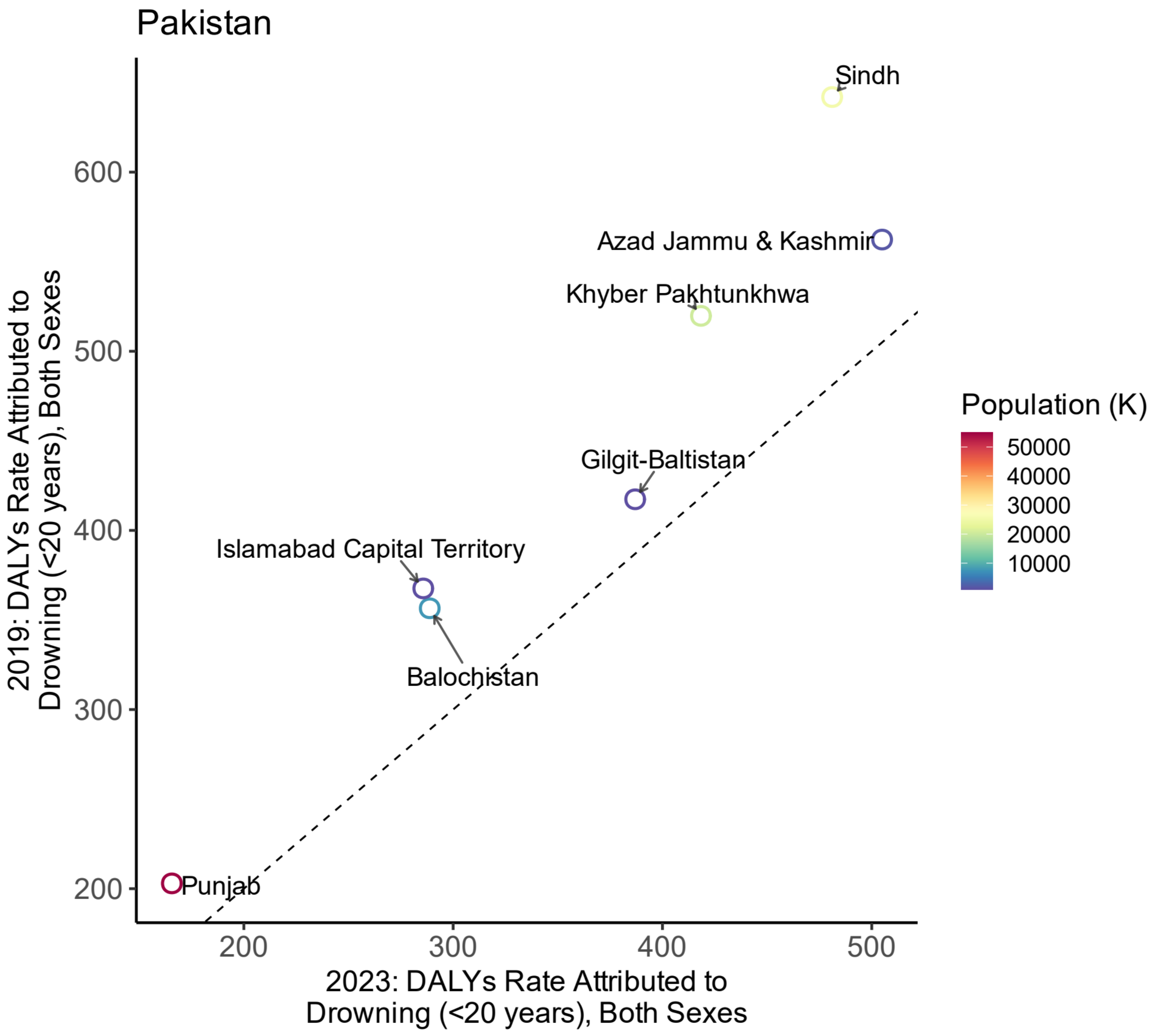

Pakistan
Sindh
Azad Jammu & Kashmir
Khyber Pakhtunkhwa
Gilgit-Baltistan
Islamabad Capital Territory
Balochistan
Punjab
2019: DALYs Rate Attributed to Drowning (<20 years), Both Sexes
2023: DALYs Rate Attributed to Drowning (<20 years), Both Sexes
Population (K)
50000
40000
30000
20000
10000

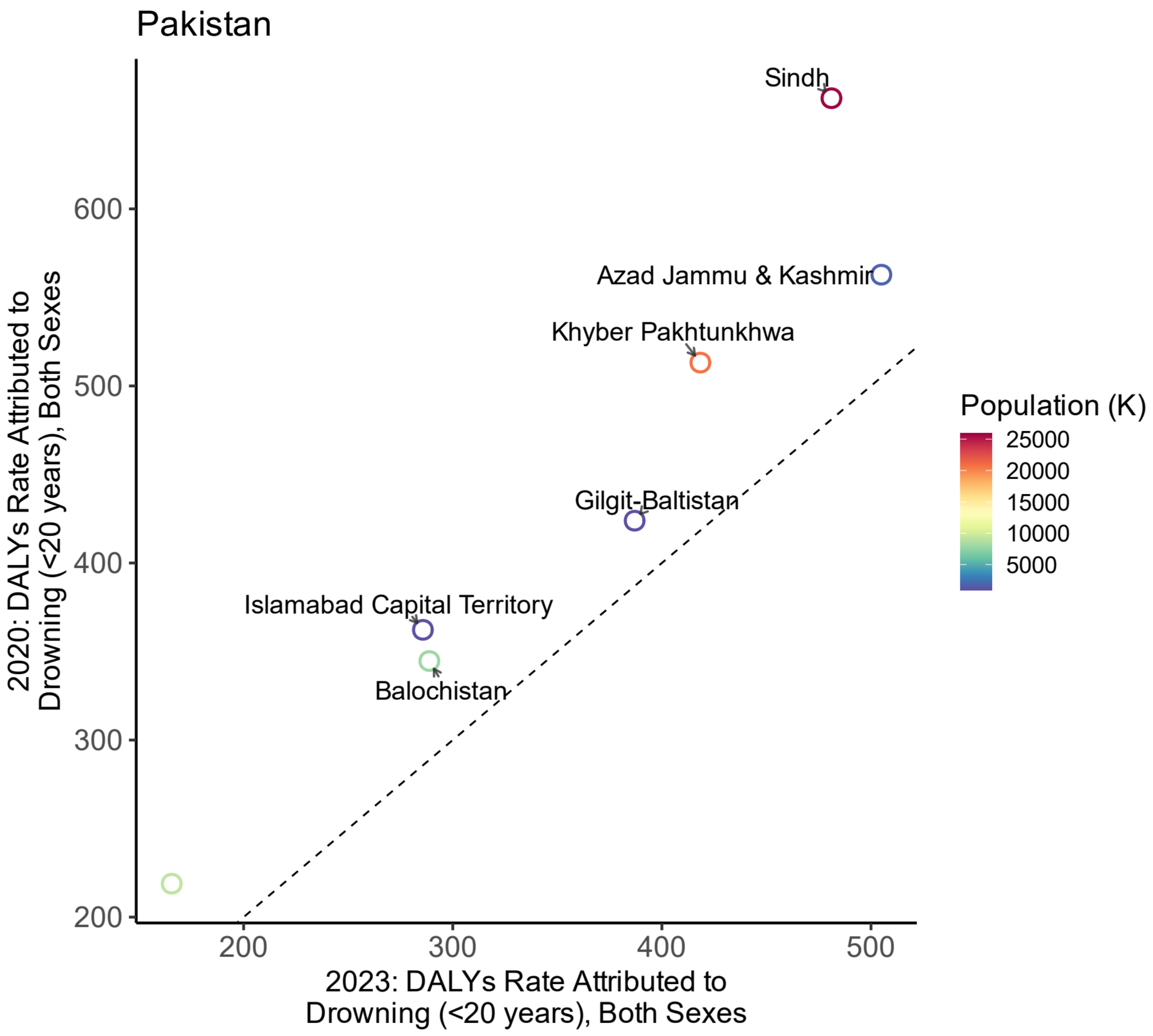

Pakistan
Sindh
Azad Jammu & Kashmir
Khyber Pakhtunkhwa
Gilgit-Baltistan
Islamabad Capital Territory
Balochistan
2020: DALYs Rate Attributed to Drowning (<20 years), Both Sexes
2023: DALYs Rate Attributed to Drowning (<20 years), Both Sexes
Population (K)
25000
20000
15000
10000
5000

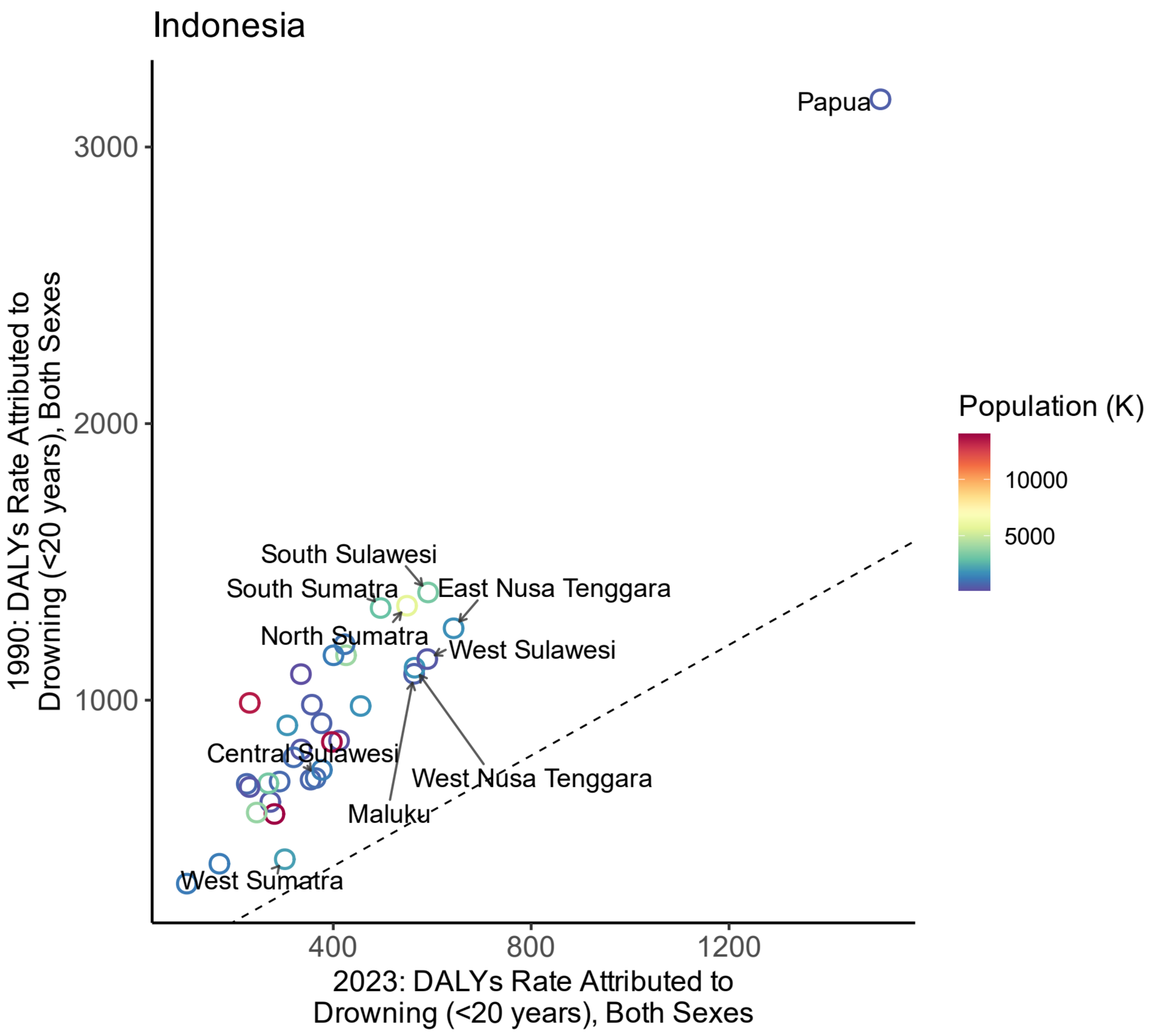
Indonesia
Papua
South Sulawesi
South Sumatra
East Nusa Tenggara
North Sumatra
West Sulawesi
Central Sulawesi
West Nusa Tenggara
Maluku
West Sumatra
3000
2000
1000
400
800
1200
1990: DALYs Rate Attributed to Drowning (<20 years), Both Sexes
2023: DALYs Rate Attributed to Drowning (<20 years), Both Sexes
Population (K)
10000
5000

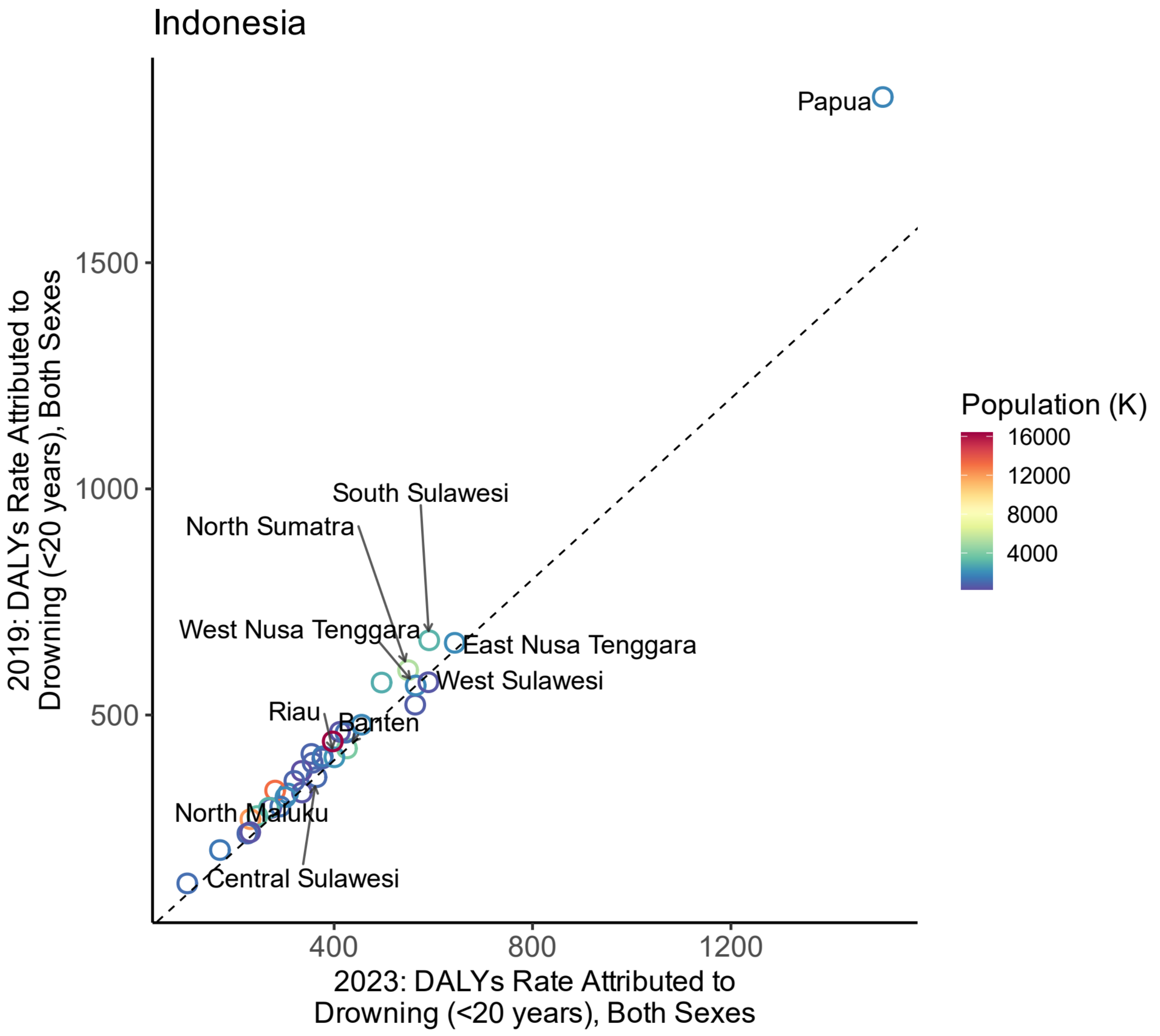
Indonesia
Papua
South Sulawesi
North Sumatra
West Nusa Tenggara
East Nusa Tenggara
West Sulawesi
Riau
Banten
North Maluku
Central Sulawesi
1500
1000
500
400
800
1200
2019: DALYs Rate Attributed to Drowning (<20 years), Both Sexes
2023: DALYs Rate Attributed to Drowning (<20 years), Both Sexes
Population (K)
16000
12000
8000
4000

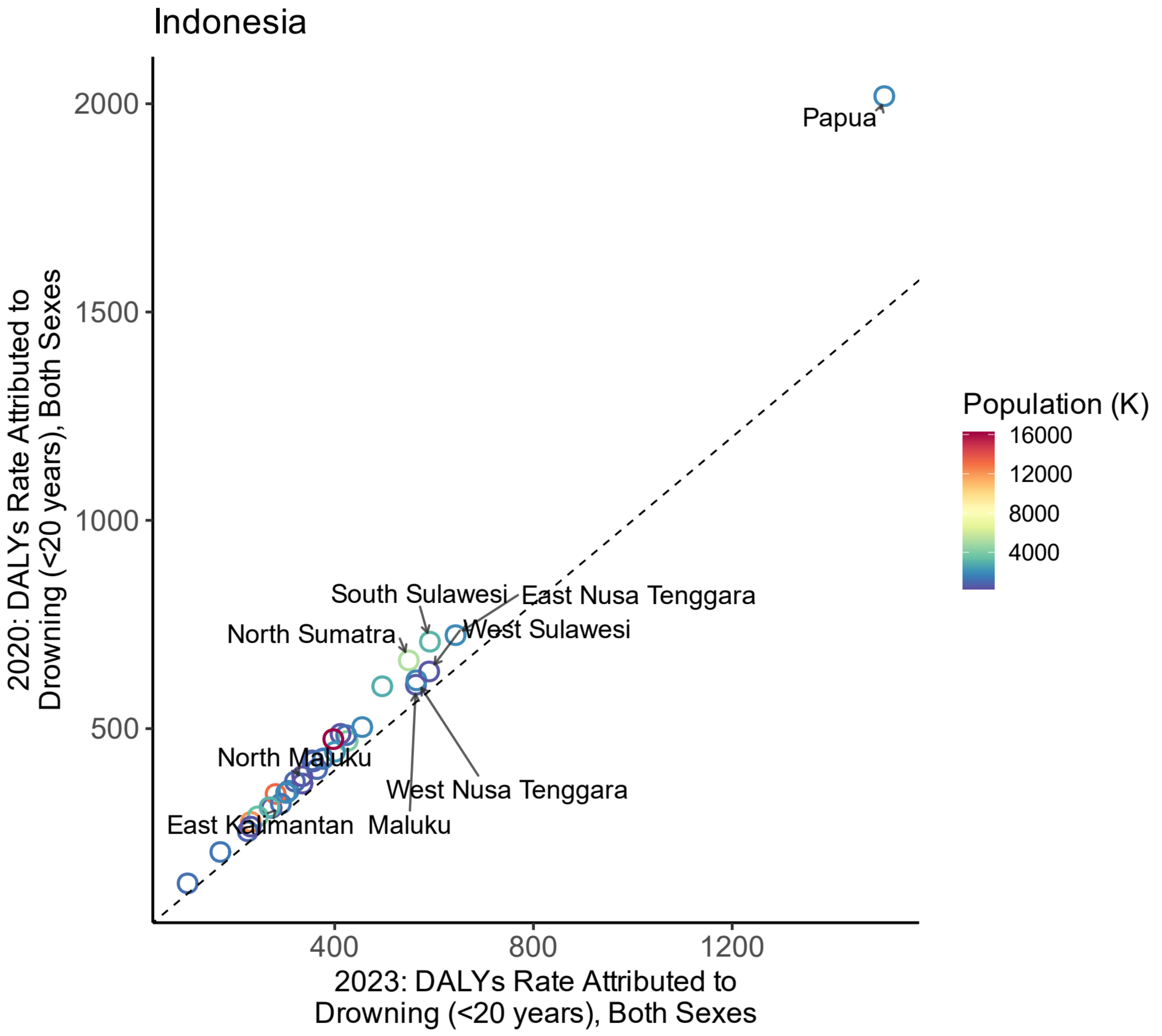
Indonesia
Papua
South Sulawesi
East Nusa Tenggara
North Sumatra
West Sulawesi
North Maluku
West Nusa Tenggara
East Kalimantan
Maluku
2000
1500
1000
500
400
800
1200
2020: DALYs Rate Attributed to Drowning (<20 years), Both Sexes
2023: DALYs Rate Attributed to Drowning (<20 years), Both Sexes
Population (K)
16000
12000
8000
4000

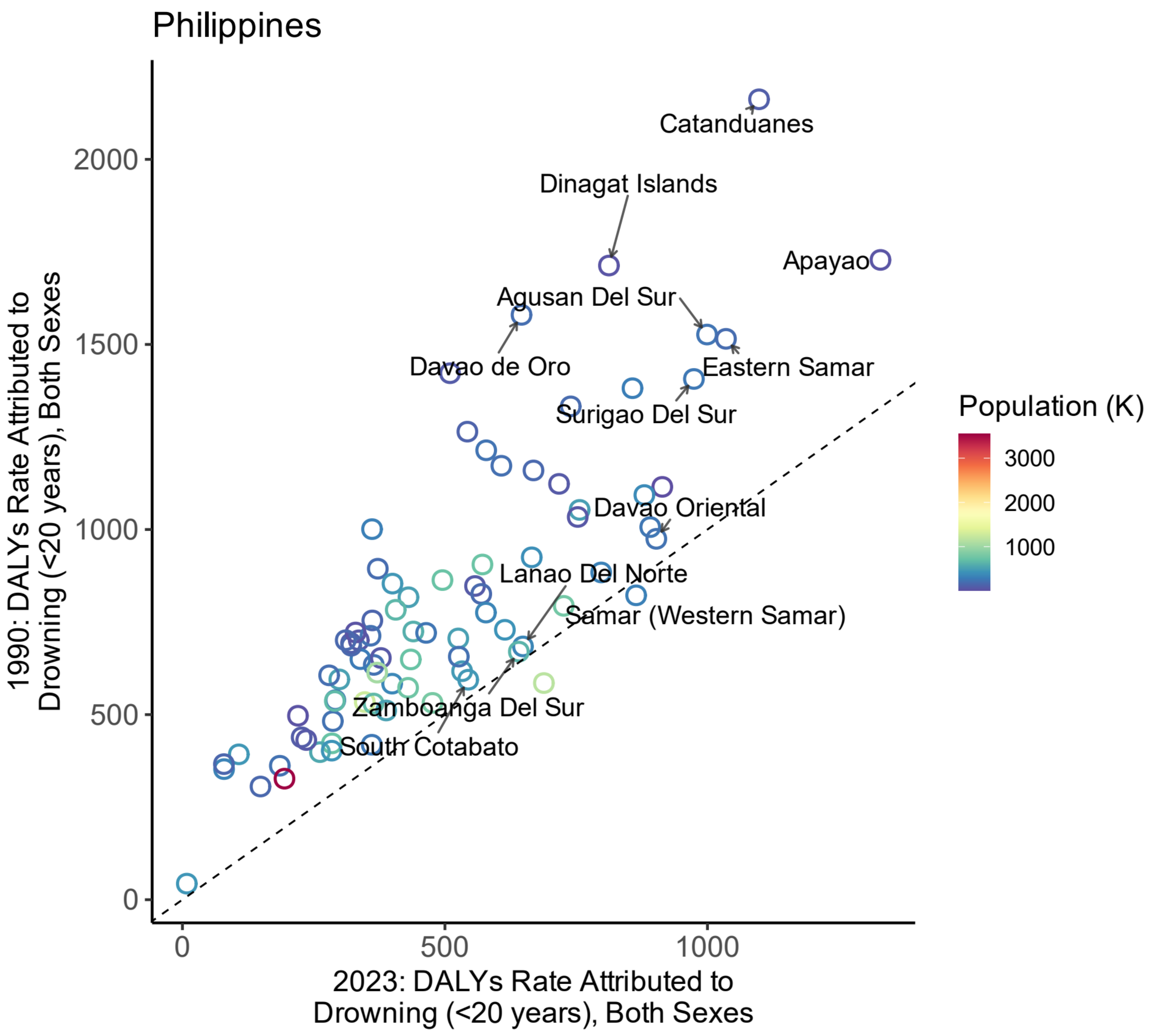
Philippines
Catanduanes
Dinagat Islands
Apayao
Agusan Del Sur
Davao de Oro
Eastern Samar
Surigao Del Sur
Davao Oriental
Lanao Del Norte
Samar (Western Samar)
Zamboanga Del Sur
South Cotabato
1990: DALYs Rate Attributed to Drowning (<20 years), Both Sexes
2023: DALYs Rate Attributed to Drowning (<20 years), Both Sexes
Population (K)
0
500
1000
1500
2000
3000

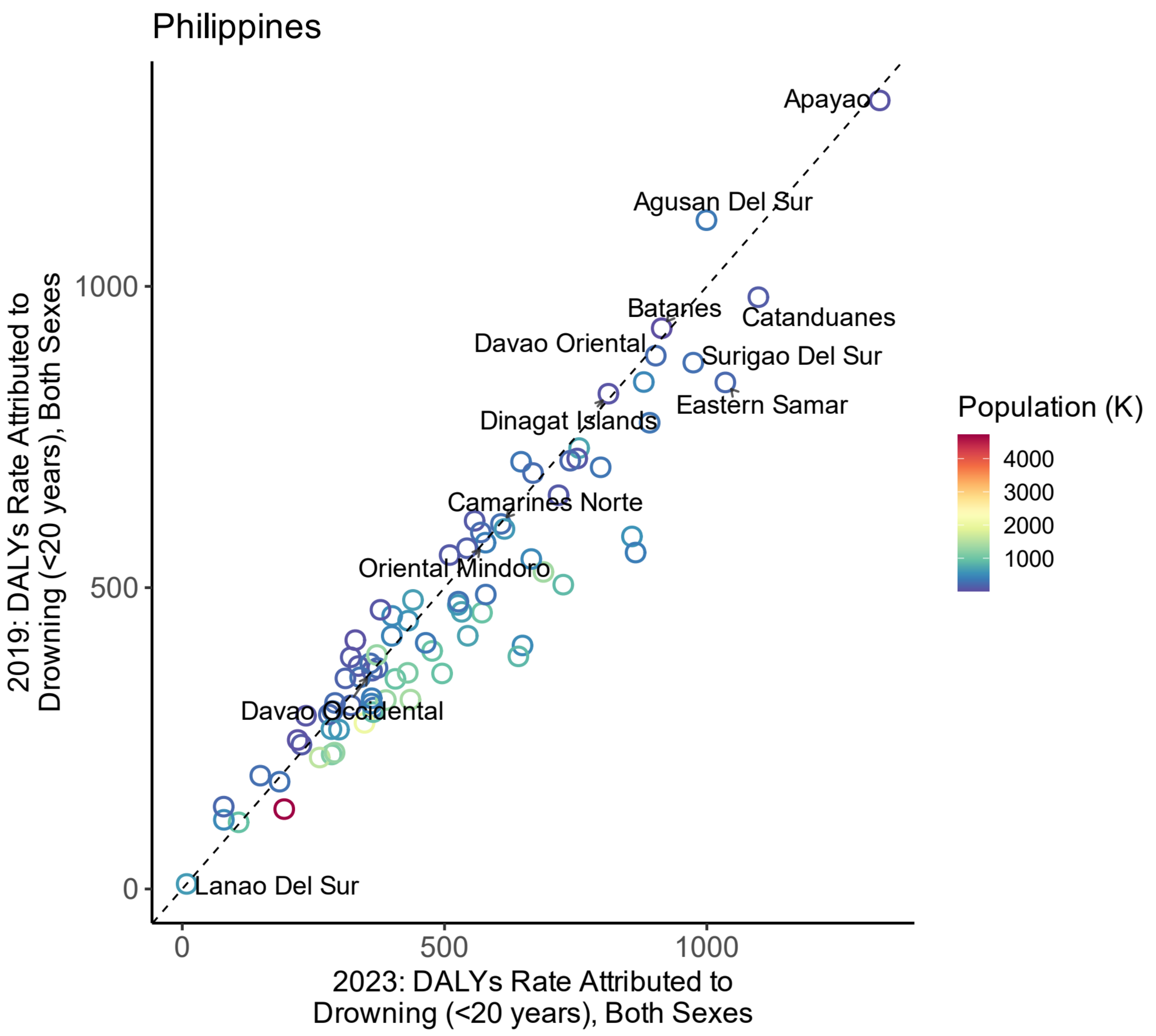
Philippines
Apayao
Agusan Del Sur
Batanes
Catanduanes
Davao Oriental
Surigao Del Sur
Eastern Samar
Dinagat Islands
Camarines Norte
Oriental Mindoro
Davao Occidental
Lanao Del Sur
2019: DALYs Rate Attributed to Drowning (<20 years), Both Sexes
2023: DALYs Rate Attributed to Drowning (<20 years), Both Sexes
Population (K)
0
500
1000
2000
3000
4000

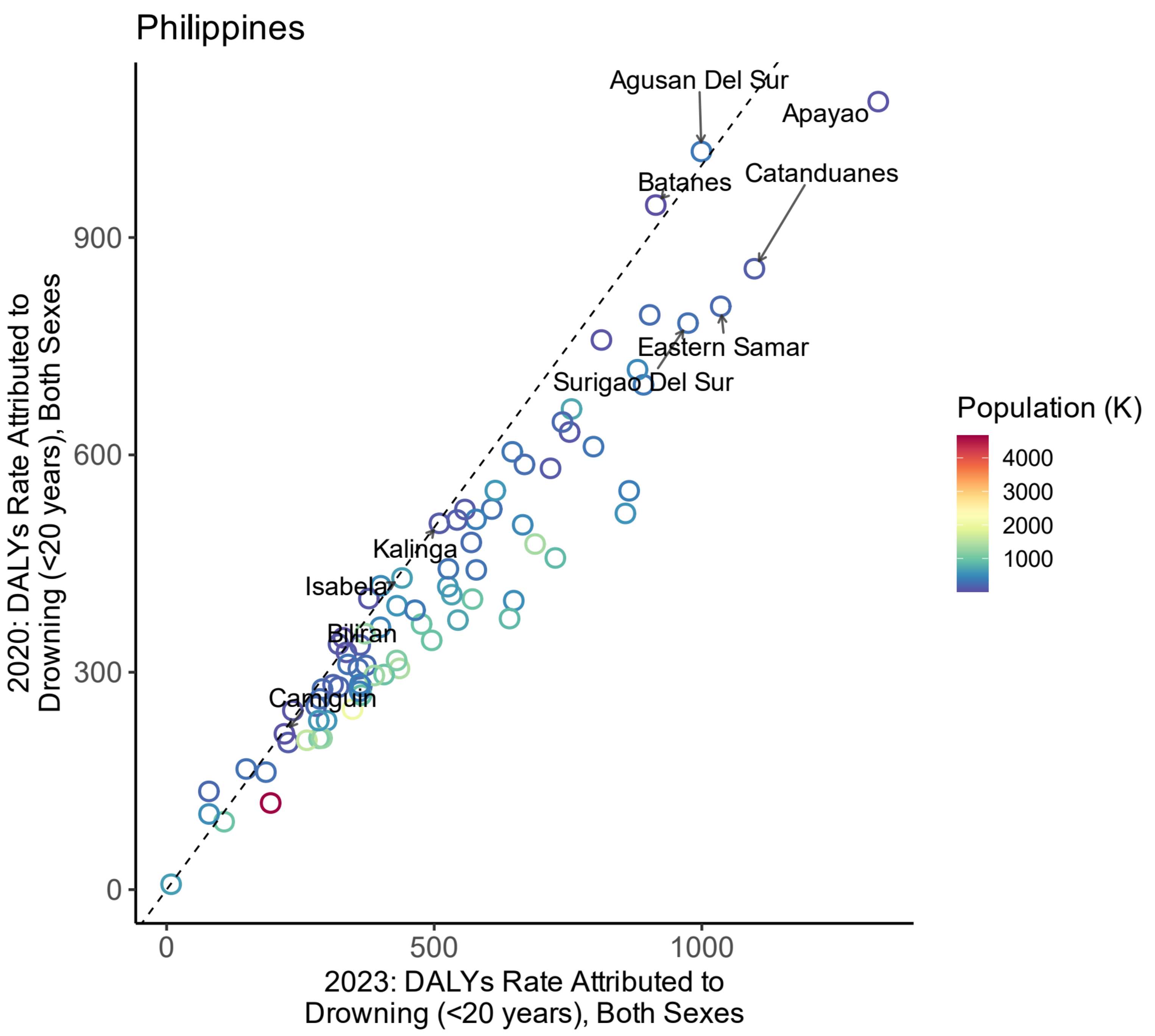
Philippines
Agusan Del Sur
Apayao
Batanes
Catanduanes
Eastern Samar
Surigao Del Sur
Kalinga
Isabela
Biliran
Camiguin
2020: DALYs Rate Attributed to Drowning (<20 years), Both Sexes
2023: DALYs Rate Attributed to Drowning (<20 years), Both Sexes
Population (K)
0
300
600
900
500
1000
2000
3000
4000

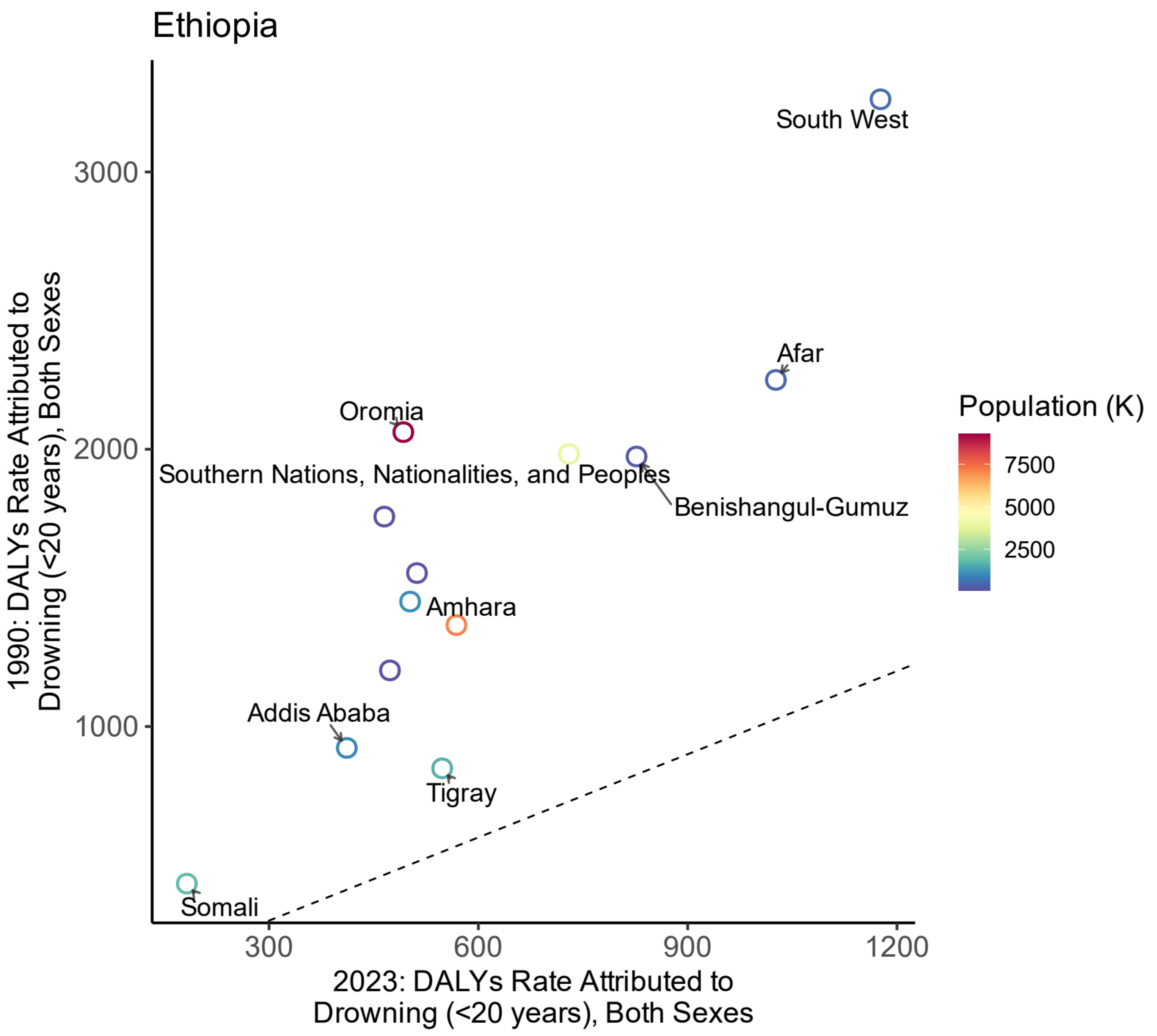
Ethiopia
1990: DALYs Rate Attributed to Drowning (<20 years), Both Sexes
2023: DALYs Rate Attributed to Drowning (<20 years), Both Sexes
Population (K)
7500
5000
2500
3000
2000
1000
300
600
900
1200
South West
Afar
Oromia
Southern Nations, Nationalities, and Peoples
Benishangul-Gumuz
Amhara
Addis Ababa
Tigray
Somali

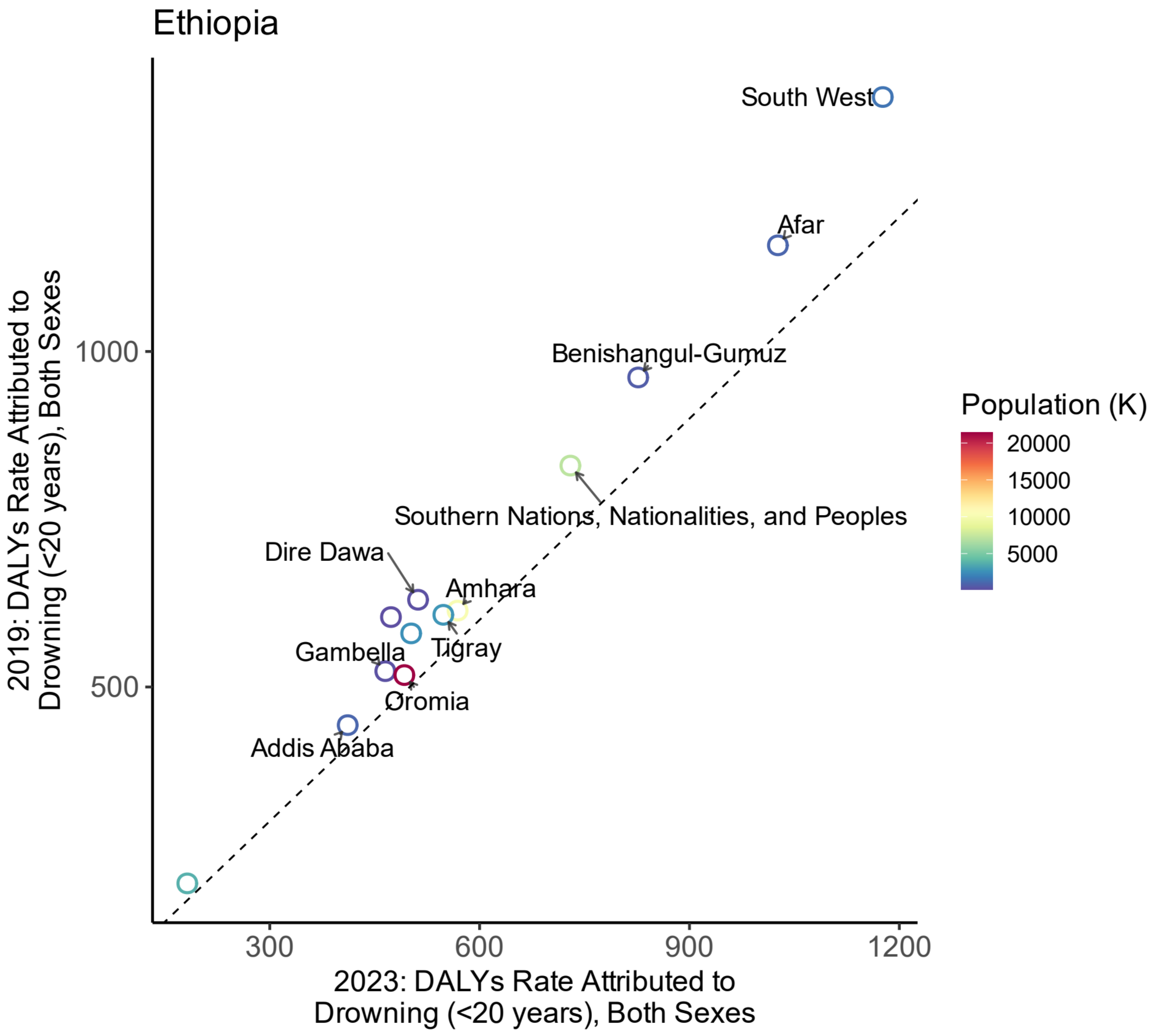
Ethiopia
2019: DALYs Rate Attributed to Drowning (<20 years), Both Sexes
2023: DALYs Rate Attributed to Drowning (<20 years), Both Sexes
Population (K)
20000
15000
10000
5000
1000
500
300
600
900
1200
South West
Afar
Benishangul-Gumuz
Southern Nations, Nationalities, and Peoples
Dire Dawa
Amhara
Gambella
Tigray
Oromia
Addis Ababa

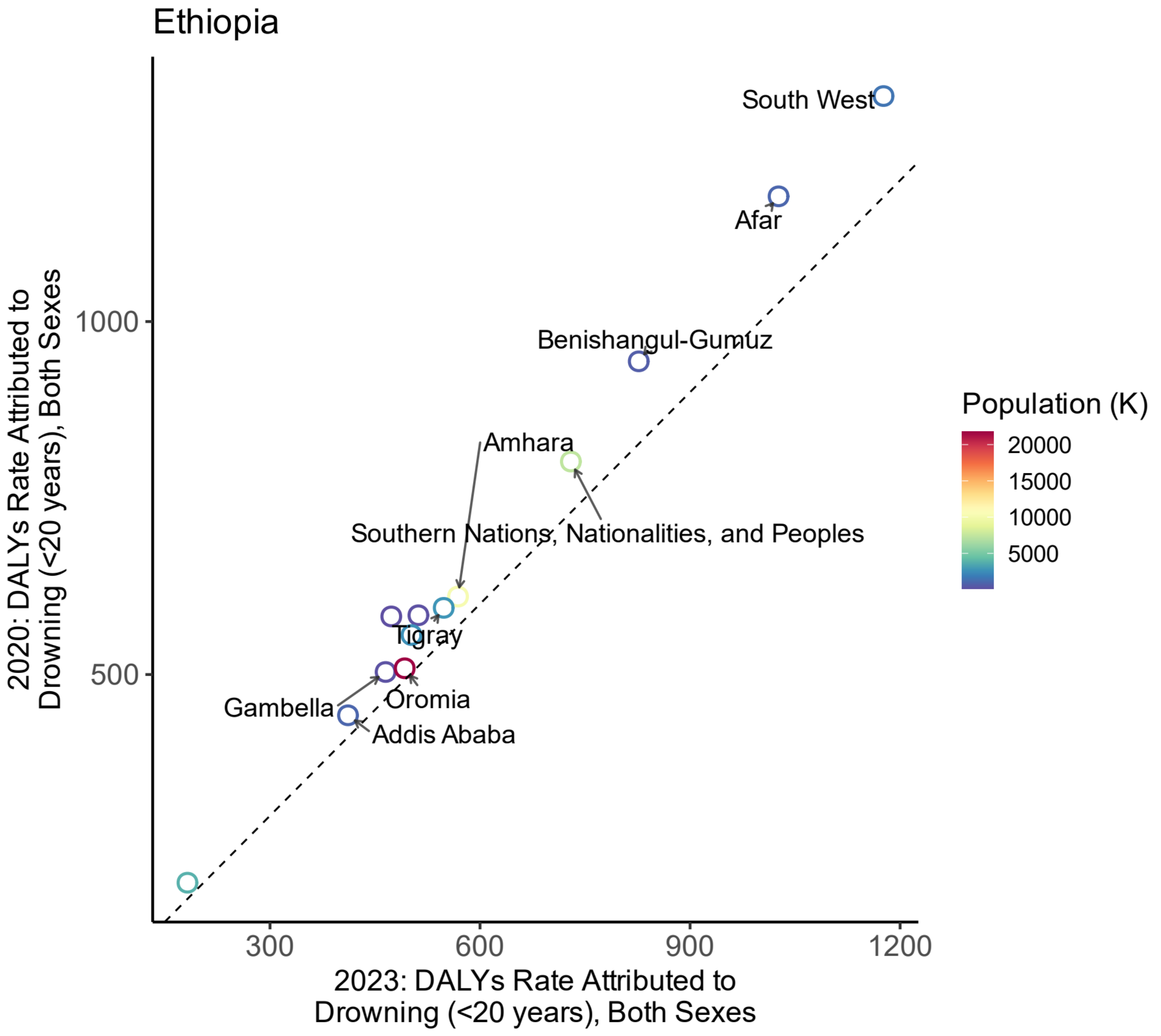
Ethiopia
2020: DALYs Rate Attributed to Drowning (<20 years), Both Sexes
2023: DALYs Rate Attributed to Drowning (<20 years), Both Sexes
Population (K)
20000
15000
10000
5000
1000
500
300
600
900
1200
South West
Afar
Benishangul-Gumuz
Amhara
Southern Nations, Nationalities, and Peoples
Tigray
Gambella
Oromia
Addis Ababa

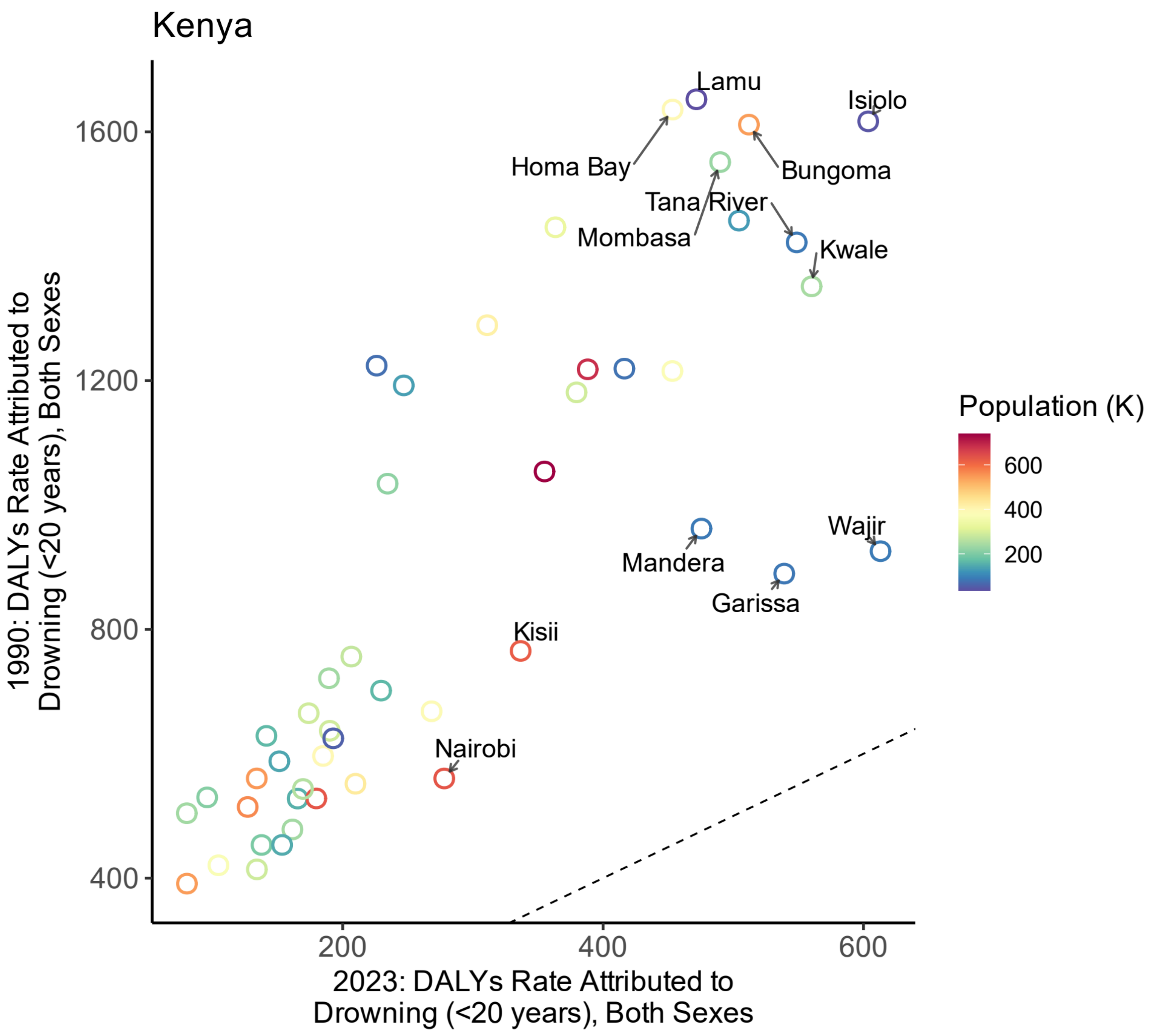
Kenya
1990: DALYs Rate Attributed to Drowning (<20 years), Both Sexes
2023: DALYs Rate Attributed to Drowning (<20 years), Both Sexes
Population (K)
Lamu
Isiolo
Homa Bay
Bungoma
Tana River
Mombasa
Kwale
Mandera
Wajir
Garissa
Kisii
Nairobi

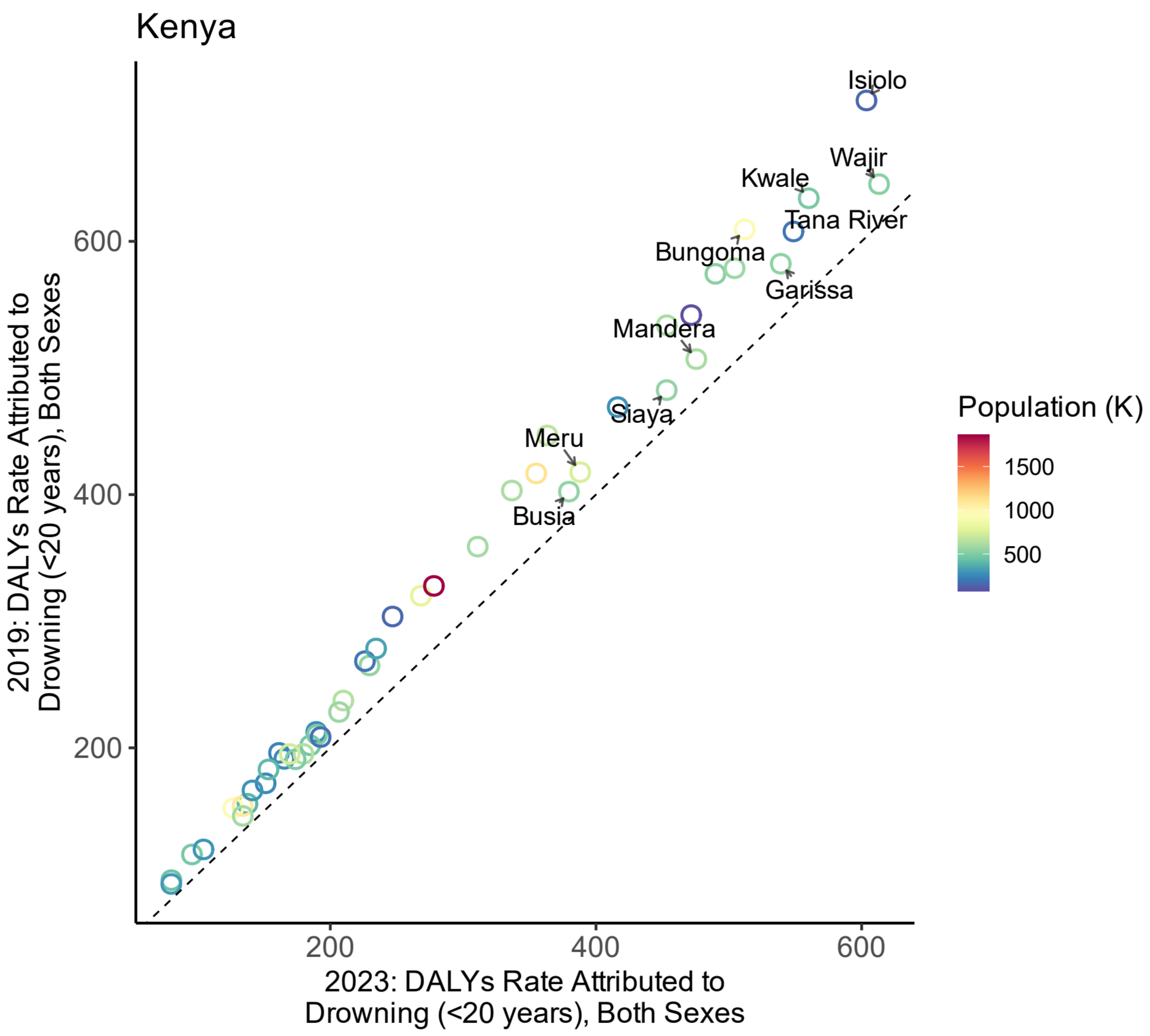
Kenya
2019: DALYs Rate Attributed to Drowning (<20 years), Both Sexes
2023: DALYs Rate Attributed to Drowning (<20 years), Both Sexes
Population (K)
Isiolo
Wajir
Kwale
Tana River
Bungoma
Garissa
Mandera
Siaya
Meru
Busia

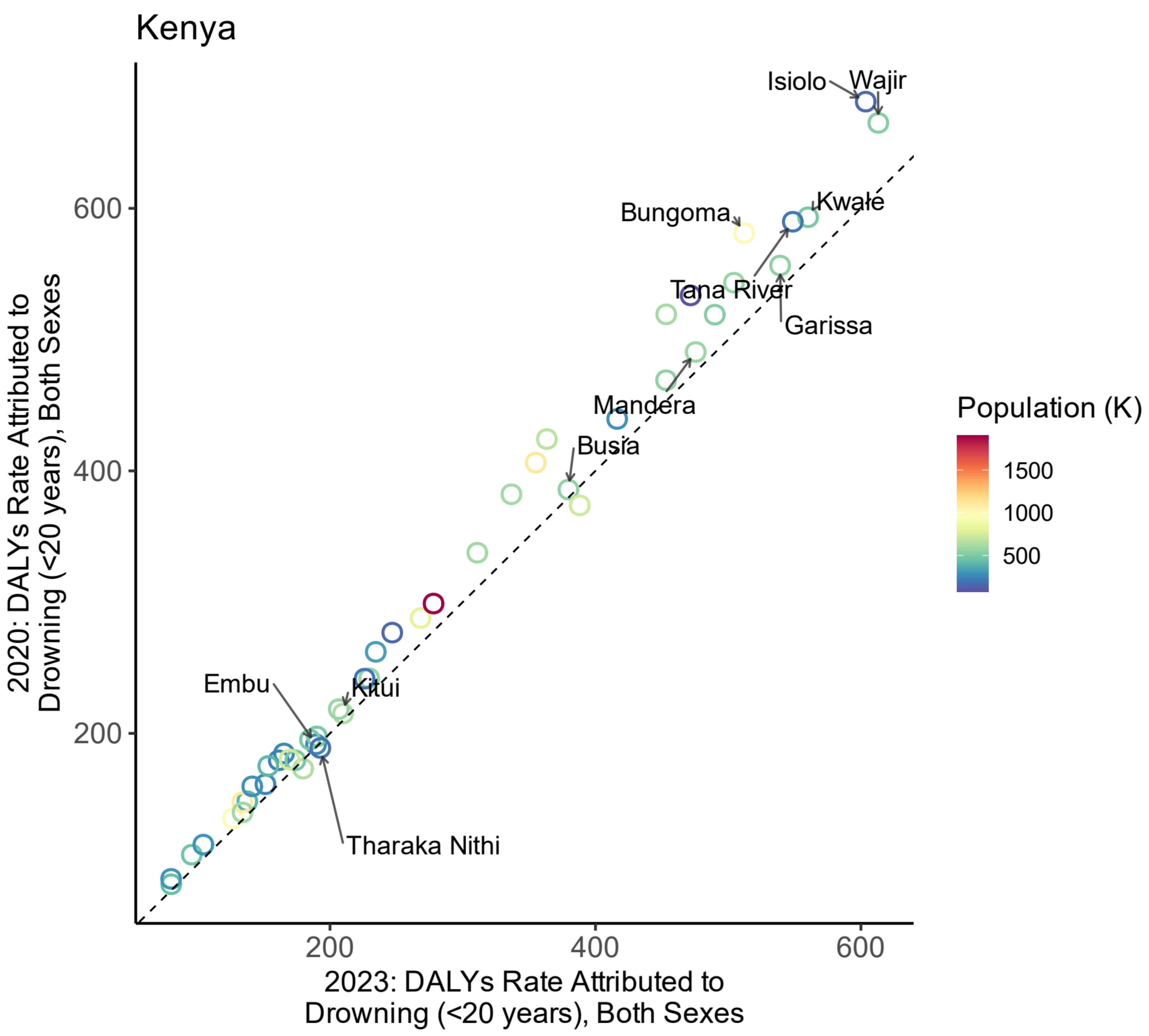
Kenya
2020: DALYs Rate Attributed to Drowning (<20 years), Both Sexes
2023: DALYs Rate Attributed to Drowning (<20 years), Both Sexes
Population (K)
Isiolo
Wajir
Bungoma
Kwale
Tana River
Garissa
Mandera
Busia
Embu
Kitui
Tharaka Nithi

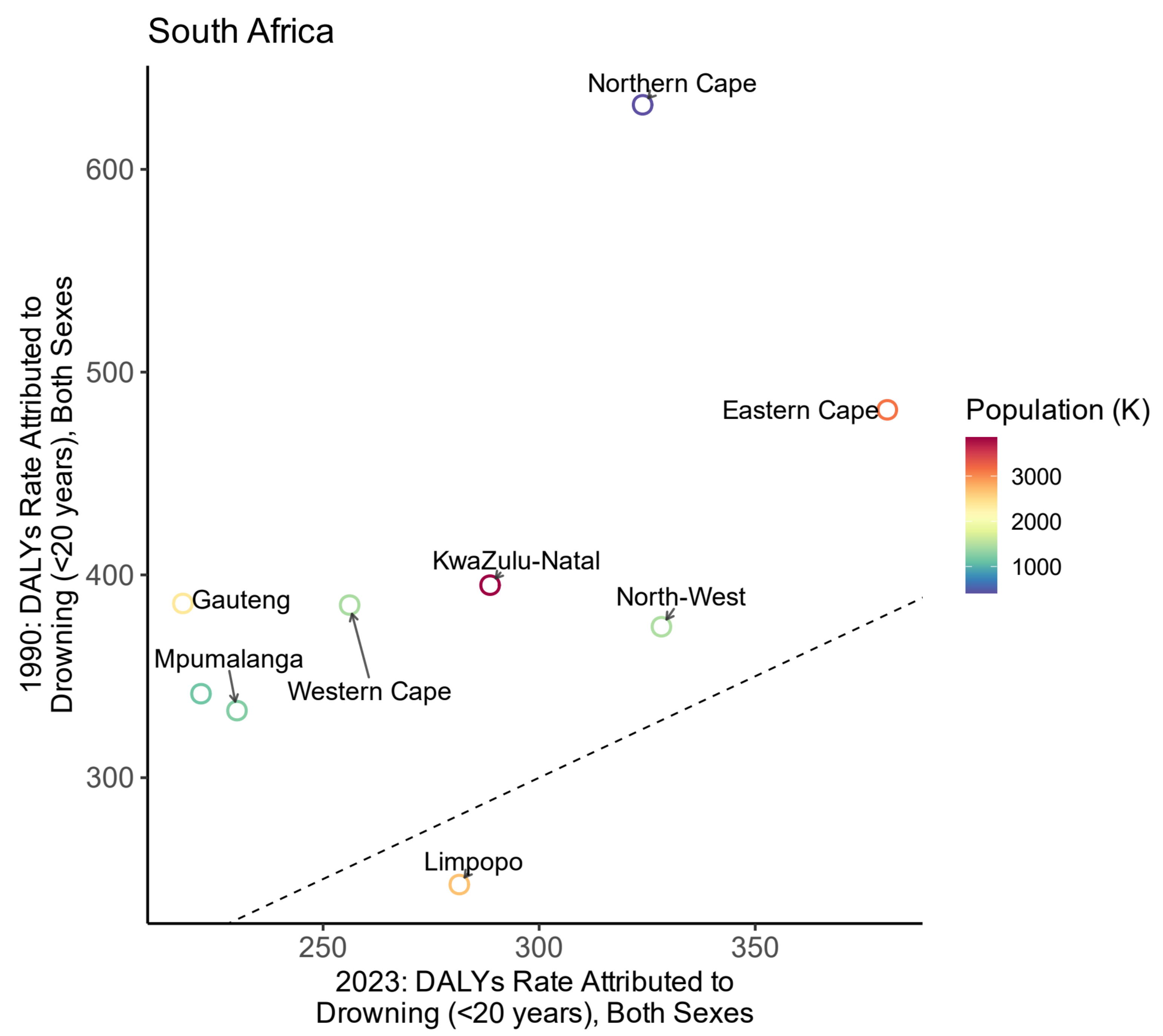
South Africa
1990: DALYs Rate Attributed to Drowning (<20 years), Both Sexes
2023: DALYs Rate Attributed to Drowning (<20 years), Both Sexes
Northern Cape
Eastern Cape
KwaZulu-Natal
North-West
Gauteng
Mpumalanga
Western Cape
Limpopo
Population (K)

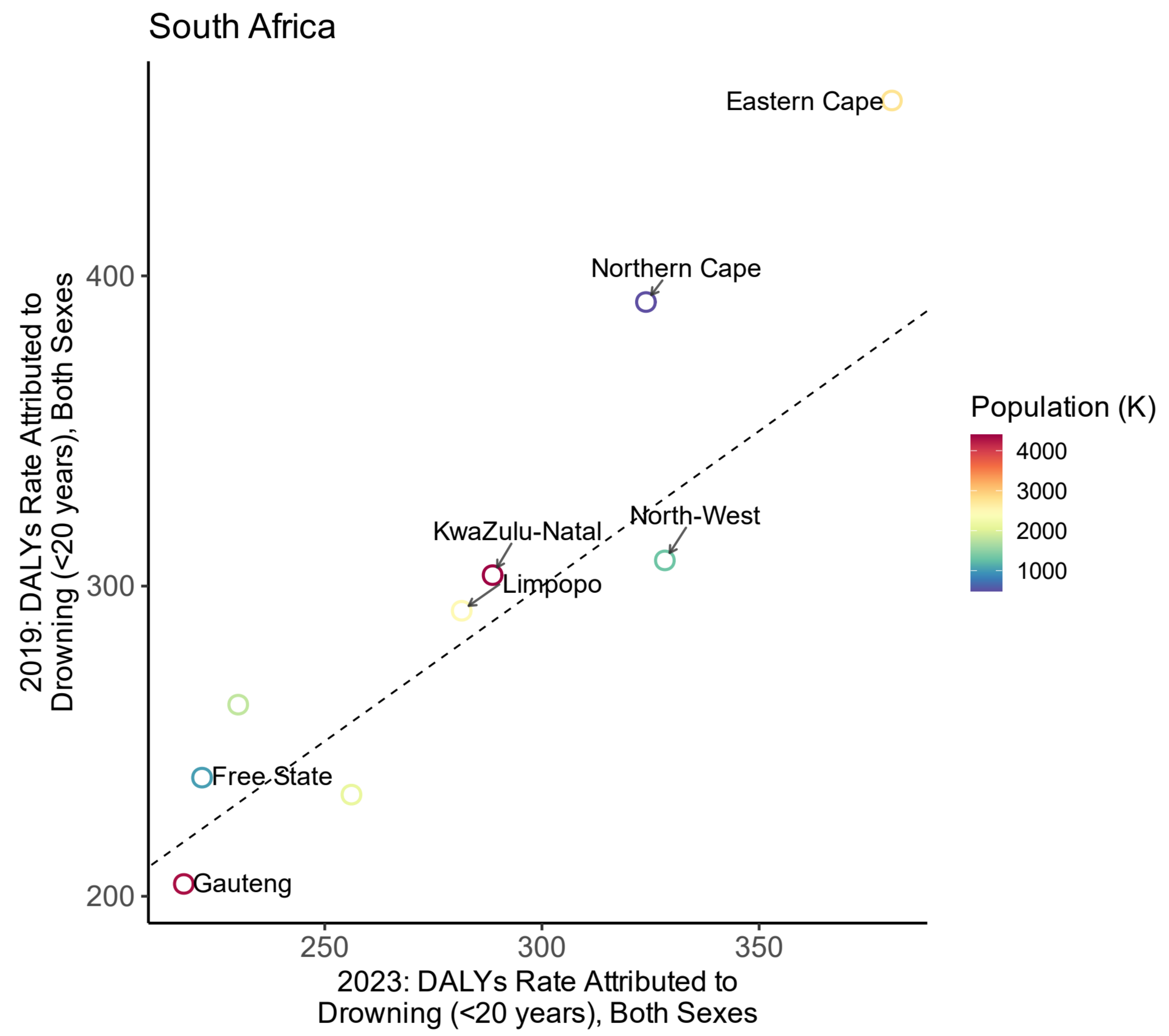
South Africa
2019: DALYs Rate Attributed to Drowning (<20 years), Both Sexes
2023: DALYs Rate Attributed to Drowning (<20 years), Both Sexes
Eastern Cape
Northern Cape
KwaZulu-Natal
North-West
Limpopo
Free State
Gauteng
Population (K)

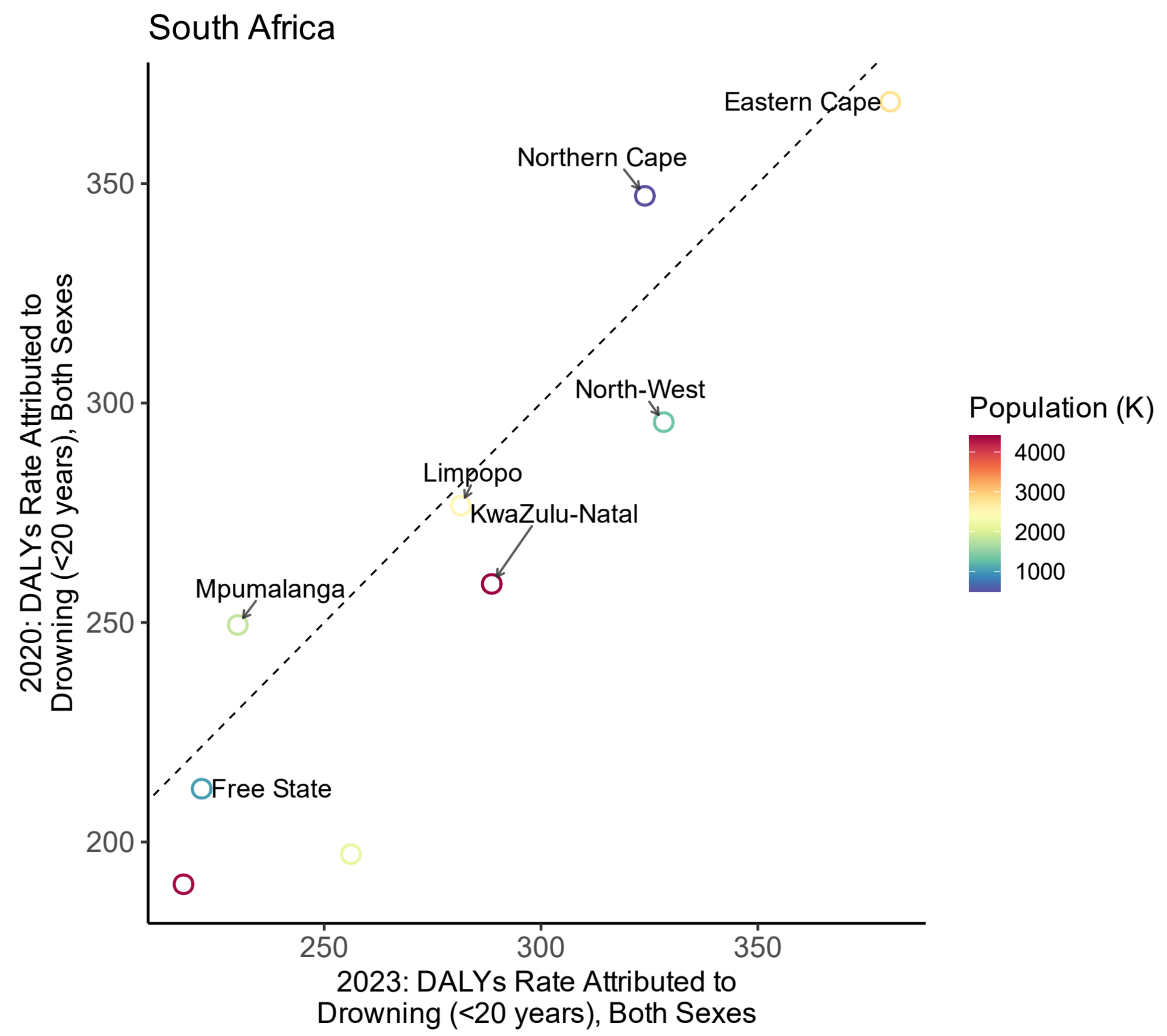
South Africa
2020: DALYs Rate Attributed to Drowning (<20 years), Both Sexes
2023: DALYs Rate Attributed to Drowning (<20 years), Both Sexes
Eastern Cape
Northern Cape
North-West
Limpopo
KwaZulu-Natal
Mpumalanga
Free State
Population (K)

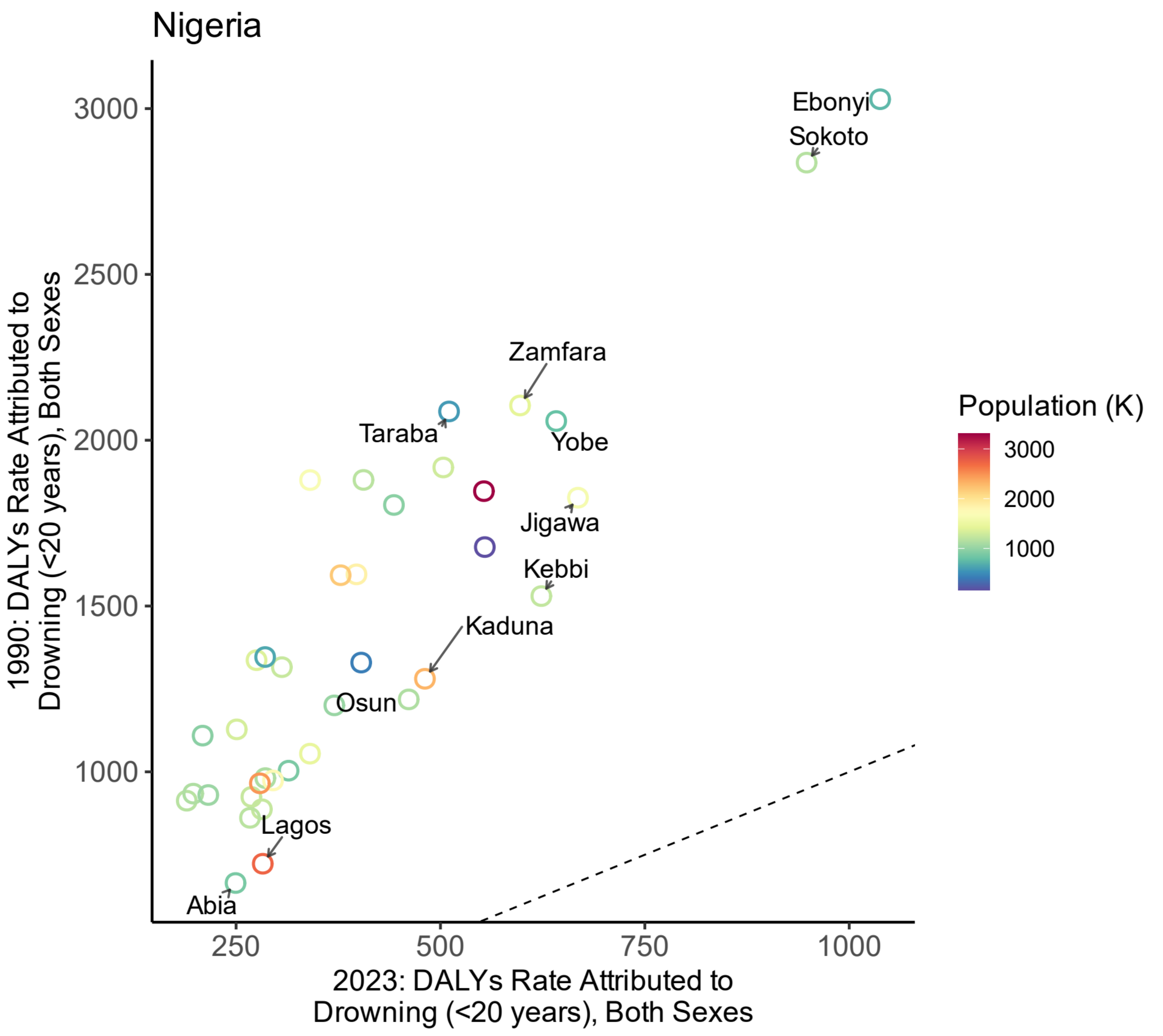
Nigeria
1990: DALYs Rate Attributed to Drowning (<20 years), Both Sexes
2023: DALYs Rate Attributed to Drowning (<20 years), Both Sexes
Population (K)
3000
2000
1000
Ebonyi
Sokoto
Zamfara
Taraba
Yobe
Jigawa
Kebbi
Kaduna
Osun
Lagos
Abia
3000
2500
2000
1500
1000
250
500
750
1000

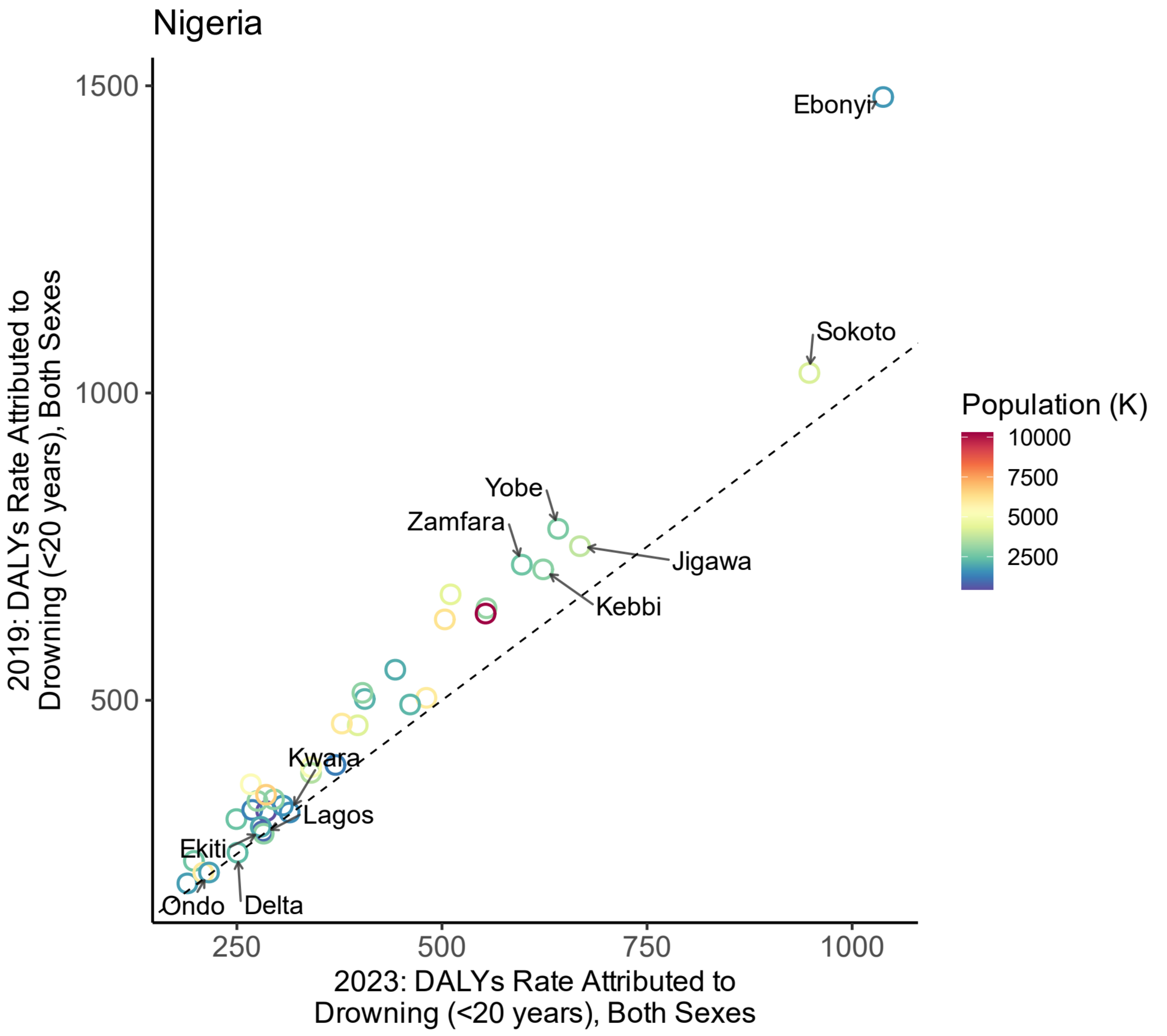
Nigeria
2019: DALYs Rate Attributed to Drowning (<20 years), Both Sexes
2023: DALYs Rate Attributed to Drowning (<20 years), Both Sexes
Population (K)
10000
7500
5000
2500
Ebonyi
Sokoto
Yobe
Zamfara
Jigawa
Kebbi
Kwara
Lagos
Ekiti
Ondo
Delta
1500
1000
500
250
500
750
1000

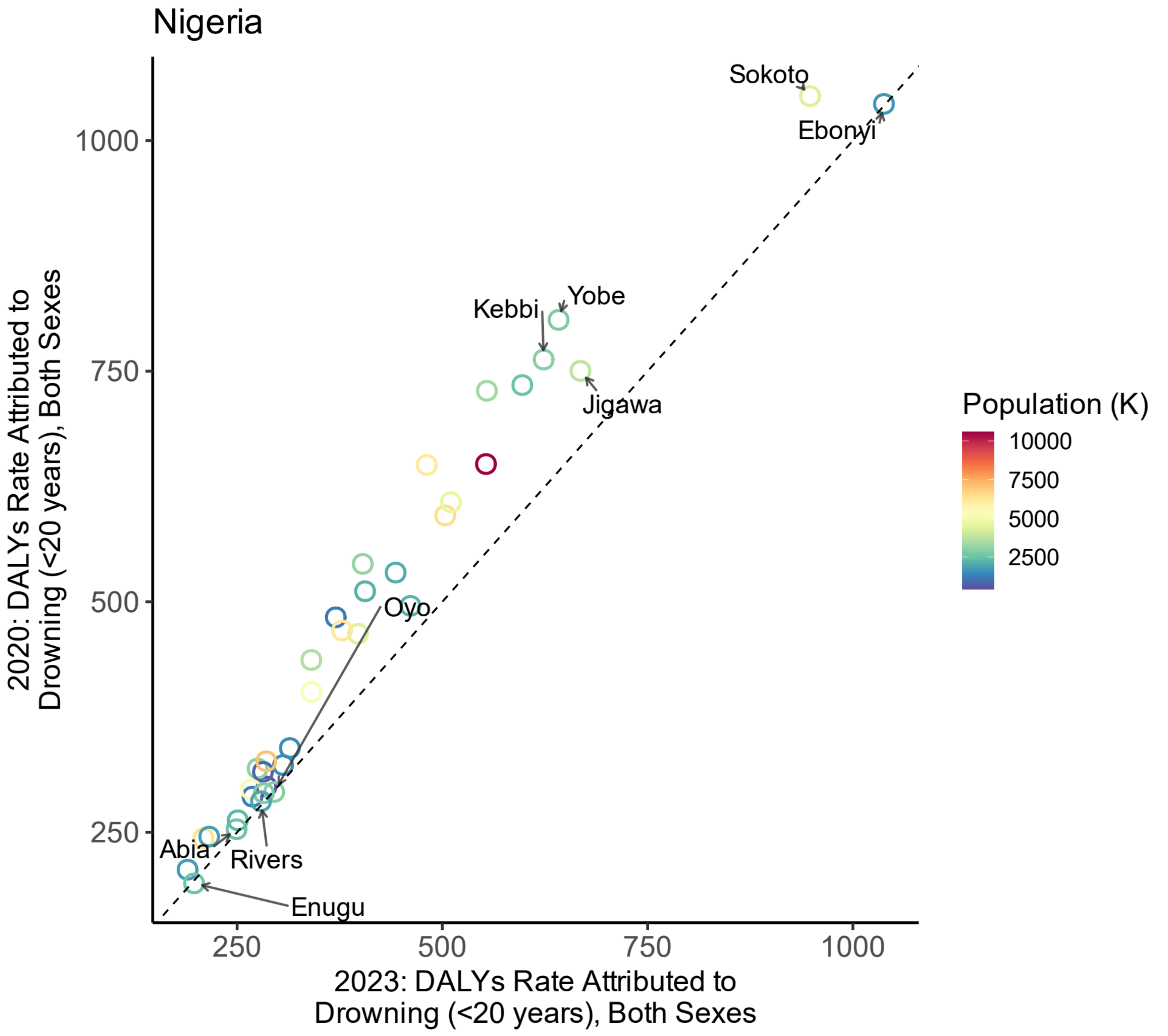
Nigeria
2020: DALYs Rate Attributed to Drowning (<20 years), Both Sexes
2023: DALYs Rate Attributed to Drowning (<20 years), Both Sexes
Population (K)
10000
7500
5000
2500
Sokoto
Ebonyi
Kebbi
Yobe
Jigawa
Oyo
Abia
Rivers
Enugu
1000
750
500
250
250
500
750
1000

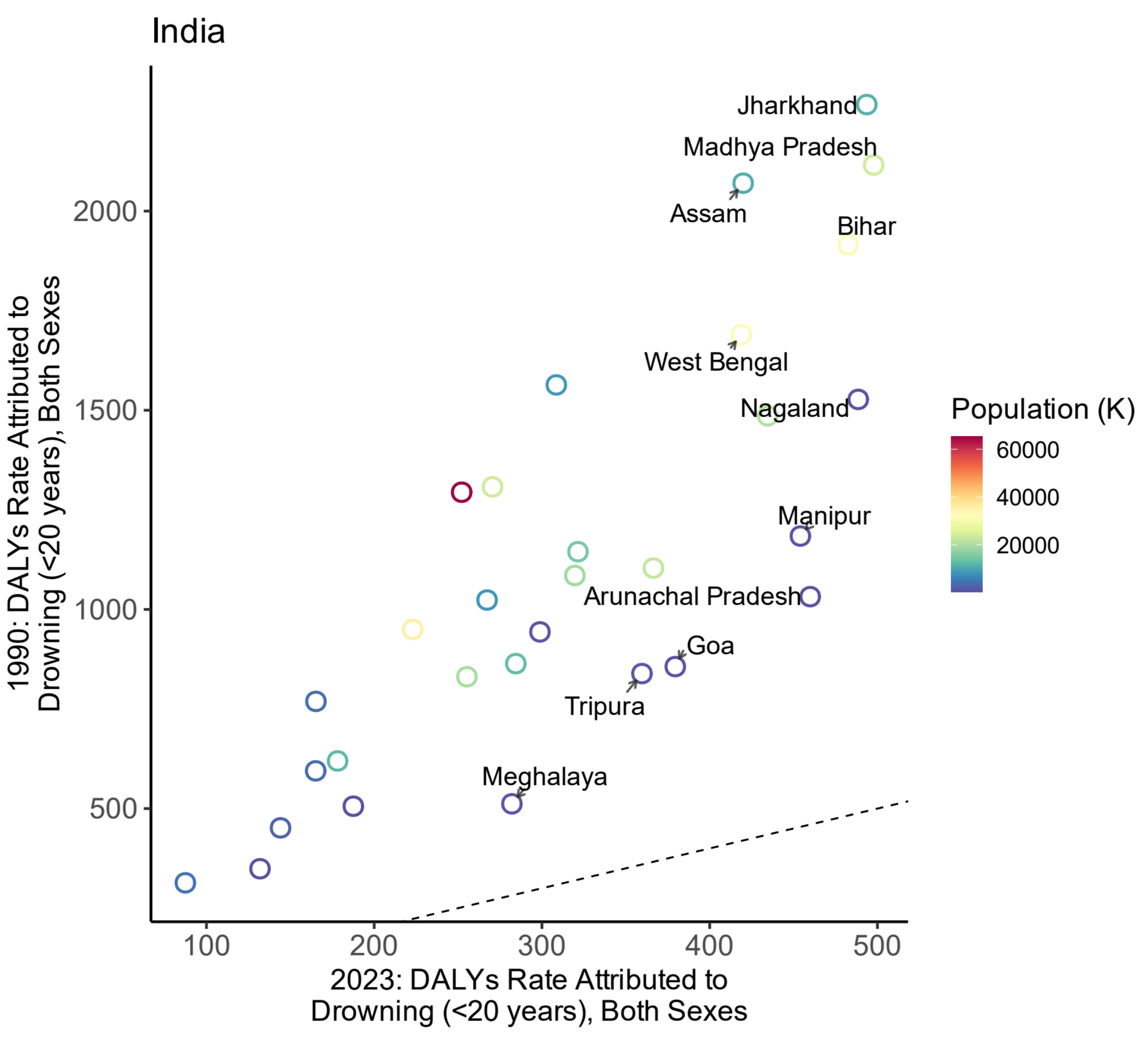
India
Jharkhand
Madhya Pradesh
Assam
Bihar
West Bengal
Nagaland
Manipur
Arunachal Pradesh
Goa
Tripura
Meghalaya
2000
1500
1000
500
100
200
300
400
500
1990: DALYs Rate Attributed to Drowning (<20 years), Both Sexes
2023: DALYs Rate Attributed to Drowning (<20 years), Both Sexes
Population (K)
60000
40000
20000

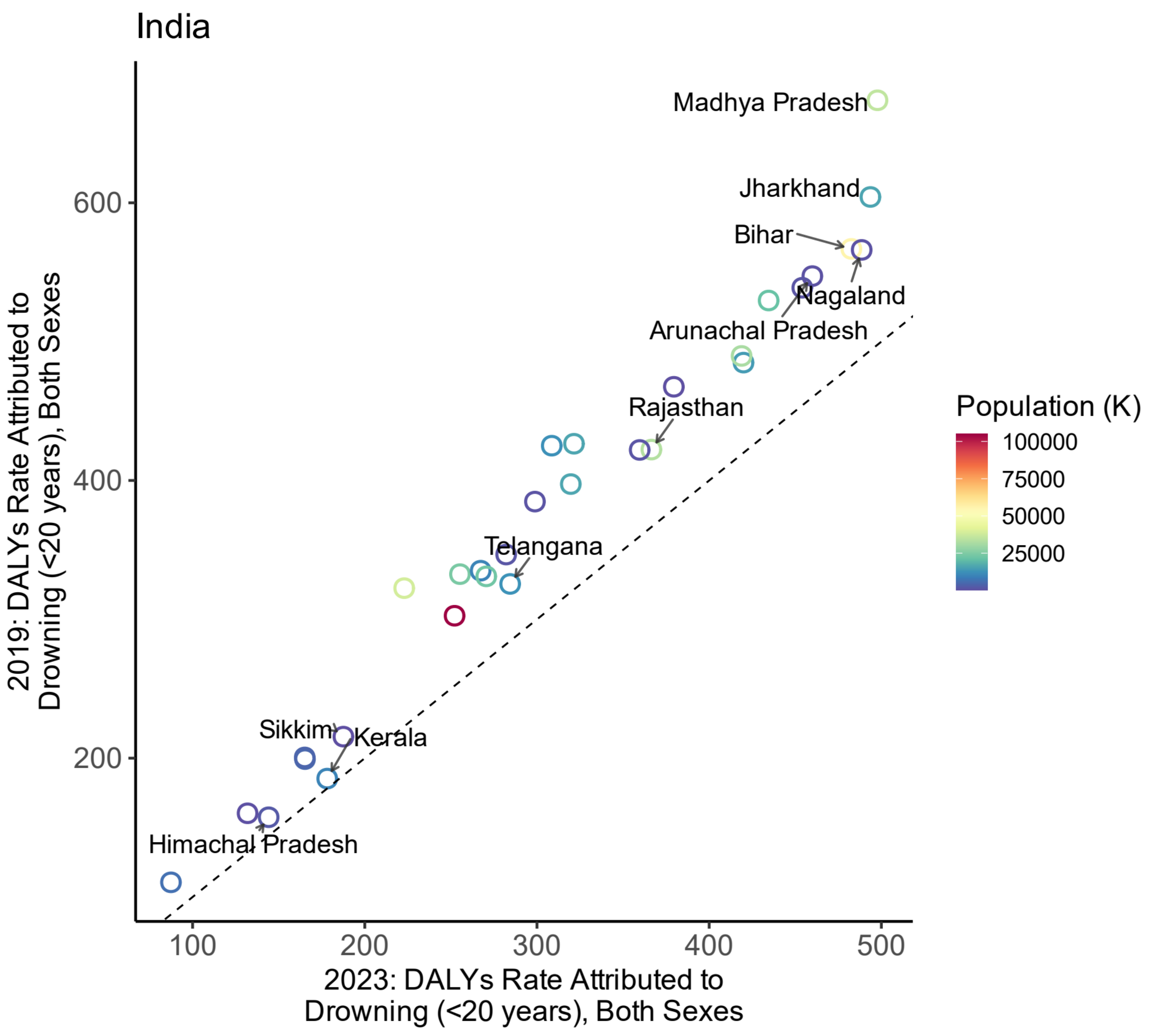
India
Madhya Pradesh
Jharkhand
Bihar
Nagaland
Arunachal Pradesh
Rajasthan
Telangana
Sikkim
Kerala
Himachal Pradesh
600
400
200
100
200
300
400
500
2019: DALYs Rate Attributed to Drowning (<20 years), Both Sexes
2023: DALYs Rate Attributed to Drowning (<20 years), Both Sexes
Population (K)
100000
75000
50000
25000

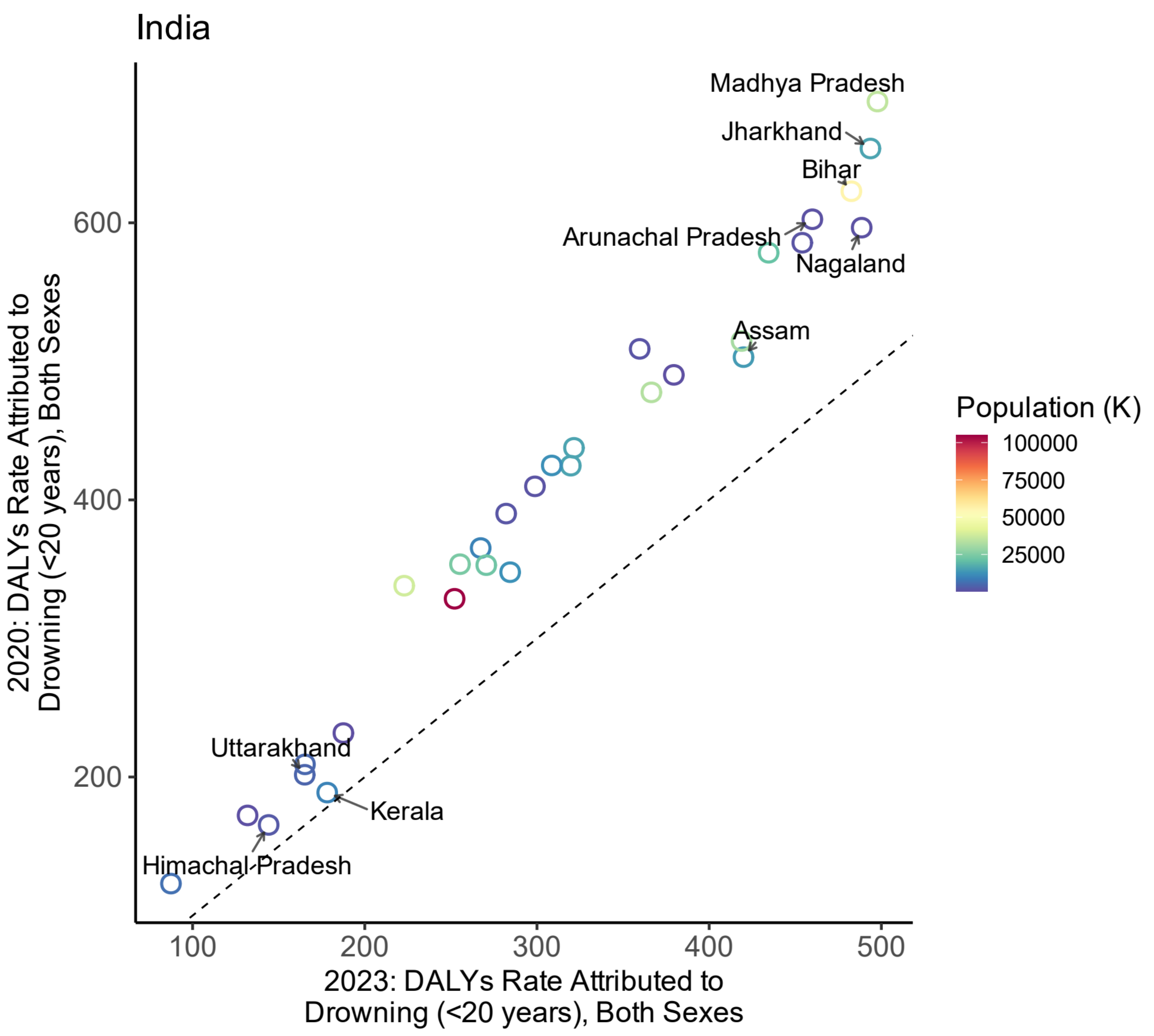
India
Madhya Pradesh
Jharkhand
Bihar
Arunachal Pradesh
Nagaland
Assam
Uttarakhand
Kerala
Himachal Pradesh
600
400
200
100
200
300
400
500
2020: DALYs Rate Attributed to Drowning (<20 years), Both Sexes
2023: DALYs Rate Attributed to Drowning (<20 years), Both Sexes
Population (K)
100000
75000
50000
25000

Supplementary Figure S2. Subnational drowning disability-adjusted life-year (DALY) rates, relative to sociodemographic development frontiers among population younger than 20 years in 14 countries, 1990–2023.

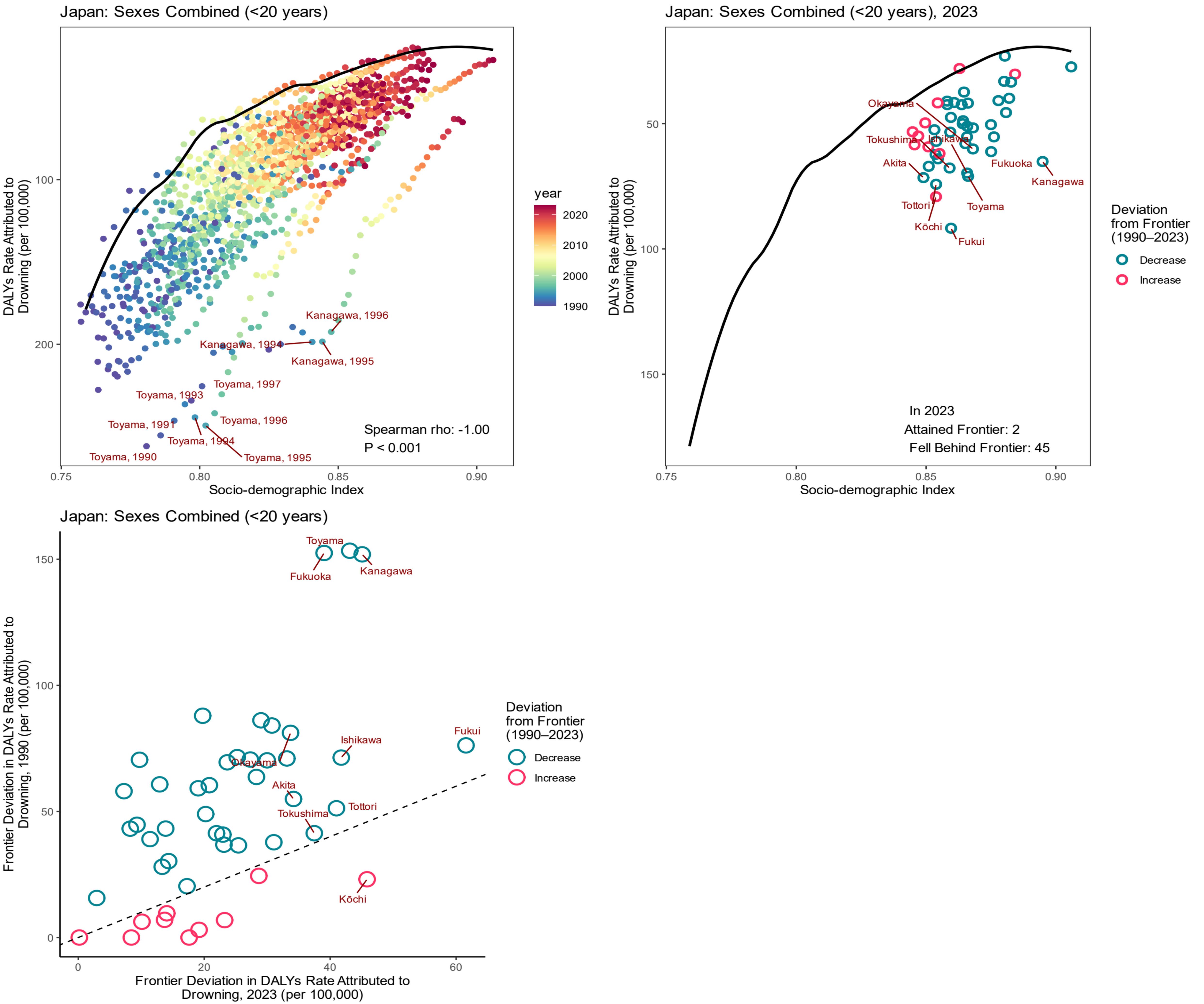

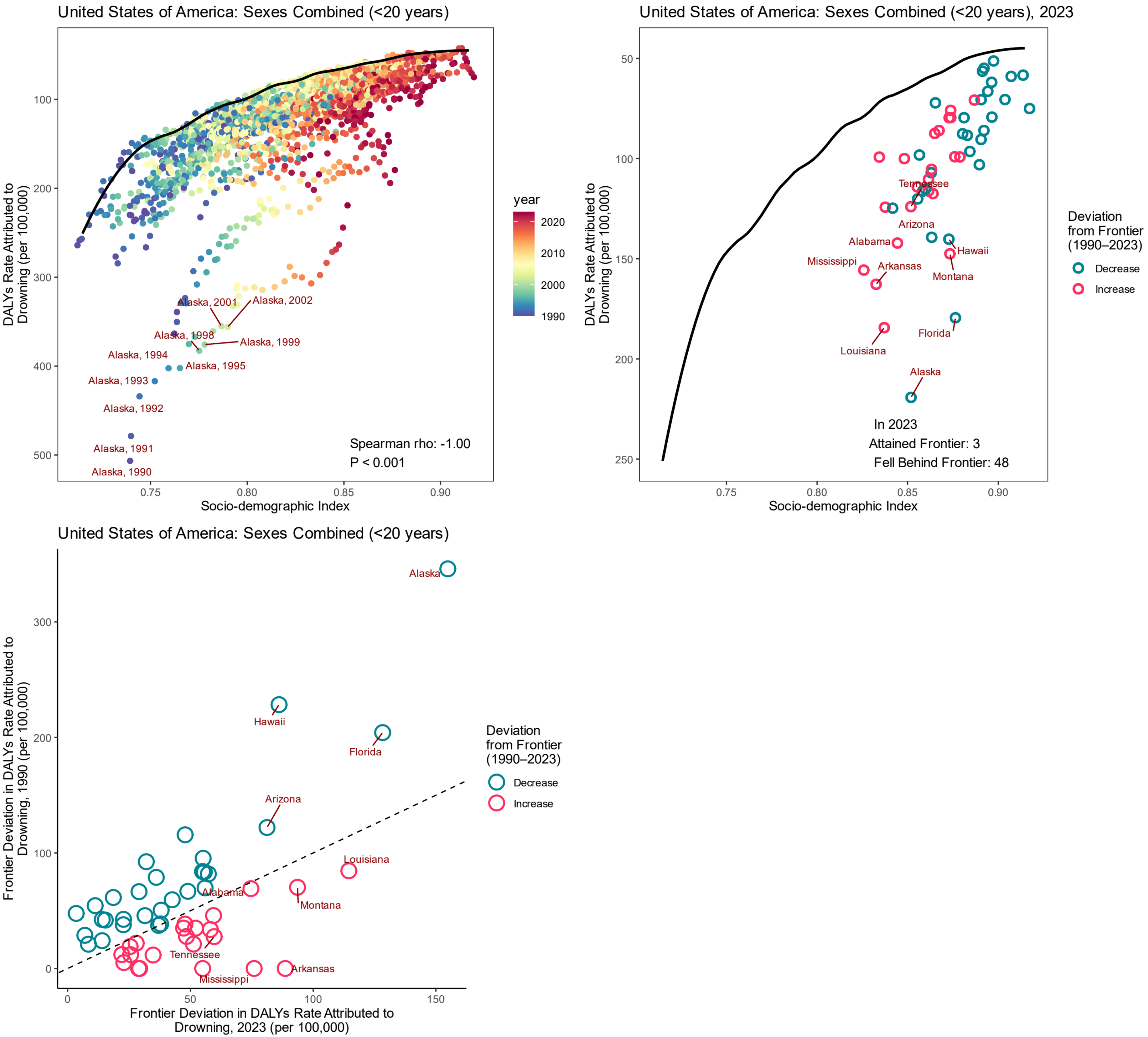
United States of America: Sexes Combined (<20 years)
DALYs Rate Attributed to Drowning (per 100,000)
Socio-demographic Index
year
2020
2010
2000
1990
Alaska, 2001
Alaska, 2002
Alaska, 1998
Alaska, 1999
Alaska, 1994
Alaska, 1995
Alaska, 1993
Alaska, 1992
Alaska, 1991
Alaska, 1990
Spearman rho: -1.00
P < 0.001
United States of America: Sexes Combined (<20 years), 2023
Tennessee
Arizona
Alabama
Hawaii
Mississippi
Arkansas
Montana
Florida
Louisiana
Alaska
In 2023
Attained Frontier: 3
Fell Behind Frontier: 48
Deviation from Frontier (1990–2023)
Decrease
Increase
United States of America: Sexes Combined (<20 years)
Frontier Deviation in DALYs Rate Attributed to Drowning, 1990 (per 100,000)
Frontier Deviation in DALYs Rate Attributed to Drowning, 2023 (per 100,000)
Alaska
Hawaii
Florida
Arizona
Louisiana
Alabama
Montana
Tennessee
Mississippi
Arkansas
Deviation from Frontier (1990–2023)
Decrease
Increase

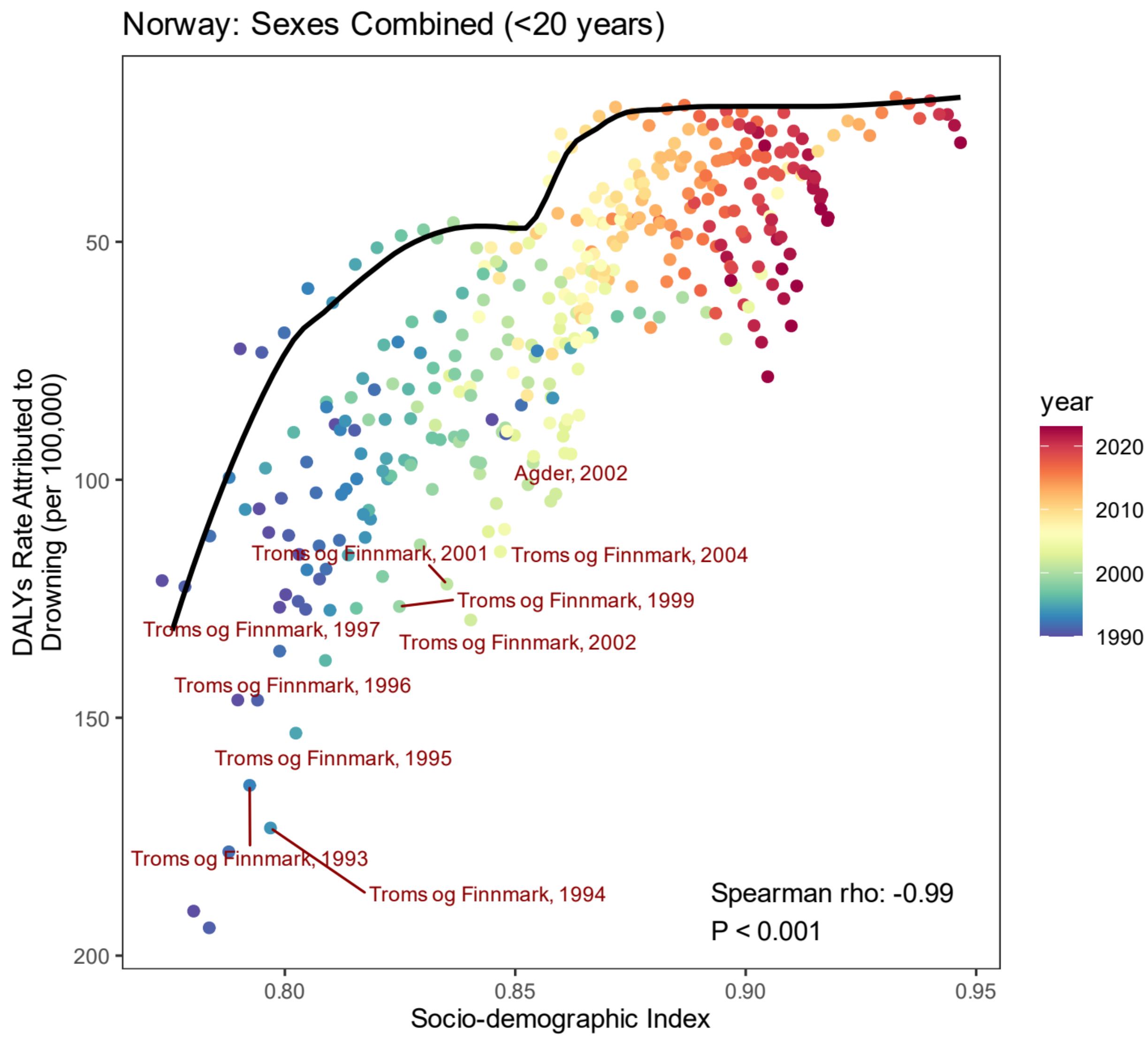

Norway: Sexes Combined (<20 years)
DALYs Rate Attributed to Drowning (per 100,000)
Socio-demographic Index
year
Agder, 2002
Troms og Finnmark, 2001
Troms og Finnmark, 2004
Troms og Finnmark, 1999
Troms og Finnmark, 1997
Troms og Finnmark, 2002
Troms og Finnmark, 1996
Troms og Finnmark, 1995
Troms og Finnmark, 1993
Troms og Finnmark, 1994
Spearman rho: -0.99
P < 0.001

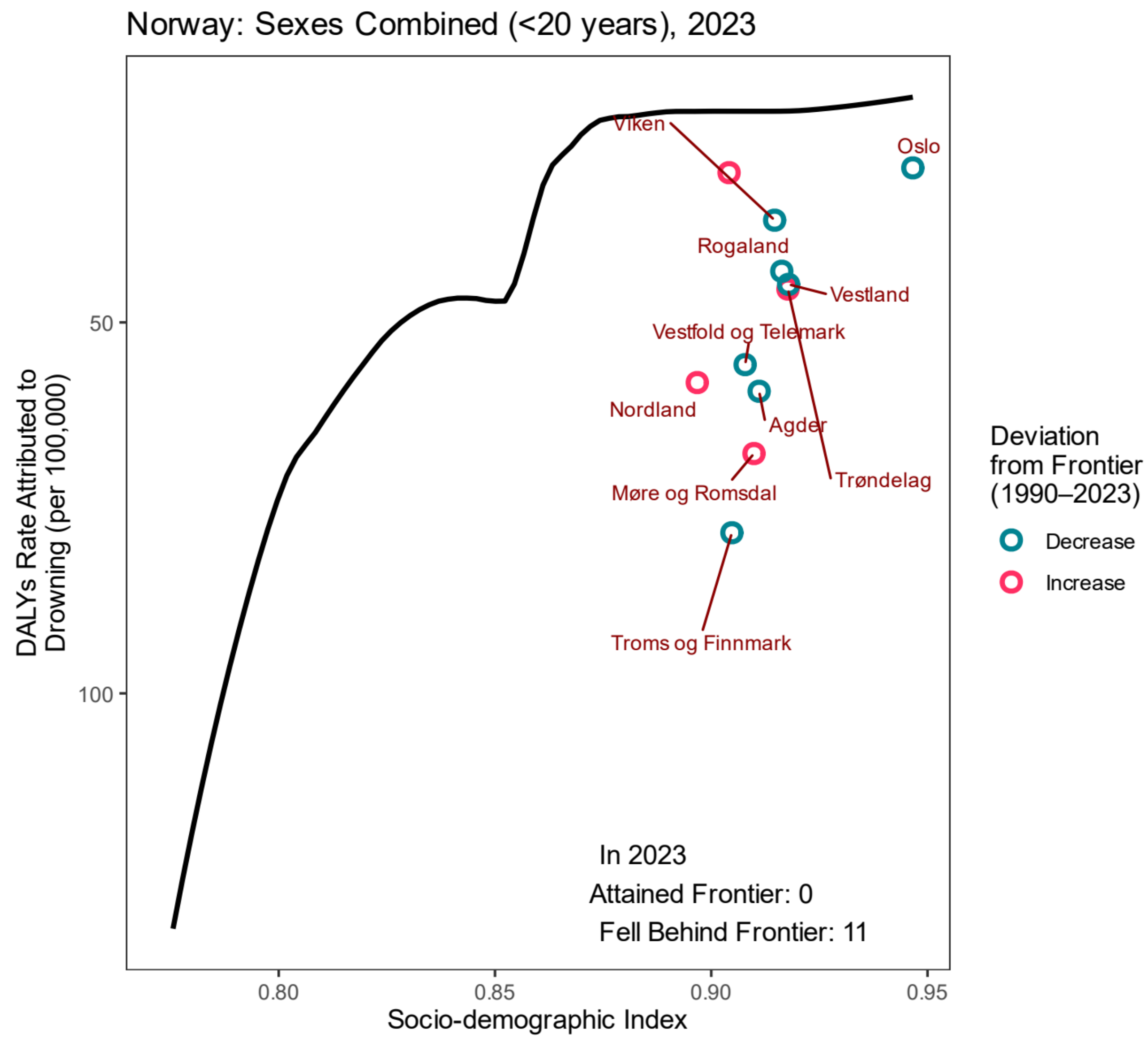

Norway: Sexes Combined (<20 years), 2023
DALYs Rate Attributed to Drowning (per 100,000)
Socio-demographic Index
Viken
Oslo
Rogaland
Vestland
Vestfold og Telemark
Nordland
Agder
Møre og Romsdal
Trøndelag
Troms og Finnmark
In 2023
Attained Frontier: 0
Fell Behind Frontier: 11
Deviation from Frontier (1990–2023)
Decrease
Increase

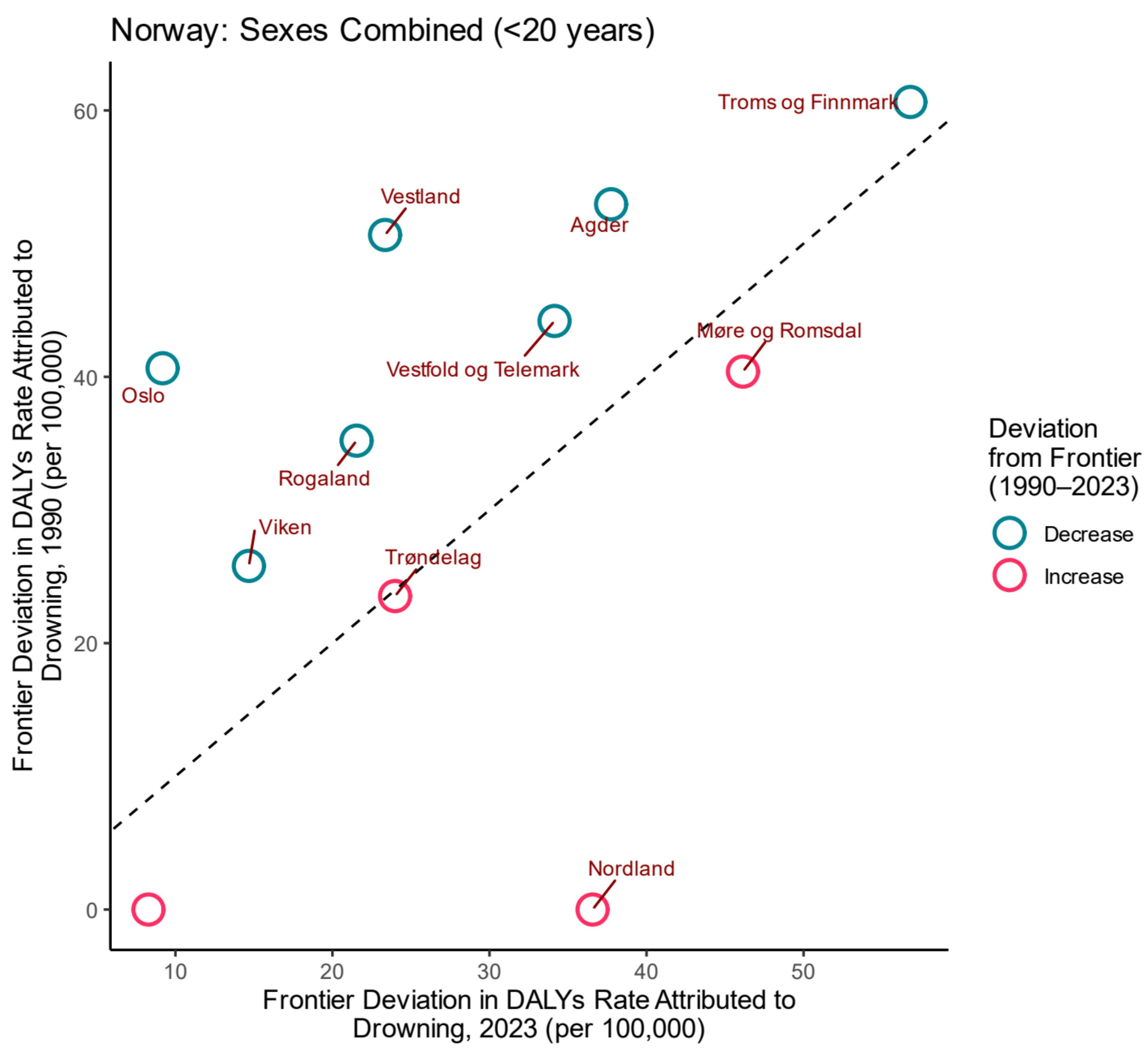

Norway: Sexes Combined (<20 years)
Frontier Deviation in DALYs Rate Attributed to Drowning, 1990 (per 100,000)
Frontier Deviation in DALYs Rate Attributed to Drowning, 2023 (per 100,000)
Troms og Finnmark
Vestland
Agder
Vestfold og Telemark
Møre og Romsdal
Oslo
Rogaland
Viken
Trøndelag
Nordland
Deviation from Frontier (1990–2023)
Decrease
Increase

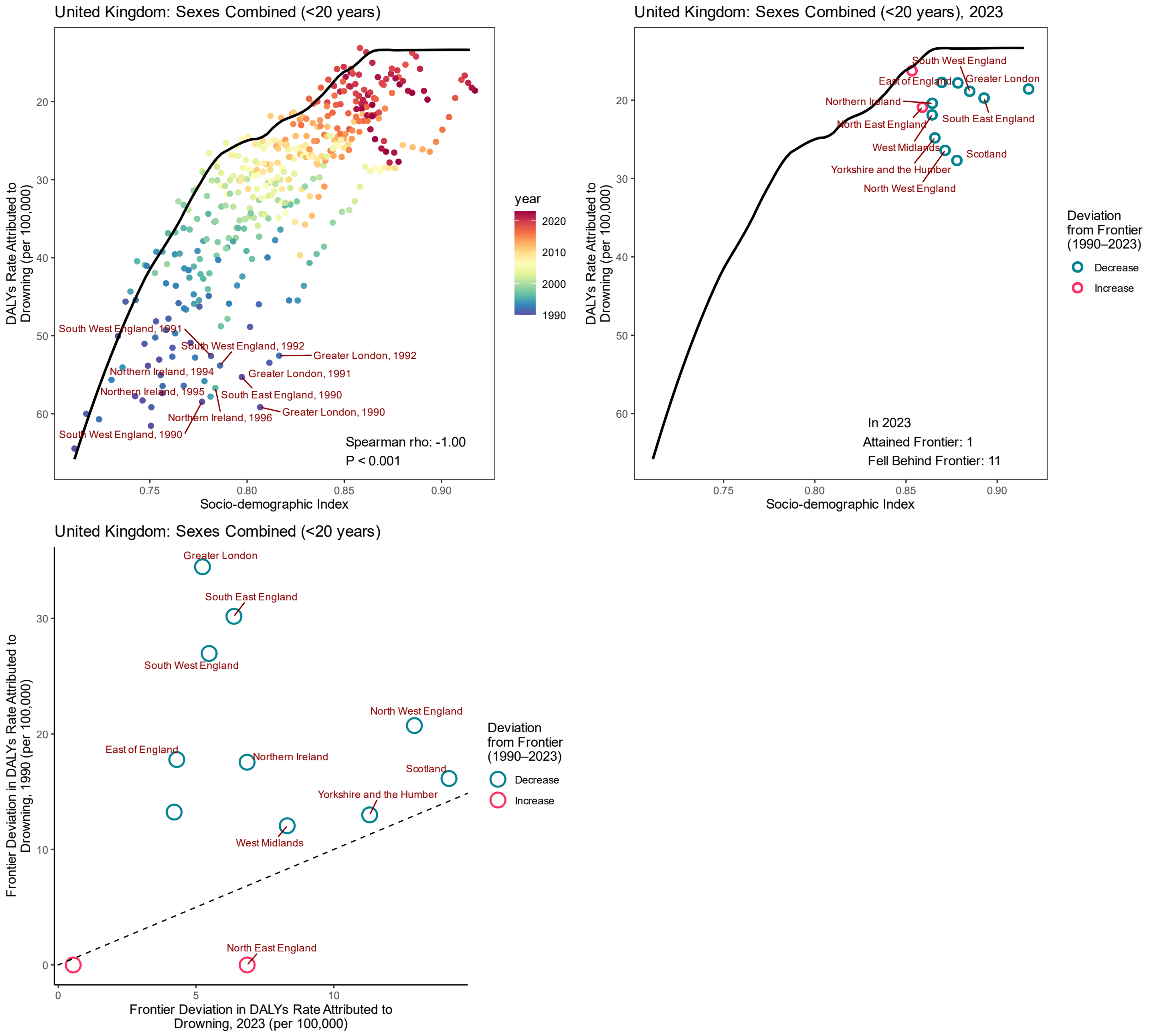
United Kingdom: Sexes Combined (<20 years)
DALYs Rate Attributed to Drowning (per 100,000)
Socio-demographic Index
year
Spearman rho: -1.00
P < 0.001
South West England, 1991
South West England, 1992
Greater London, 1992
Northern Ireland, 1994
Greater London, 1991
Northern Ireland, 1995
South East England, 1990
Greater London, 1990
Northern Ireland, 1996
South West England, 1990
United Kingdom: Sexes Combined (<20 years), 2023
South West England
East of England
Greater London
Northern Ireland
North East England
South East England
West Midlands
Scotland
Yorkshire and the Humber
North West England
In 2023
Attained Frontier: 1
Fell Behind Frontier: 11
Deviation from Frontier (1990–2023)
Decrease
Increase
United Kingdom: Sexes Combined (<20 years)
Frontier Deviation in DALYs Rate Attributed to Drowning, 1990 (per 100,000)
Frontier Deviation in DALYs Rate Attributed to Drowning, 2023 (per 100,000)
Greater London
South East England
South West England
North West England
East of England
Northern Ireland
Scotland
Yorkshire and the Humber
West Midlands
North East England
Deviation from Frontier (1990–2023)
Decrease
Increase

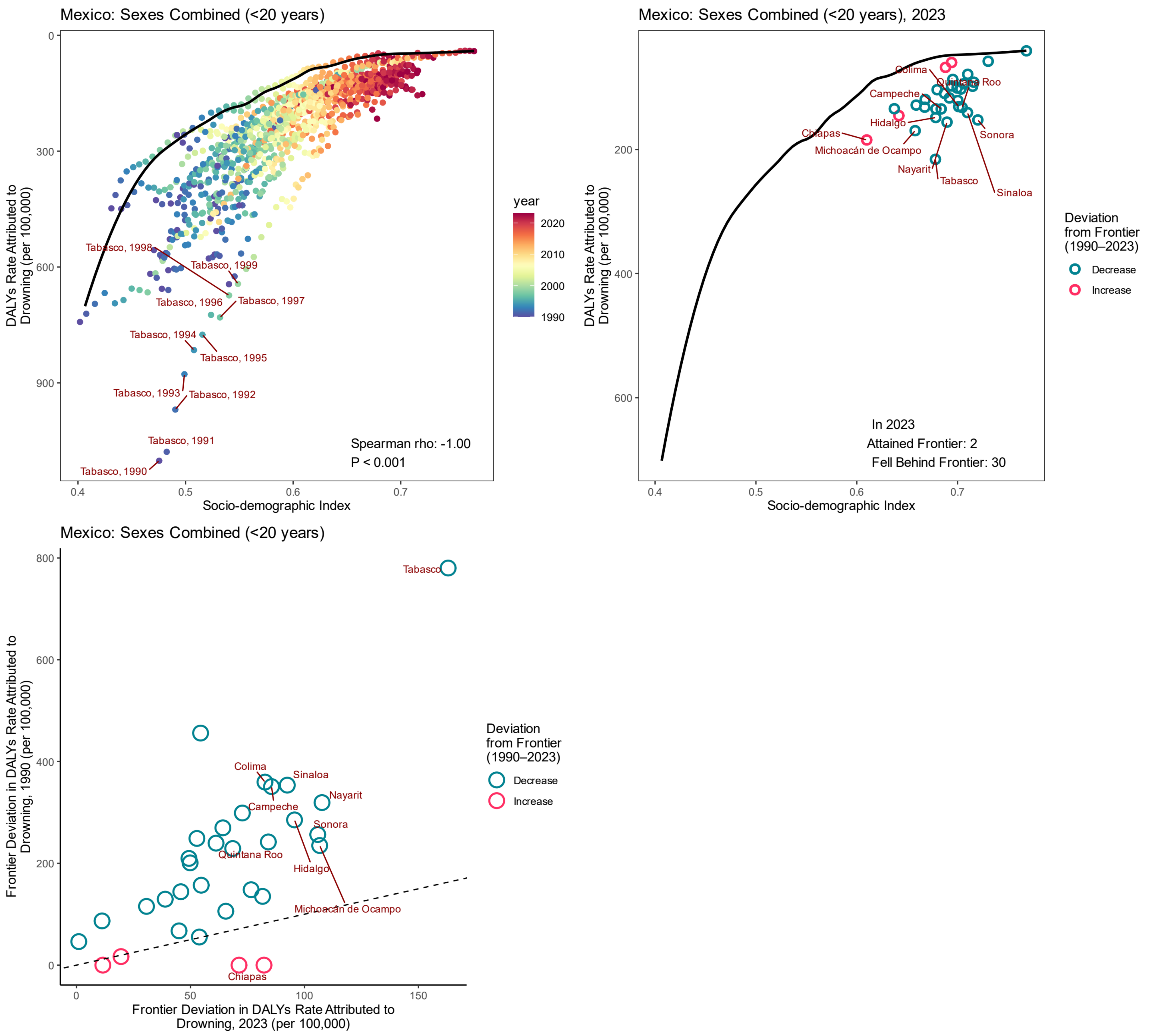

Mexico: Sexes Combined (<20 years)
DALYs Rate Attributed to Drowning (per 100,000)
Socio-demographic Index
year
2020
2010
2000
1990
Tabasco, 1998
Tabasco, 1999
Tabasco, 1996
Tabasco, 1997
Tabasco, 1994
Tabasco, 1995
Tabasco, 1993
Tabasco, 1992
Tabasco, 1991
Tabasco, 1990
Spearman rho: -1.00
P < 0.001
Mexico: Sexes Combined (<20 years), 2023
Colima
Quintana Roo
Campeche
Hidalgo
Chiapas
Michoacán de Ocampo
Sonora
Nayarit
Tabasco
Sinaloa
In 2023
Attained Frontier: 2
Fell Behind Frontier: 30
Deviation from Frontier (1990–2023)
Decrease
Increase
Mexico: Sexes Combined (<20 years)
Frontier Deviation in DALYs Rate Attributed to Drowning, 1990 (per 100,000)
Frontier Deviation in DALYs Rate Attributed to Drowning, 2023 (per 100,000)
Tabasco
Colima
Sinaloa
Nayarit
Campeche
Sonora
Quintana Roo
Hidalgo
Michoacán de Ocampo
Chiapas
Deviation from Frontier (1990–2023)
Decrease
Increase

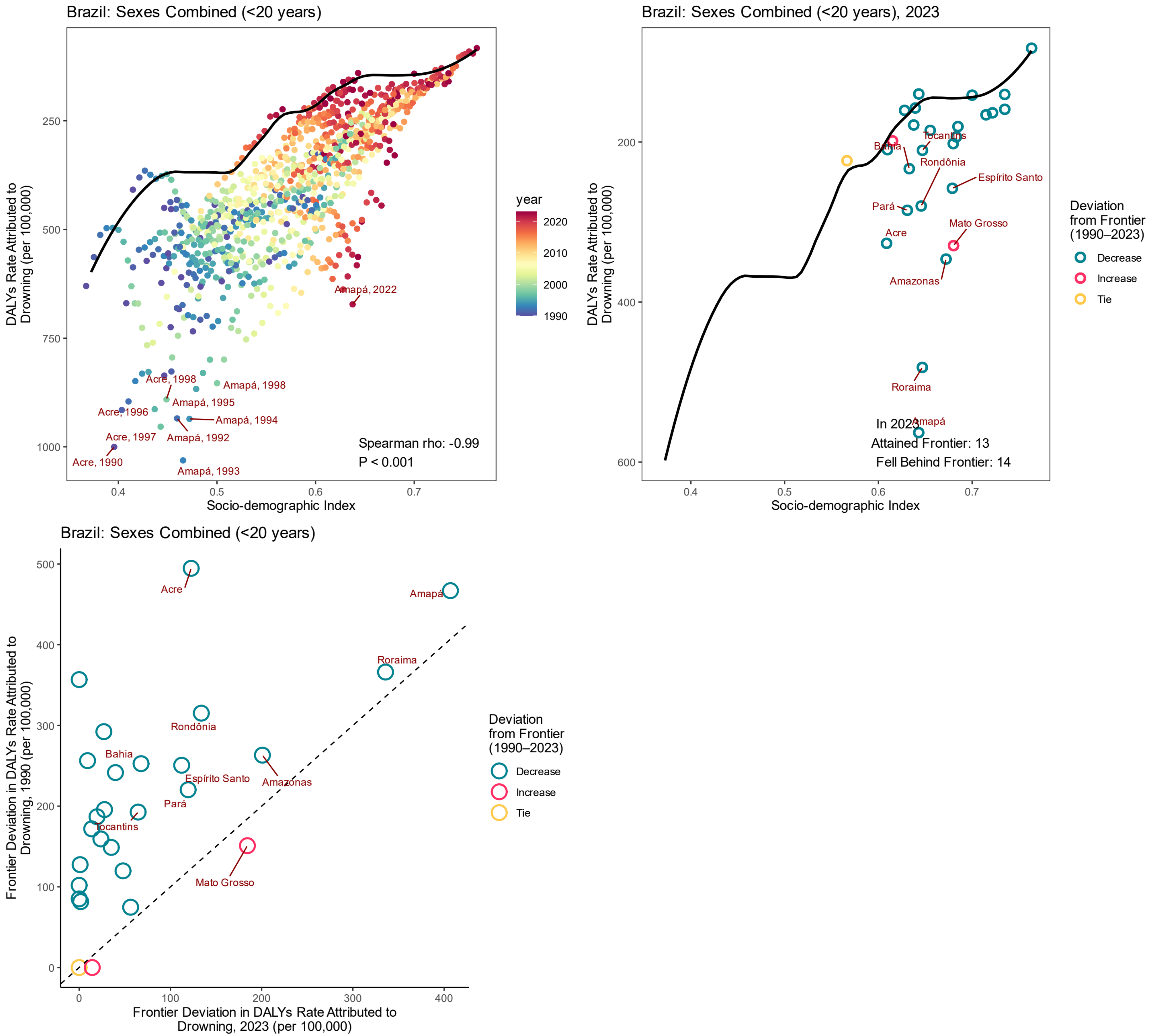

Brazil: Sexes Combined (<20 years)
DALYs Rate Attributed to Drowning (per 100,000)
Socio-demographic Index
year
2020
2010
2000
1990
Amapá, 2022
Acre, 1998
Amapá, 1998
Amapá, 1995
Acre, 1996
Amapá, 1994
Acre, 1997
Amapá, 1992
Acre, 1990
Amapá, 1993
Spearman rho: -0.99
P < 0.001
Brazil: Sexes Combined (<20 years), 2023
Tocantins
Bahia
Rondônia
Espírito Santo
Pará
Mato Grosso
Acre
Amazonas
Roraima
Amapá
In 2023
Attained Frontier: 13
Fell Behind Frontier: 14
Deviation from Frontier (1990–2023)
Decrease
Increase
Tie
Brazil: Sexes Combined (<20 years)
Frontier Deviation in DALYs Rate Attributed to Drowning, 1990 (per 100,000)
Frontier Deviation in DALYs Rate Attributed to Drowning, 2023 (per 100,000)
Acre
Amapá
Roraima
Rondônia
Bahia
Espírito Santo
Amazonas
Pará
Tocantins
Mato Grosso
Deviation from Frontier (1990–2023)
Decrease
Increase
Tie

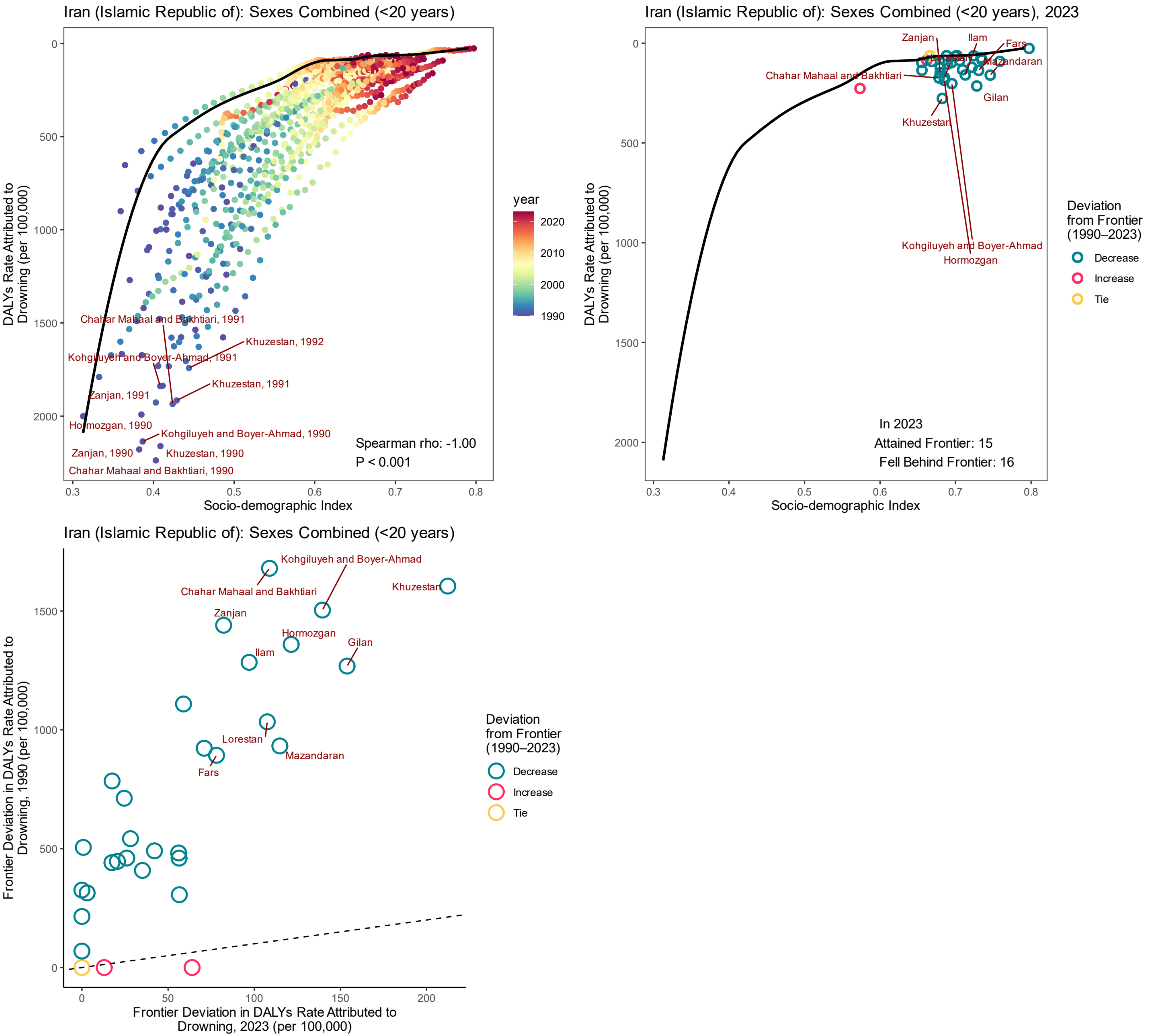

Iran (Islamic Republic of): Sexes Combined (<20 years)
DALYs Rate Attributed to Drowning (per 100,000)
Socio-demographic Index
year
2020
2010
2000
1990
Chahar Mahaal and Bakhtiari, 1991
Khuzestan, 1992
Kohgiluyeh and Boyer-Ahmad, 1991
Khuzestan, 1991
Zanjan, 1991
Hormozgan, 1990
Kohgiluyeh and Boyer-Ahmad, 1990
Zanjan, 1990
Khuzestan, 1990
Chahar Mahaal and Bakhtiari, 1990
Spearman rho: -1.00
P < 0.001
Iran (Islamic Republic of): Sexes Combined (<20 years), 2023
Zanjan
Ilam
Fars
Lorestan
Mazandaran
Chahar Mahaal and Bakhtiari
Gilan
Khuzestan
Kohgiluyeh and Boyer-Ahmad
Hormozgan
In 2023
Attained Frontier: 15
Fell Behind Frontier: 16
Deviation from Frontier (1990–2023)
Decrease
Increase
Tie
Iran (Islamic Republic of): Sexes Combined (<20 years)
Frontier Deviation in DALYs Rate Attributed to Drowning, 1990 (per 100,000)
Frontier Deviation in DALYs Rate Attributed to Drowning, 2023 (per 100,000)
Kohgiluyeh and Boyer-Ahmad
Chahar Mahaal and Bakhtiari
Khuzestan
Zanjan
Hormozgan
Gilan
Ilam
Lorestan
Mazandaran
Fars
Deviation from Frontier (1990–2023)
Decrease
Increase
Tie

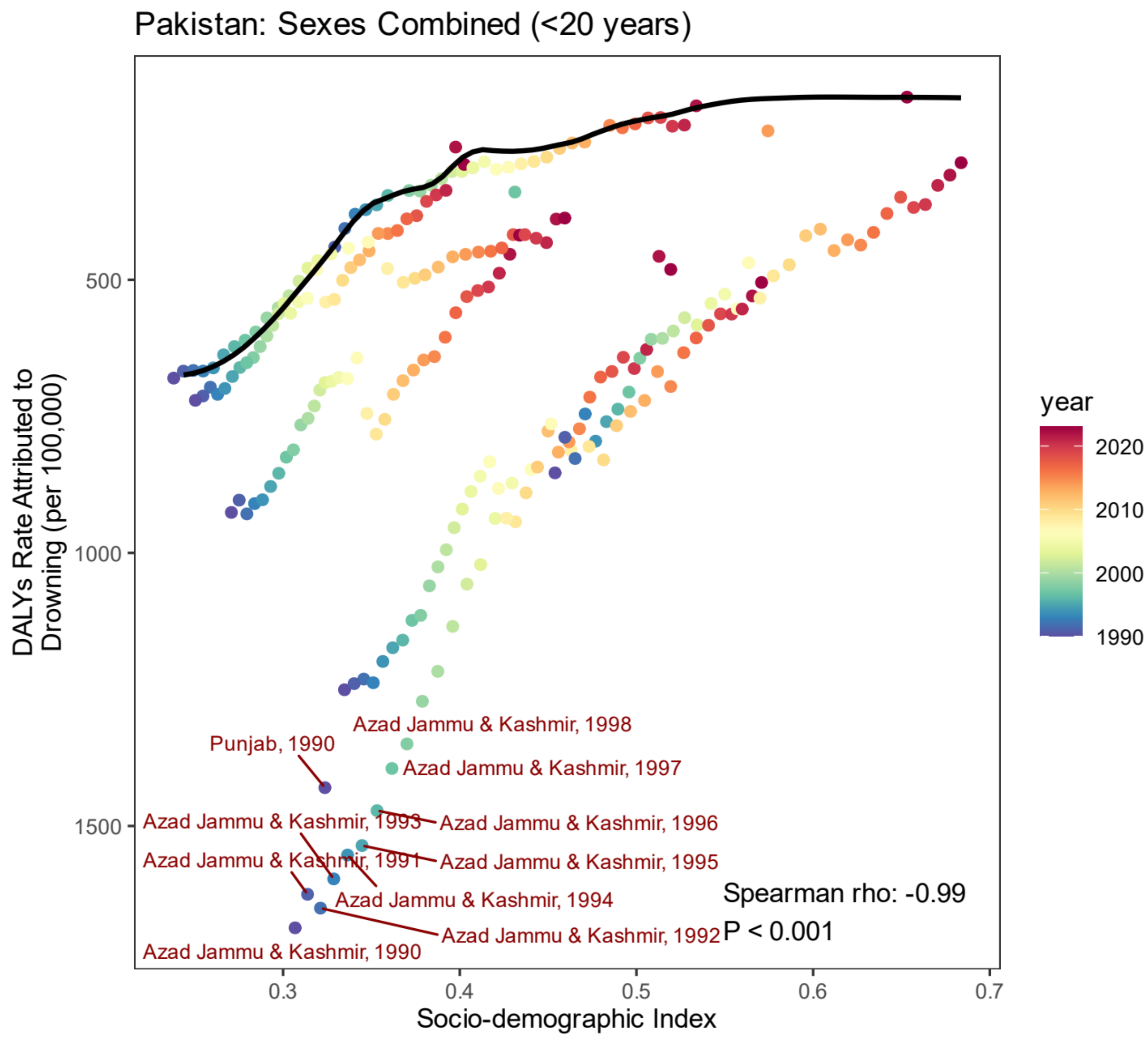
Pakistan: Sexes Combined (<20 years)
DALYs Rate Attributed to Drowning (per 100,000)
500
1000
1500
0.3
0.4
0.5
0.6
0.7
Socio-demographic Index
year
2020
2010
2000
1990
Azad Jammu & Kashmir, 1998
Punjab, 1990
Azad Jammu & Kashmir, 1997
Azad Jammu & Kashmir, 1993
Azad Jammu & Kashmir, 1996
Azad Jammu & Kashmir, 1991
Azad Jammu & Kashmir, 1995
Azad Jammu & Kashmir, 1994
Azad Jammu & Kashmir, 1992
Azad Jammu & Kashmir, 1990
Spearman rho: -0.99
P < 0.001

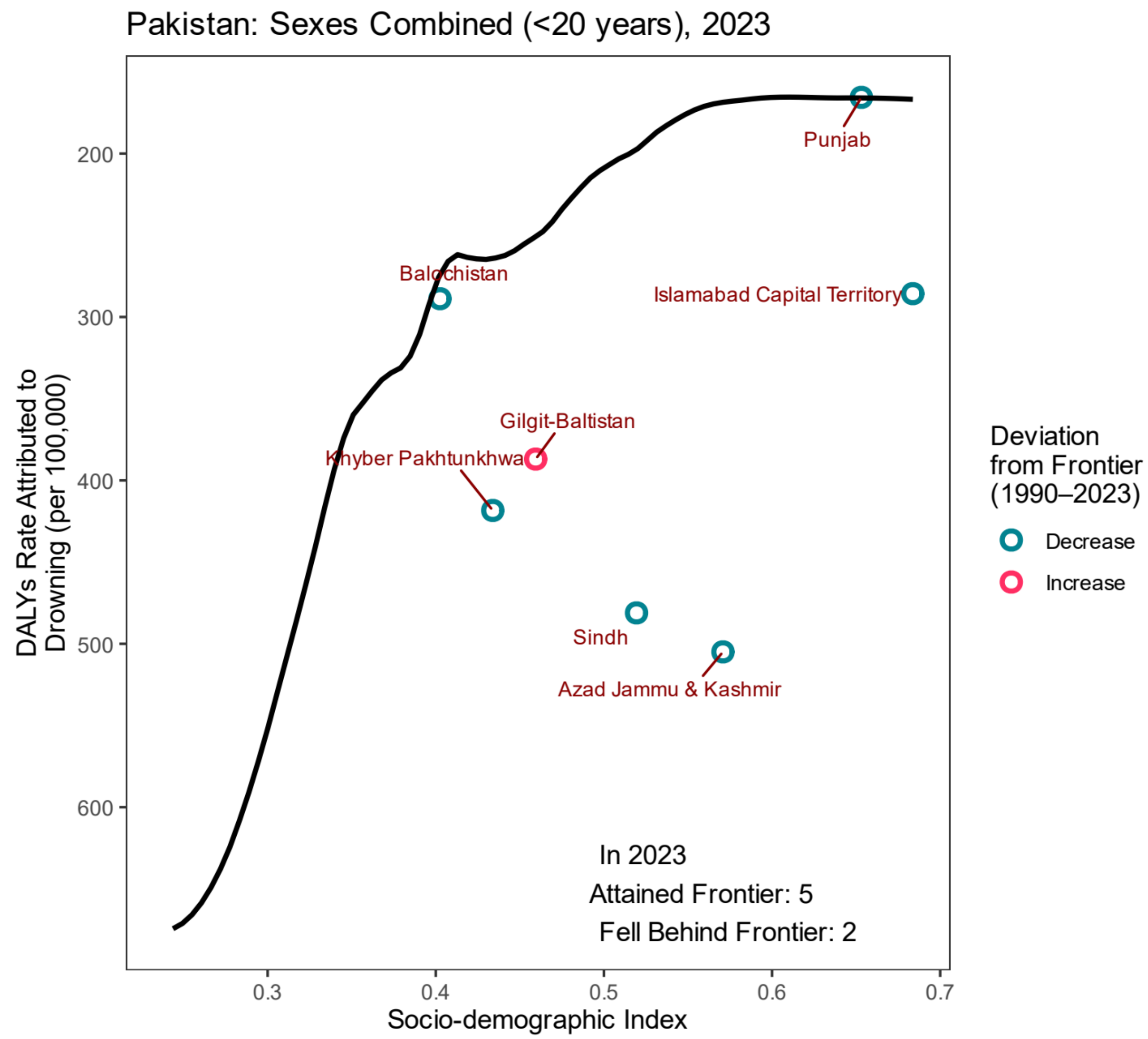
Pakistan: Sexes Combined (<20 years), 2023
DALYs Rate Attributed to Drowning (per 100,000)
200
300
400
500
600
0.3
0.4
0.5
0.6
0.7
Socio-demographic Index
Punjab
Balochistan
Islamabad Capital Territory
Gilgit-Baltistan
Khyber Pakhtunkhwa
Sindh
Azad Jammu & Kashmir
In 2023
Attained Frontier: 5
Fell Behind Frontier: 2
Deviation from Frontier (1990–2023)
Decrease
Increase

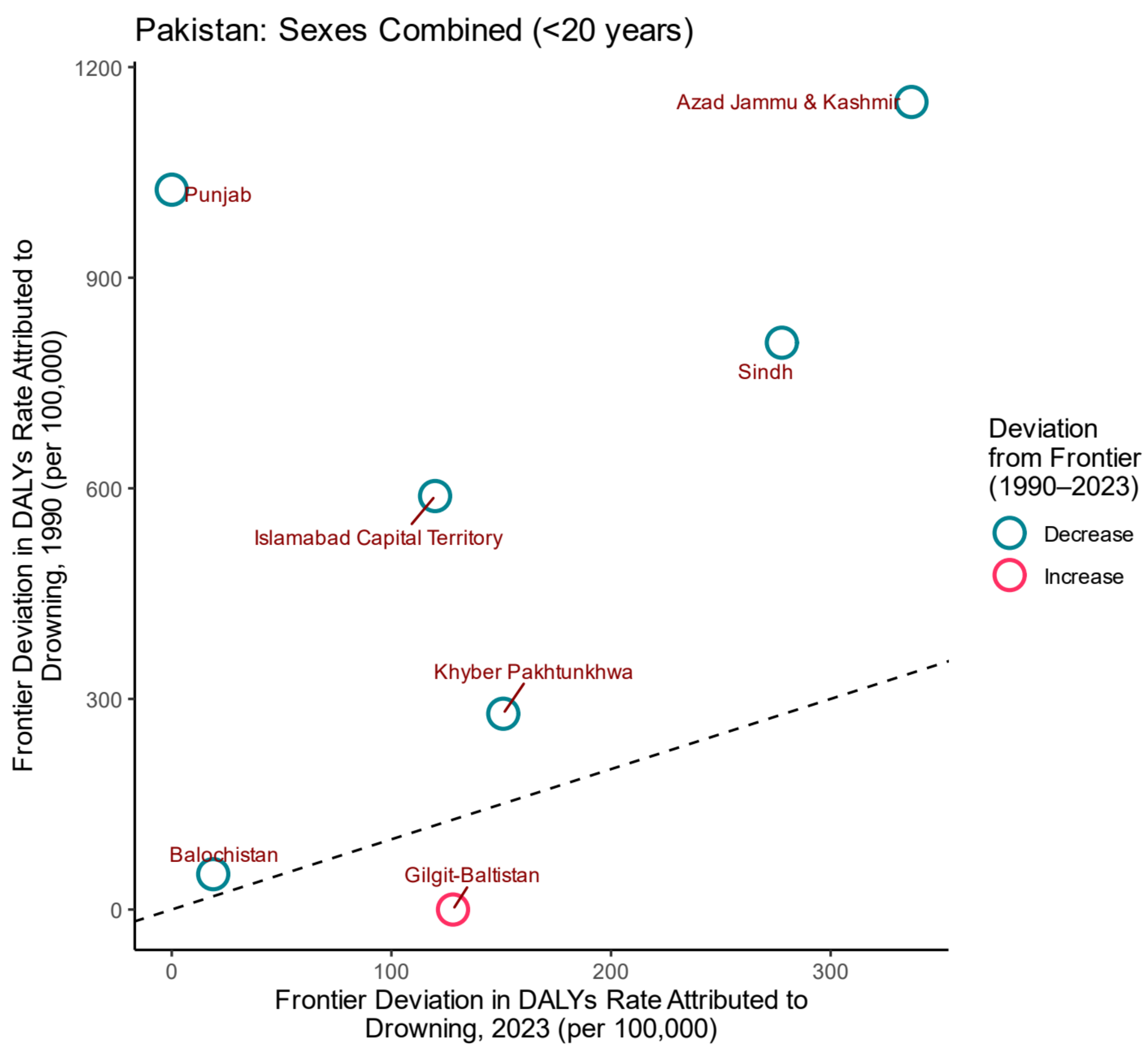
Pakistan: Sexes Combined (<20 years)
Frontier Deviation in DALYs Rate Attributed to Drowning, 1990 (per 100,000)
1200
900
600
300
0
Frontier Deviation in DALYs Rate Attributed to Drowning, 2023 (per 100,000)
0
100
200
300
Azad Jammu & Kashmir
Punjab
Sindh
Islamabad Capital Territory
Khyber Pakhtunkhwa
Balochistan
Gilgit-Baltistan
Deviation from Frontier (1990–2023)
Decrease
Increase

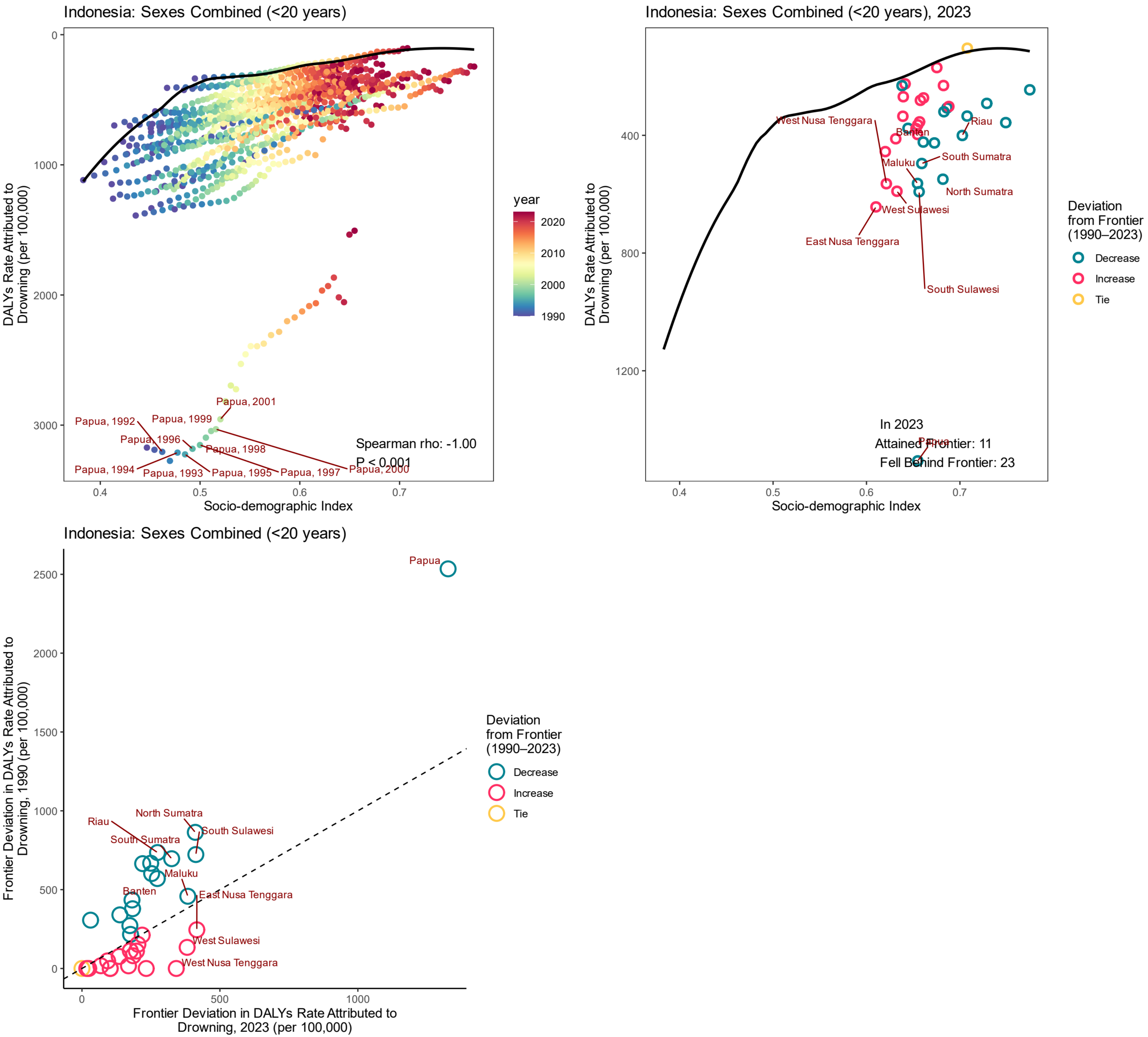

Indonesia: Sexes Combined (<20 years)
DALYs Rate Attributed to Drowning (per 100,000)
Socio-demographic Index
year
2020
2010
2000
1990
Papua, 2001
Papua, 1992
Papua, 1999
Papua, 1996
Papua, 1998
Papua, 1994
Papua, 1993
Papua, 1995
Papua, 1997
Papua, 2000
Spearman rho: -1.00
P < 0.001
Indonesia: Sexes Combined (<20 years), 2023
West Nusa Tenggara
Banten
Riau
South Sumatra
Maluku
North Sumatra
West Sulawesi
East Nusa Tenggara
South Sulawesi
In 2023
Attained Frontier: 11
Papua
Fell Behind Frontier: 23
Deviation from Frontier (1990–2023)
Decrease
Increase
Tie
Indonesia: Sexes Combined (<20 years)
Frontier Deviation in DALYs Rate Attributed to Drowning, 1990 (per 100,000)
Frontier Deviation in DALYs Rate Attributed to Drowning, 2023 (per 100,000)
Papua
Riau
North Sumatra
South Sulawesi
South Sumatra
Maluku
Banten
East Nusa Tenggara
West Sulawesi
West Nusa Tenggara
Deviation from Frontier (1990–2023)
Decrease
Increase
Tie

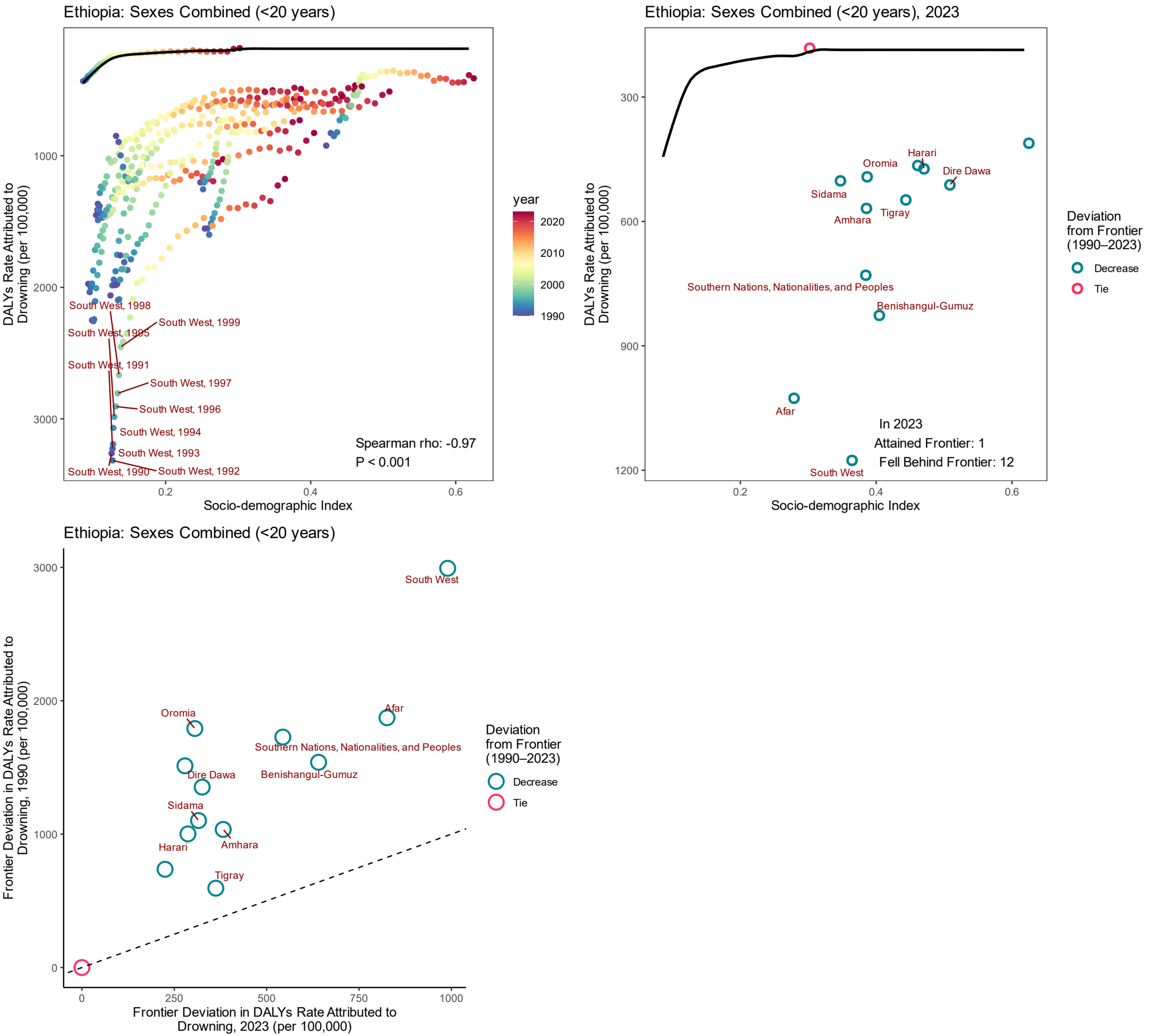

Ethiopia: Sexes Combined (<20 years)
DALYs Rate Attributed to Drowning (per 100,000)
Socio-demographic Index
year
2020
2010
2000
1990
South West, 1998
South West, 1999
South West, 1995
South West, 1991
South West, 1997
South West, 1996
South West, 1994
South West, 1993
South West, 1990
South West, 1992
Spearman rho: -0.97
P < 0.001
Ethiopia: Sexes Combined (<20 years), 2023
Harari
Oromia
Dire Dawa
Sidama
Tigray
Amhara
Southern Nations, Nationalities, and Peoples
Benishangul-Gumuz
Afar
South West
In 2023
Attained Frontier: 1
Fell Behind Frontier: 12
Deviation from Frontier (1990–2023)
Decrease
Tie
Ethiopia: Sexes Combined (<20 years)
Frontier Deviation in DALYs Rate Attributed to Drowning, 1990 (per 100,000)
Frontier Deviation in DALYs Rate Attributed to Drowning, 2023 (per 100,000)
South West
Oromia
Afar
Southern Nations, Nationalities, and Peoples
Dire Dawa
Benishangul-Gumuz
Sidama
Harari
Amhara
Tigray
Deviation from Frontier (1990–2023)
Decrease
Tie

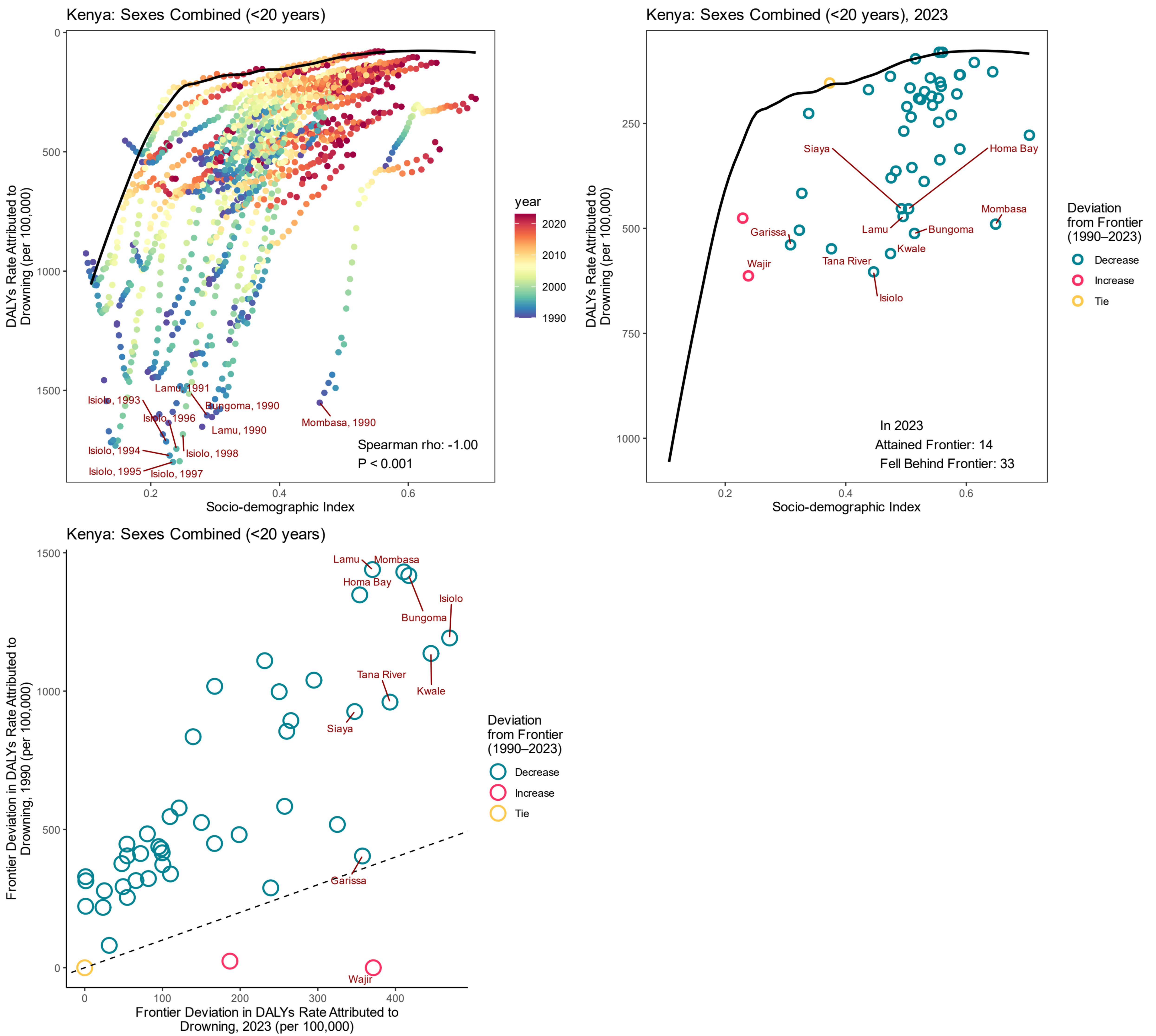

Kenya: Sexes Combined (<20 years)
DALYs Rate Attributed to Drowning (per 100,000)
Socio-demographic Index
year
2020
2010
2000
1990
Isiolo, 1993
Lamu, 1991
Bungoma, 1990
Isiolo, 1996
Lamu, 1990
Mombasa, 1990
Isiolo, 1994
Isiolo, 1998
Isiolo, 1995
Isiolo, 1997
Spearman rho: -1.00
P < 0.001
Kenya: Sexes Combined (<20 years), 2023
Siaya
Homa Bay
Mombasa
Garissa
Lamu
Bungoma
Kwale
Tana River
Wajir
Isiolo
In 2023
Attained Frontier: 14
Fell Behind Frontier: 33
Deviation from Frontier (1990–2023)
Decrease
Increase
Tie
Kenya: Sexes Combined (<20 years)
Frontier Deviation in DALYs Rate Attributed to Drowning, 1990 (per 100,000)
Frontier Deviation in DALYs Rate Attributed to Drowning, 2023 (per 100,000)
Lamu
Mombasa
Homa Bay
Isiolo
Bungoma
Tana River
Kwale
Siaya
Garissa
Wajir
Deviation from Frontier (1990–2023)
Decrease
Increase
Tie

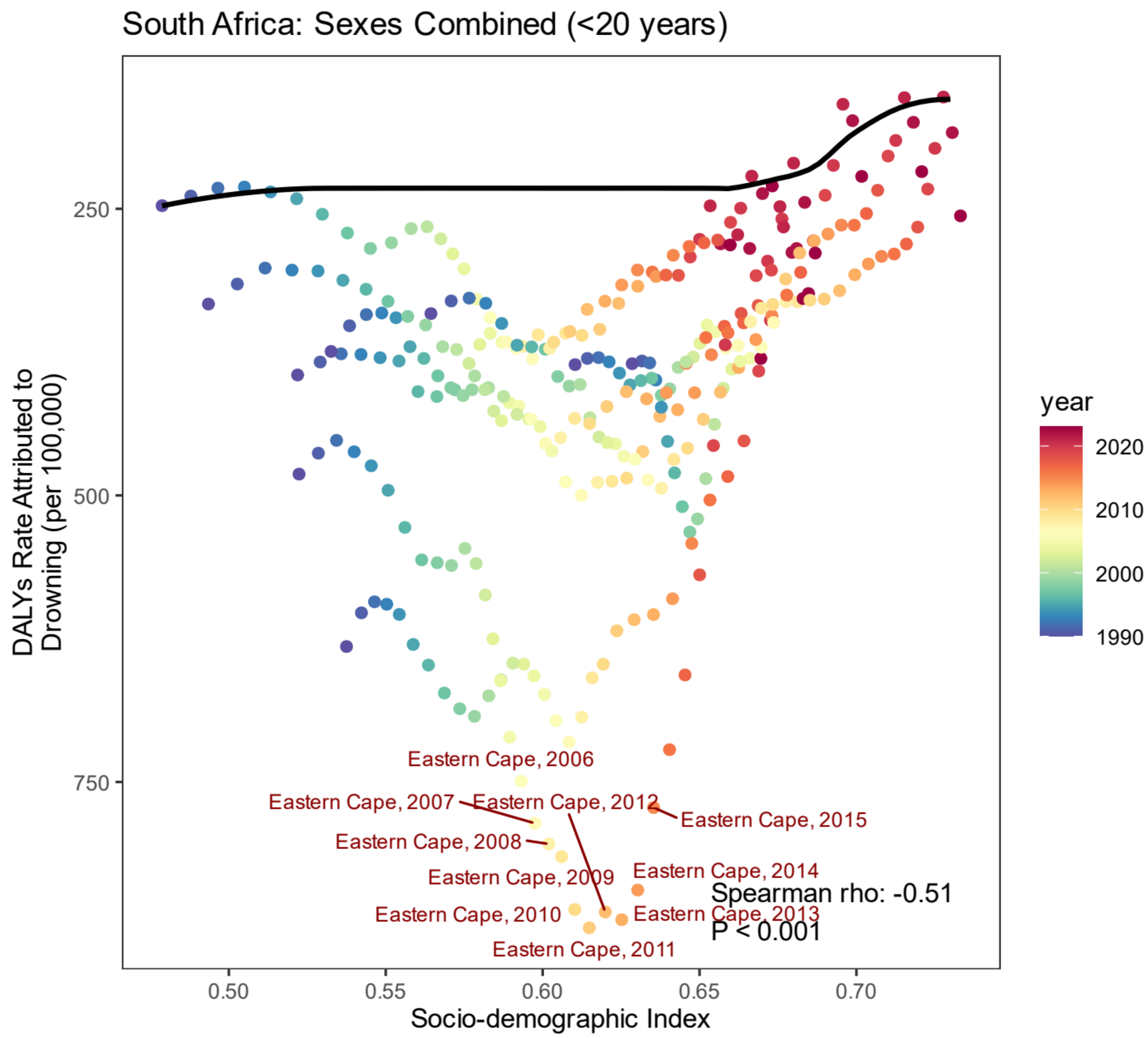
South Africa: Sexes Combined (<20 years)
DALYs Rate Attributed to Drowning (per 100,000)
Socio-demographic Index
year
2020
2010
2000
1990
Eastern Cape, 2006
Eastern Cape, 2007
Eastern Cape, 2012
Eastern Cape, 2015
Eastern Cape, 2008
Eastern Cape, 2009
Eastern Cape, 2014
Eastern Cape, 2010
Eastern Cape, 2013
Eastern Cape, 2011
Spearman rho: -0.51
P < 0.001
250
500
750
0.50
0.55
0.60
0.65
0.70

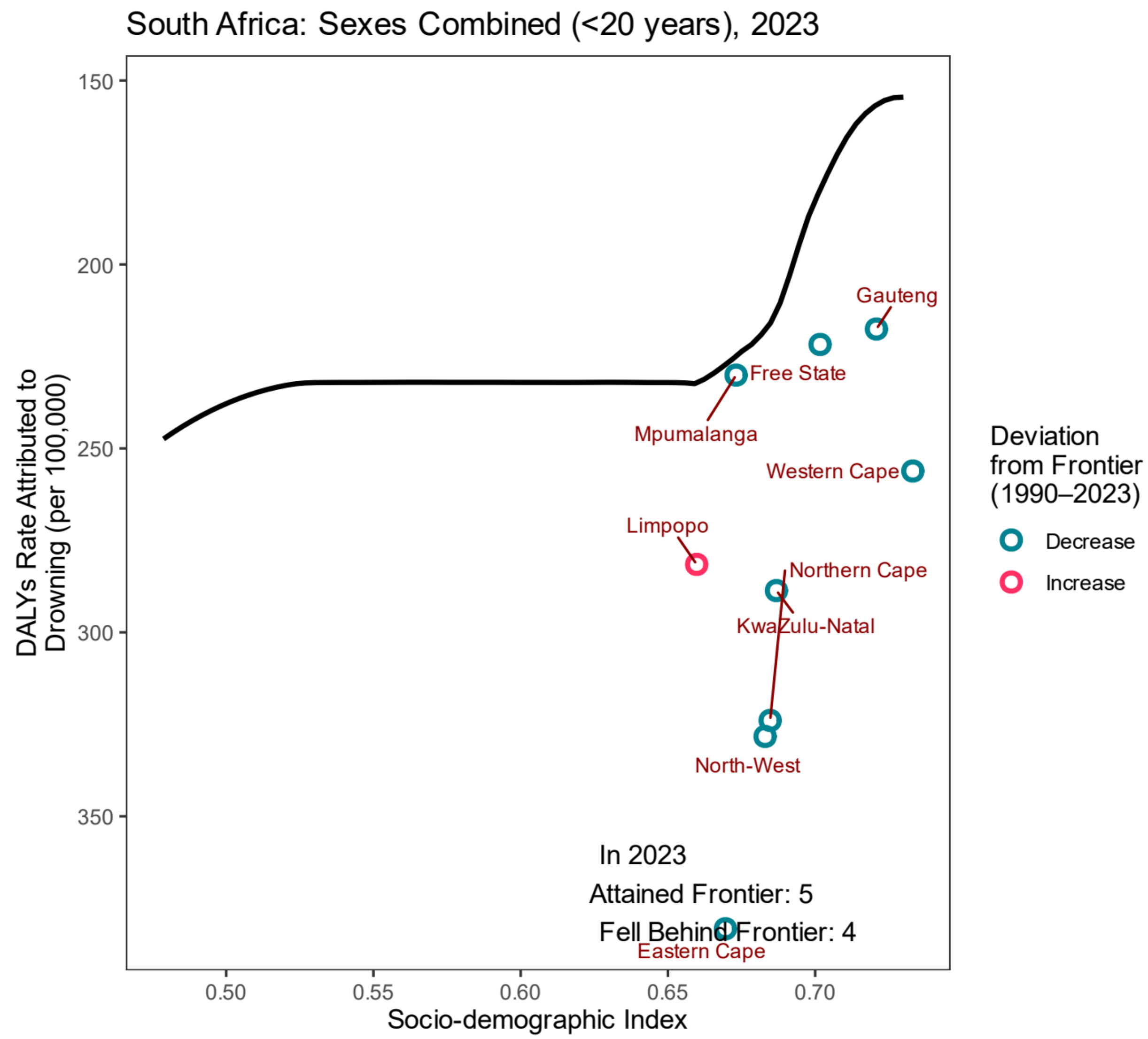
South Africa: Sexes Combined (<20 years), 2023
DALYs Rate Attributed to Drowning (per 100,000)
Socio-demographic Index
Gauteng
Free State
Mpumalanga
Western Cape
Limpopo
Northern Cape
KwaZulu-Natal
North-West
Eastern Cape
In 2023
Attained Frontier: 5
Fell Behind Frontier: 4
Deviation from Frontier (1990–2023)
Decrease
Increase
150
200
250
300
350
0.50
0.55
0.60
0.65
0.70

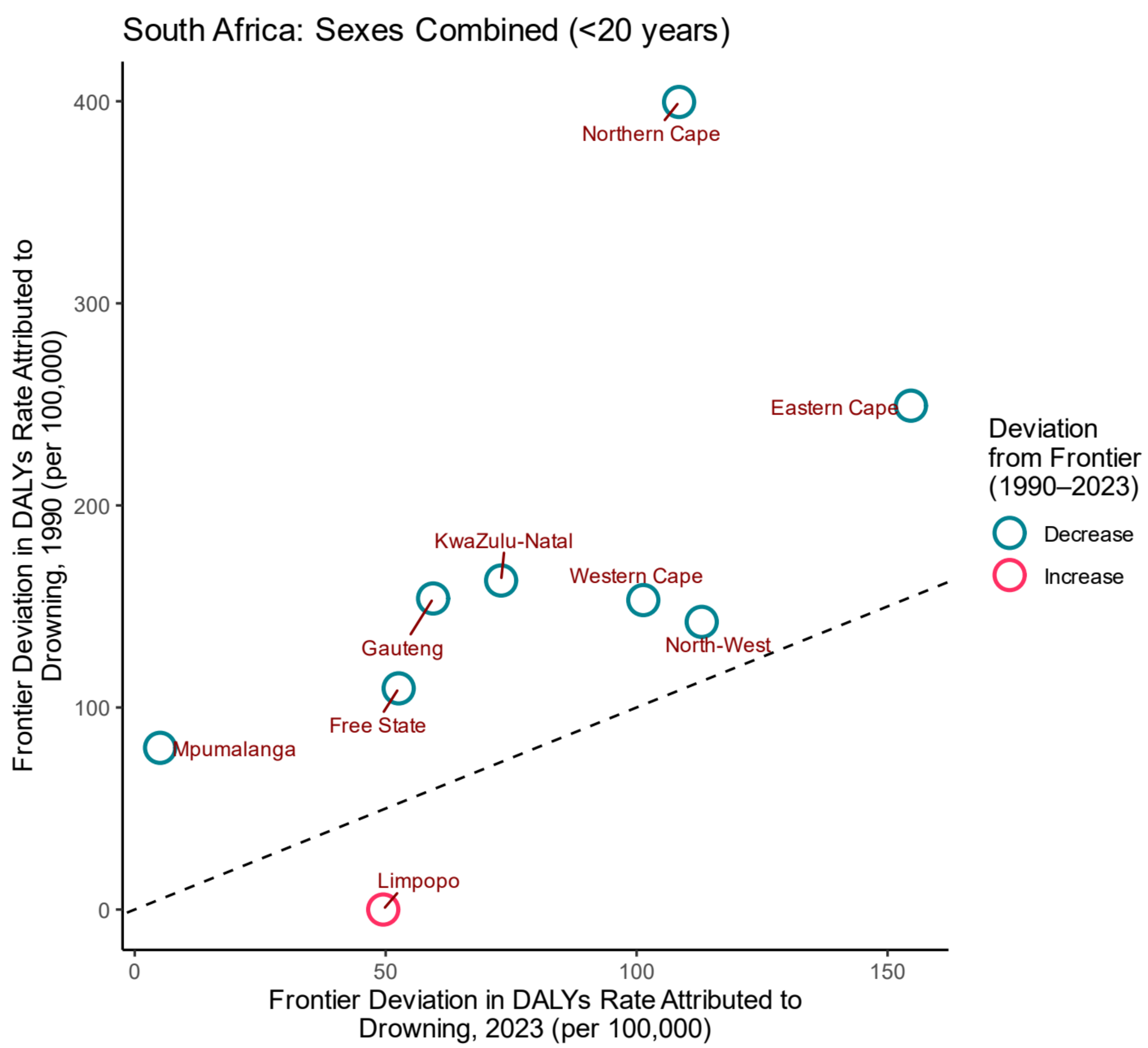
South Africa: Sexes Combined (<20 years)
Frontier Deviation in DALYs Rate Attributed to Drowning, 1990 (per 100,000)
Frontier Deviation in DALYs Rate Attributed to Drowning, 2023 (per 100,000)
Northern Cape
Eastern Cape
KwaZulu-Natal
Western Cape
Gauteng
North-West
Free State
Mpumalanga
Limpopo
Deviation from Frontier (1990–2023)
Decrease
Increase
0
100
200
300
400
0
50
100
150

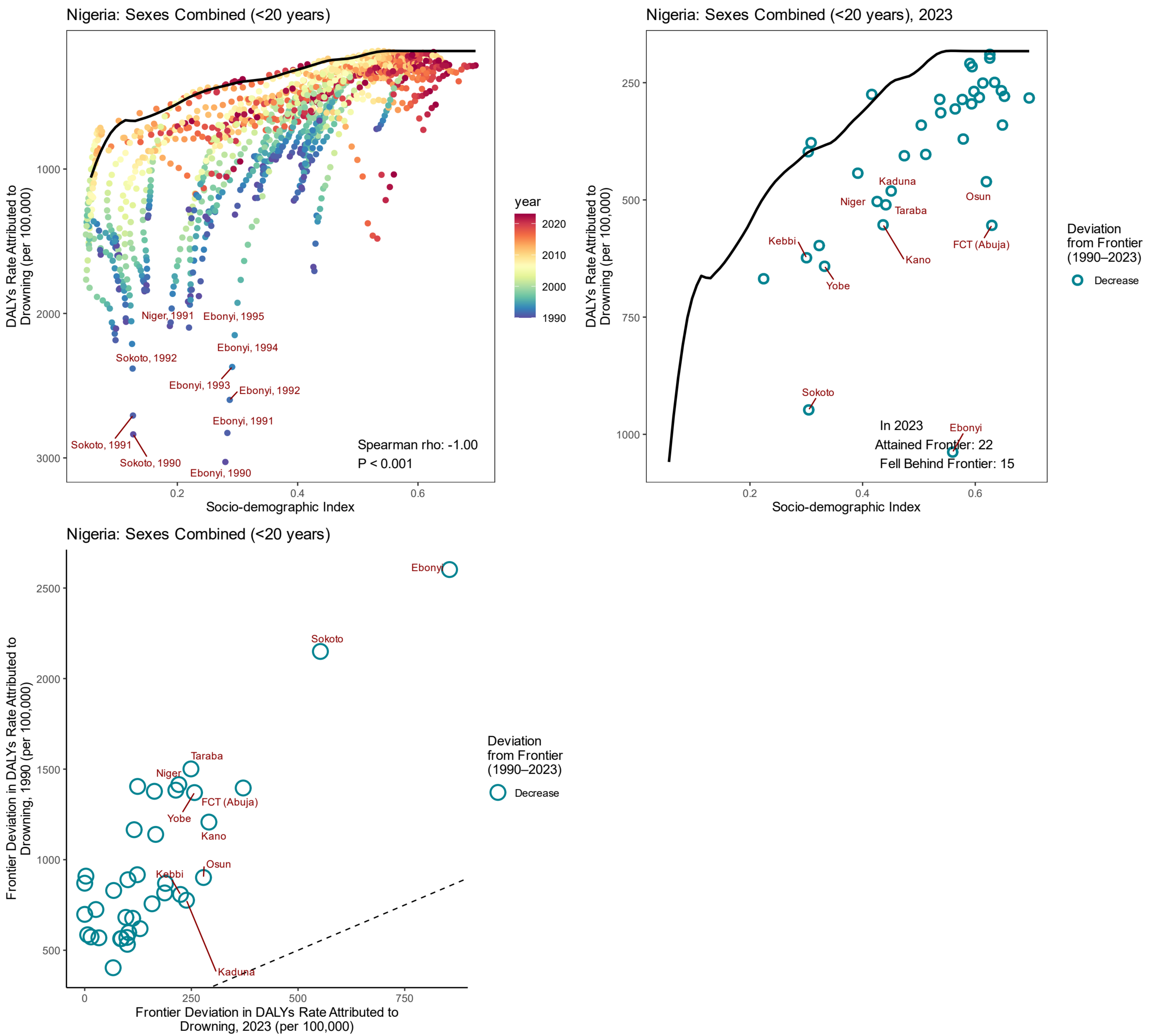

Nigeria: Sexes Combined (<20 years)
DALYs Rate Attributed to Drowning (per 100,000)
Socio-demographic Index
year
2020
2010
2000
1990
Niger, 1991
Ebonyi, 1995
Ebonyi, 1994
Sokoto, 1992
Ebonyi, 1993
Ebonyi, 1992
Ebonyi, 1991
Sokoto, 1991
Sokoto, 1990
Ebonyi, 1990
Spearman rho: -1.00
P < 0.001
Nigeria: Sexes Combined (<20 years), 2023
DALYs Rate Attributed to Drowning (per 100,000)
Socio-demographic Index
Kaduna
Osun
Niger
Taraba
Kebbi
Kano
FCT (Abuja)
Yobe
Sokoto
Ebonyi
In 2023
Attained Frontier: 22
Fell Behind Frontier: 15
Deviation from Frontier (1990–2023)
Decrease
Nigeria: Sexes Combined (<20 years)
Frontier Deviation in DALYs Rate Attributed to Drowning, 1990 (per 100,000)
Frontier Deviation in DALYs Rate Attributed to Drowning, 2023 (per 100,000)
Ebonyi
Sokoto
Taraba
Niger
FCT (Abuja)
Yobe
Kano
Osun
Kebbi
Kaduna
Deviation from Frontier (1990–2023)
Decrease

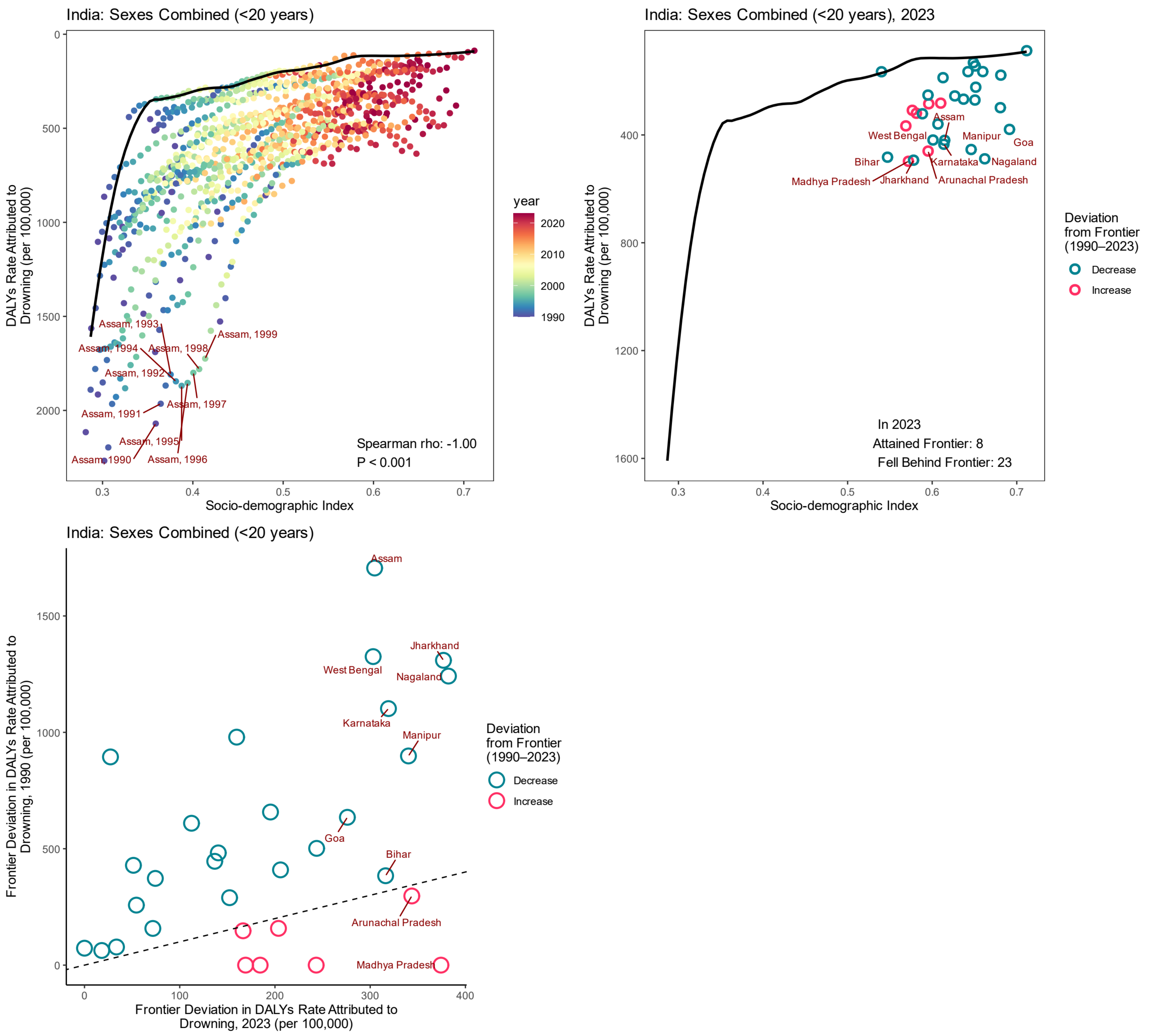

India: Sexes Combined (<20 years)
DALYs Rate Attributed to Drowning (per 100,000)
Socio-demographic Index
year
2020
2010
2000
1990
Assam, 1993
Assam, 1994
Assam, 1998
Assam, 1999
Assam, 1992
Assam, 1997
Assam, 1991
Assam, 1995
Assam, 1990
Assam, 1996
Spearman rho: -1.00
P < 0.001
India: Sexes Combined (<20 years), 2023
Assam
West Bengal
Manipur
Goa
Bihar
Karnataka
Nagaland
Madhya Pradesh
Jharkhand
Arunachal Pradesh
In 2023
Attained Frontier: 8
Fell Behind Frontier: 23
Deviation from Frontier (1990–2023)
Decrease
Increase
India: Sexes Combined (<20 years)
Frontier Deviation in DALYs Rate Attributed to Drowning, 1990 (per 100,000)
Frontier Deviation in DALYs Rate Attributed to Drowning, 2023 (per 100,000)
Assam
Jharkhand
West Bengal
Nagaland
Karnataka
Manipur
Goa
Bihar
Arunachal Pradesh
Madhya Pradesh
Deviation from Frontier (1990–2023)
Decrease
Increase
